# Comparison of Mechanical Properties of Natural Gut and Synthetic Polymer Harp Strings

**DOI:** 10.3390/ma11112160

**Published:** 2018-11-01

**Authors:** Nicolas Lynch-Aird, Jim Woodhouse

**Affiliations:** 1 The Old Forge, Burnt House Lane, Battisford, Suffolk IP14 2ND, UK; 2 Engineering Department, Cambridge University, Trumpington St, Cambridge CB2 1PZ, UK; jw12@cam.ac.uk

**Keywords:** polymer, nylon, fluorocarbon, gut, string, tuning, harp

## Abstract

The long-term mechanical behaviour of a number of fluorocarbon and gut harp strings has been examined, and the results compared with a previous study of rectified nylon strings. The stretching behaviour of the three materials was studied via different measures of the Young’s modulus; with test time scales on the order of weeks, minutes, and milliseconds. The strings were subjected to cyclic variations in temperature, enabling various aspects of their thermal behaviour to be investigated. The effects of humidity changes on gut strings were also examined. The behaviour of the fluorocarbon strings was found to be similar in many ways to that of the nylon strings, despite their different chemical formulation and significantly higher density. In particular, the faster measures of Young’s modulus were found to show an almost identical strong variation with the applied stress; while the thermal behaviour of both materials was largely determined by the balance between opposing effects associated with thermal contraction and thermal variations in the Young’s modulus. The gut strings showed some similarities of behaviour to the synthetic materials, but also major differences. All three measures of the Young’s modulus remained constant as the applied stress was increased. The gut strings were far more sensitive to changes in humidity than the synthetic materials, although some of the results, especially the thermal tuning sensitivity of the strings when held at constant length, displayed remarkable stability under changing humidity. The observed behaviour suggests very strongly that there is significant coupling between humidity-related changes in the linear density of a gut string and complementary changes in its tension.

## 1. Introduction

The strings of musical instruments like the violin, guitar or harp are made from a variety of materials. There are important issues to do with the sound and “playability” of these instruments, which depend on the mechanical properties of these strings. Furthermore, musicians are finely-tuned to small details of the behaviour of their instruments, so that quite subtle aspects of the string behaviour may be significant. Somewhat surprisingly, there is very little published data about the mechanical behaviour of strings, and what there is stops well short of the level of detail necessary to address serious musical concerns. This paper presents new data, which contributes to filling this gap.

The original motivation behind this study was to address a particular concern. Harp strings, whether of natural gut or synthetic polymer, are notorious for their sensitivity to their environment, to the extent that they may frequently become noticeably out of tune during the course of a performance. It was hoped that, if a sufficient understanding could be obtained of how these strings respond to changes in temperature and humidity, it might be possible to develop an automated system to make pre-emptive tuning corrections. Any such automated tuning system would also demand a sufficiently good understanding of the string’s Young’s modulus to ensure that the required tuning corrections could be made with the minimum number of iterations. Specifically, the tensile “tangent modulus” (the stress/strain ratio for small perturbations) of the string at its expected operating point is needed. Without that understanding, there is the risk that any tuning changes would be too small, and hence too slow, to keep the string in tune. Conversely, if the tuning changes were too large (which would result from using an estimate for the Young’s modulus that is too low), there is a danger of the tuning system becoming unstable.

This project began by studying the behaviour of centreless-ground (“rectified”) monofilament nylon harp strings. Results were published in a previous paper [1]. Compared to natural gut strings, one might expect that these synthetic strings should be relatively homogeneous along their lengths and also relatively consistent from one string to the next. The study involved in-depth measurements of the stretching behaviour of the strings and also of their response and sensitivity to changes in temperature. It proved necessary to examine stretching behaviour over a wide range of time scales: from weeks (to capture the long-term creep response of the strings), to minutes (to give the tangent modulus relevant to tuning changes), and right down to milliseconds (to capture the effect of string bending stiffness on overtone frequencies). Collecting this information is a slow process: each tested string typically needed to remain on the test rig for several months.

The fundamental frequency $f_{0}$ of a stretched flexible string is given to a good approximation by the well-established equation [2]:

(1) $$f_{0}=\frac{1}{2L_{V}}\sqrt{\frac{F}{\mu }}=\frac{1}{2L_{V}}\sqrt{\frac{\sigma }{\rho }}$$

where *F* is the string tension, μ is its linear density, $L_{V}$ is the vibrating length, ρ is the material bulk density, and σ=F/A is the stress, *A* being the cross-sectional area. In addition to investigating the variation in the string fundamental frequency, the study therefore also examined the changes in tension and linear density in response to temperature fluctuations. The tension variations were studied in further detail by measuring the thermal variation in the Young’s modulus, which in turn enabled estimates of the longitudinal coefficient of linear thermal expansion (CLTE) to be obtained.

However, rectified nylon is by no means the only choice for harp strings: fluorocarbon strings, made from polyvinylidene fluoride (PVDF) and commonly known as “carbon” strings, are becoming increasingly popular, while many harp players still prefer to use strings made from natural animal gut. This paper extends the previous work with a similar study into the mechanical and thermal properties of fluorocarbon and natural gut harp strings, and compares the behaviour of all three materials. The Young’s modulus is investigated over the same wide range of time scales, together with the effects of the string stretching on its density and working tension. This is followed by an examination of how the string frequency, tension, and linear density are affected by changes in temperature, and, to a lesser extent, by changes in humidity.

As already mentioned, there is very little published information available regarding the mechanical properties of synthetic or natural gut musical instrument strings. Even the weighty “Handbook of Materials for String Musical Instruments” [3] devotes only some 20 out of 950 pages to the mechanical properties of the kind of strings investigated here, and even then, most of that space is devoted to historical material rather than to mechanical measurements on modern strings. Some information is available regarding the tensile Young’s modulus and breaking strength of modern nylon strings, for example [4,5], but these values should be treated with caution. The tensile Young’s modulus of a viscoelastic material is a complex quantity that is expected to vary with the frequency or time scale of the method by which it is measured (see for example [6]). The previous study [1] showed that this was certainly the case for rectified nylon strings: values varied over an order of magnitude over the frequency range investigated. Moreover, for these nylon strings, the tensile Young’s modulus was found to vary significantly with the applied stress: the tangent modulus of a given string increased by roughly a factor of four as the applied stress increased from zero to the breaking stress. Any single estimate for the Young’s modulus of a nylon instrument string, as offered by earlier publications, would miss all this complexity.

With the exception of a study by Bell and Firth from 1986 [7], there is virtually no published data regarding the properties and behaviour of gut instrument strings, and absolutely no relevant information appears to be available for fluorocarbon strings. Bell and Firth’s paper explored the bulk density, twist ratio (the ratio of the twist period along the length of the string to its diameter), tensile strength, and Young’s modulus of quite a large number of gut strings (54 strings covering eight diameters from 0.52–2.51 mm), but did not examine the effects of changes in temperature and humidity. Furthermore, the tensile testing was conducted by applying a slowly increasing tension to each string. Gut, like nylon, creeps when stretched, and it could be expected that the Young’s modulus values obtained in this way would, therefore, be lower than the tangent modulus that would apply if the strings had first been allowed to finish creeping at any given test tension. The present study has examined fewer strings than in Bell and Firth’s study, but the time has been taken to allow the strings to finish creeping prior to each set of tests. A wider range of properties has then been measured, and information obtained over a wide range of time scales.

## 2. Methods and Materials

The tests were conducted using the same methods and protocols as those developed for testing nylon strings [1]. The test rig built for this purpose consisted of a solid hardwood baseboard of well-seasoned Canadian rock maple along which a single test string was mounted approximately 2 cm above its surface. A motorised winding shaft at one end of the string enabled length adjustments to be made with an accuracy of better than ±0.008 mm. The other end of the string was connected to a load cell (Novatech F256EFR0KN 40 kg) with an operating range of 0–300 N and accurate to within ±0.2 N. Between the winding shaft and the load cell, the string passed over two bridge pins mounted in ball bearings. These bridge pins were mounted perpendicular to each other (one parallel to the baseboard and one vertical) so that the effective vibrating length of the string was not affected by the direction in which it was plucked [8]. A motorised plucker, which used a guitar plectrum to pluck the string, was positioned mid-way between the bridge pins, together with a microphone and a digital temperature and humidity sensor. The latter had a nominal accuracy of ±0.3 °C and ±1.8% RH (relative humidity). The whole assembly was contained within a wooden chamber with a removable Perspex front panel. Heating was provided using combinations of 100-W and 150-W incandescent light bulbs, and a water reservoir was included to maintain the humidity levels within the chamber.

The test rig could therefore directly measure the string frequency, tension, and any tuning length adjustments (the lengths of string wound onto or off the winding shaft), together with the temperature and relative humidity. The string diameter was periodically measured using the same manual micrometer used for the initial string measurements (see Table 1) with a resolution of 0.01 mm. The string linear density was obtained from the measured frequency and tension using Equation (1), with the bulk density in turn obtained using the measured diameter. A more detailed and comprehensive description of the test rig is given in [1], together with the various equations used for the derived quantities.

The fluorocarbon strings were tested on the same rig as the earlier nylon tests, while a second test rig of the same design and capabilities was constructed to enable the gut strings to be tested in parallel. For each target fundamental test frequency (see Table 1), the rig was run in its constant-frequency mode until the string had settled (as far as could be determined using the creep-monitoring approach described in [1]). A Young’s modulus test was then run, using a 30-min cycle of tension modulation around a base tension measured when the string was tuned to the target frequency. Tests were also run with the string held at constant length (no tuning adjustments) and at constant tension. During all of these tests, a 24-h heating cycle was applied. As with the nylon strings, each of the fluorocarbon and gut strings was typically mounted on its test rig for several months.

The tensile Young’s modulus was measured over the same three time scales as for the previous study [1]: a slow measure $E_{S}$ was obtained from the gradient of the stress-strain response obtained across the full set of target fundamental test frequencies. An intermediate measure $E_{T}$ was obtained as described above using a tension modulation cycle with a period of about 30 min. Finally a fast measure $E_{B}$ was derived from the string bending stiffness, which in turn was obtained by analysing the deviation of the string overtones from an exact harmonic series [8]. The test rig control program included a facility to analyse the pattern of overtone frequencies deduced from recorded pluck responses automatically [1]. This approach worked well with the nylon and fluorocarbon strings, but gave rather poor results with the gut strings. Manual pluck responses from the gut strings, recorded using the wire-break method [8], were therefore analysed instead. For the measurements of $E_{T}$ (all three materials) and the measurements of $E_{B}$ (nylon and fluorocarbon only), the application of a heating cycle during the tests meant that the thermal sensitivity in these measures of the Young’s modulus could also be studied.

The earlier study of nylon strings [1] also included some dynamic mechanical analyser (DMA) tests to further explore the variation of the Young’s modulus with the test frequency. These DMA tests were not run for the fluorocarbon strings, and an attempt to test samples of gut strings was unsuccessful: the test used a three-point bend configuration with no tension in the string, and the bond between the strands of the gut string failed at the localised contact points in the clamping rig. The loss of this test procedure was not thought to be important: in the earlier tests, the DMA results simply served to confirm the viscoelasticity interpretation of the other results over different time scales. As will be seen, qualitatively similar behaviour at these different time scales was found in the new tests, and there is no reason to doubt that viscoelasticity is again the underlying explanation.

During most of the tests, efforts were made to maintain a fairly consistent humidity level within the test chambers, of around 60% RH at 20 °C, while the temperature was varied. These efforts did not, however, completely shield the test chambers from the effects of variations in the ambient humidity levels outside the test chamber, which changed with the weather. Care had also to be taken not to overheat the strings (especially the gut strings), and in general, the temperature inside the test chambers was kept below 40 °C.

Even quite small changes in the ambient humidity conditions were found to have a noticeable effect on the properties of the gut strings. To examine more specifically the effects of changes in the overall (average) temperature and humidity levels, a number of additional tests were run with some of the gut string sections (G5b and G5c; see Table 1). In these more extended tests, which were run for durations of 2–8 weeks, the string was tuned to a target frequency and then left without making any further tuning adjustments, while the overall temperature and humidity levels within the test chamber were varied. The average temperature range obtained during these tests was 4–29 °C, with an average absolute humidity (AH) range of 4–29 g/m${}^{3}$.

### Strings Tested

Tests were conducted on four fluorocarbon harp strings from the Savarez Alliance range for harps [9], and three gut pedal harp strings from Bowbrand (Norfolk, UK) [10]. For two of the gut strings, the tests were repeated on two different sections of the same string. The previously tested nylon strings were all from the Bowbrand “Pedal Nylon” range. The thinner fluorocarbon strings in the Savarez Alliance range are monofilaments, like the nylon strings tested previously. The fatter strings, however, have a more complex construction, with a number of thin filaments wound around a central core of the same material. Examples of both types of string were included in these tests.

The gut string manufacturing process employed by Bowbrand takes many weeks and is highly manual. Strips of washed cow gut are first gathered into bundles: the number of strips varies both with the required string gauge and the herd from which the gut has been sourced. The bundles are treated in a chemical bath, which includes a fixative, and then twisted together. The twisted bundles are dried slowly under controlled temperature conditions until they no longer display a tendency to untwist when released at one end. Finally the dried bundles are centreless ground to the required gauge, and varnished with several coats of clear or coloured varnish. The final string gauge produced from an initial bundle of gut strips may vary from the intended gauge due to variations in the thickness of the individual gut strips; the chosen bundle counts are only a rough guide. Consequently, the number of gut strips can vary between strings of the same gauge. The strings used by Bell and Firth were provided by Salvi harps and were also made from cow gut [7]. They would not, however, have been manufactured by Bowbrand since, at that time, Bowbrand was using sheep gut for their harp strings; they had yet to make the transition to cow gut.

Table 1 lists the strings, with their unstretched diameters and densities measured prior to testing. The string diameters were measured using a manual micrometer with a resolution of 0.01 mm. Multiple measurements were taken along the length of each string and averaged to give the values shown in Table 1. These strings were part of a larger set, used in a number of studies. When each string was first weighed and measured, it was assigned an identification number, which is shown in the first column of Table 1. Many of the strings were long enough to provide multiple sections for testing. Where this was the case, the string number was supplemented with a suffix letter to differentiate the sections. These original identification numbers may seem a little illogical in the context of the present study, but they have been retained for consistency with reports on other tests. These numbers are used consistently in the summary datasets accompanying this paper and in the much larger comprehensive datasets available at https://doi.org/10.17863/CAM.9018 (nylon) and https://doi.org/10.17863/CAM.31653 (fluorocarbon and gut). Within this paper, an additional prefix ‘N’ has been added to the identification numbers for the nylon strings, to help distinguish them from the fluorocarbon and gut strings. The note shown for each string indicates its intended usage on the harp and is given using the piano scale. The corresponding string number on the harp string numbering scale is shown in brackets.

Each string section was tested over a range of different target fundamental frequencies, listed in Table 1. These included frequencies corresponding to the expected operating points of each string on the harp. Since the test rigs had fixed vibrating lengths, while the strings on the harp are all of different lengths, the test frequency to be used at each string’s intended operating point was calculated by multiplying the intended frequency for that string by the ratio of the string vibrating length on a pedal harp to that on the test rig. The harp measured for this purpose was a Russian-made Elysian Cecilia 46 pedal harp. The slight differences in the test frequencies used for the gut strings were due to a small difference in the string vibrating lengths on the two test rigs: 500 mm on the test rig used for the nylon and fluorocarbon strings; 498.5 mm on the test rig used for the gut strings.

**Figure 1:**
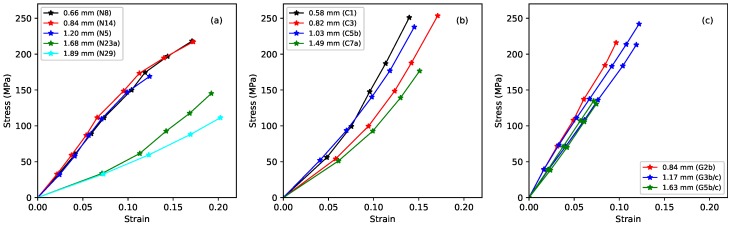
Long-term stress-strain plots at the base temperature of 20 °C, with the strings settled at each point (using the creep-monitoring approach described in [1]), for (**a**) nylon, (**b**) fluorocarbon, and (**c**) gut.

Fluorocarbon strings have a significantly higher bulk density than gut and nylon strings. One consequence of this is that, in order for the corresponding strings for a given note to have approximately the same tension, and thus the same “feel” (defined by Firth as $4F/L_{V}$, where *F* is the string tension and $L_{V}$ is its vibrating length [11,12,13]), fluorocarbon strings have smaller diameters than their gut and nylon counterparts (see Equation (1)). It follows that fluorocarbon strings operate at a higher stress level for a given note, compared to gut and nylon strings. This resulted in the tests being completed at fewer target test frequencies before the fluorocarbon strings broke, even though their breaking stress was typically higher than for gut or nylon (see Figure 1, for example).

## 3. Results

### 3.1. Results for Young’s Modulus

Figure 1 shows the long-term relation between the measured stress and strain. The layout of this figure will be followed in many subsequent comparative plots: the leftmost plot reproduces earlier results for rectified nylon strings; the centre plot shows the fluorocarbon strings; and the rightmost plot shows the gut strings. Identical axis scaling is used for all three subplots to facilitate direct comparisons between the three string types. Each plotted point here represents the state of a string, at the base temperature of 20 °C, when it had settled at the chosen stress level (using the creep-monitoring approach described in [1]). The slopes of the curves define the ‘slow’ Young’s modulus $E_{S}$. This quantity includes a contribution from the creep extension, accumulated over a period of several weeks.

As previously reported, the nylon strings split into two distinct groups, corresponding to a difference in their densities (see Table 1). This presumably points to a difference in their chemical make-up; however, the strings were purchased through a normal retail outlet, and it has not proven possible to track down the detailed chemical formulation. The stiffness of the thinner, lower-density nylon strings fell as the strain increased; while the stiffness of the thicker, higher-density strings increased. The fluorocarbon strings showed similar behaviour to the thicker, higher-density nylon strings, albeit with consistently higher stiffness. While the fluorocarbon responses extended to higher stress levels than the corresponding nylon strings, they tended to break at lower overall strain levels. There was no obvious distinction between the behaviour of the monofilament and wound strings. The responses for the gut strings showed a similar overall stiffness to the fluorocarbon strings, but were strikingly linear with very similar gradients (fitting lines through the origin to each of the gut responses gave $r^{2}$ values in excess of 0.999 in every case, while fitting a single line to the data for all five gut string sections resulted in a combined $r^{2}$ value of 0.992). This is in clear contrast to the curvatures shown by the synthetic materials.

**Figure 2:**
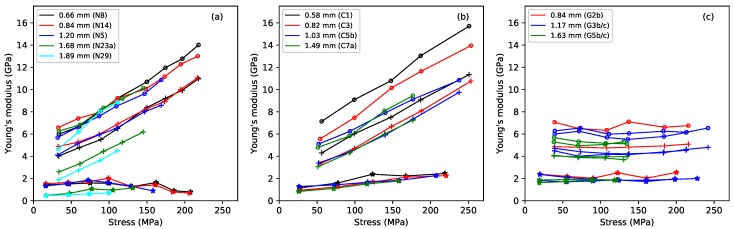
Young’s modulus at the base temperature of 20 °C plotted against the applied stress, for (**a**) nylon, (**b**) fluorocarbon, and (**c**) gut. Results are shown for the three measurement approaches: $E_{B}$ (o) from bending stiffness, $E_{T}$ (+) from modulation of string tension and $E_{S}$ (*) from the slope of the long-term stress-strain plots shown in Figure 1.

Figure 2 shows the base-temperature Young’s modulus plotted against the applied stress, for the three different measurement methods: the slow modulus $E_{S}$ calculated from the slopes of the lines in Figure 1; the modulus $E_{T}$ measured using a tension-modulation cycle with a period of around 30 min; and the modulus $E_{B}$ calculated from the string bending stiffness [1] and thus based on stress-strain variations with a millisecond time scale. For any given string (indicated by colour in the plot), $E_{S}$ had the lowest value; $E_{T}$ came next, and the modulus $E_{B}$ was highest. This pattern is familiar: the Young’s modulus of a viscoelastic material generally increases as the time scale of the measurement method reduces, or equivalently as the frequency increases [6,14]. The slow deformation described here as “creep” only plays a major role in $E_{S}$, because the measurements of $E_{T}$ and $E_{B}$ were too fast for this creep to have much effect.

The fluorocarbon strings showed behaviour in many ways similar to that of the nylon strings, but with some slight differences. The $E_{S}$ responses rose steadily with the applied stress, in accordance with the stress-strain results of Figure 1, and at a somewhat faster rate than the higher-density group of nylon strings (strings N23a and N29 in Figure 2a). The $E_{T}$ and $E_{B}$ responses rose in a very similar linear fashion as the stress was increased, and with much the same range of values and gradients as the nylon strings. As might be expected from the differences in their construction, and in contrast to the fairly small spread in the $E_{T}$ responses, the two wound fluorocarbon strings gave distinctly lower $E_{B}$ responses, compared to the monofilament strings. Over-wrapped string construction, whether with metal wire or, as here, with the same material as the core, is used deliberately to reduce the bending stiffness of thicker and heavier strings.

The gut strings showed very different behaviour to the synthetic materials. Although the same trend $E_{S}<E_{T}<E_{B}$ was seen, all three measures of Young’s modulus remained remarkably constant as the applied stress, and hence strain, was increased. In the case of the thinner strings (G2b, G3b, G3c), this remained true even up to the point where the string broke. One consequence of this difference in behaviour is that, at their intended operating points, gut strings will often have a lower bending stiffness than the equivalent nylon or fluorocarbon strings, potentially leading to lower levels of inharmonicity in the string overtones [12]. The Young’s modulus for natural gut instrument strings is generally reported to be about 5.5–6.5 GPa [4], but it would seem that the situation is more complicated than this. This range of values is closest to the observed values of the Young’s modulus obtained from the bending stiffness, perhaps indicating that the published values were obtained using a similar high-frequency measurement technique.

The behaviour seen in Figure 2 suggests that, contrary to what one might have guessed, the gut strings can be characterised much more simply than the synthetic strings. Gut appears to behave as a linear viscoelastic material over the entire stress range of these tests. The Young’s modulus varies with frequency, but it does not exhibit the strain hardening, or more properly “strain stiffening”, shown by both nylon and fluorocarbon strings. This stiffening implies that the microstructure is being significantly and progressively modified by the imposed strain. The previous study [1] suggested a mechanism: the change might correspond to progressive straightening of the nylon molecules, from an initially random state in the polymer melt. The behaviour could be similar in both nylon and fluorocarbon strings, explaining why their $E_{B}$ and $E_{T}$ responses have such similar gradients (about 40 in both cases).

The structure of gut strings is different in two distinct ways, one microscopic and one macroscopic. Firstly, the structural component of gut is collagen. Collagen molecules consist of a triple helix, whose structure is well understood [15,16]. The biological process of growing gut fibres will presumably result in well-ordered chains, so there may not be the same scope as with the synthetic materials for additional alignment as a result of strain once the string is under tension. Secondly, the gut strings have a wound construction without a central core, and this will presumably influence the stress-strain behaviour. Some of the fluorocarbon strings also had a wound construction, but with a central core that may have dominated their responses to changes in the applied stress and strain, making them behave more like the monofilament strings in this respect.

There is another consequence of the more complicated behaviour of the synthetic strings, compared to the gut strings. The increase in the faster measures of Young’s modulus ($E_{T}$ and $E_{B}$) with the applied stress should correspond to a falling off in the elastic stretching; while the relatively constant levels of $E_{S}$ suggested that the proportion of creep deformation was increasing. The synthetic strings seem to become more susceptible to creep at higher stresses, perhaps because the cross-links between polymer molecules are more readily overcome. No comparable mechanism seems to operate in the gut strings. It would appear that the gut and synthetic strings were behaving very differently in terms of how both their “elastic” and “plastic” stretching varied with the applied stress. It seems possible that this different behaviour might have some influence on a player’s sense of the “feel” of strings.

The previous study showed that the spread in the $E_{T}$ results from the nylon strings could be matched quite well by fitting a linear function in both the applied stress and bulk density. However, given the much higher density of the fluorocarbon strings the same density term cannot be used for this material, and the spread of densities among the tested fluorocarbon strings was too small to justify further exploration at this stage.

**Figure 3:**
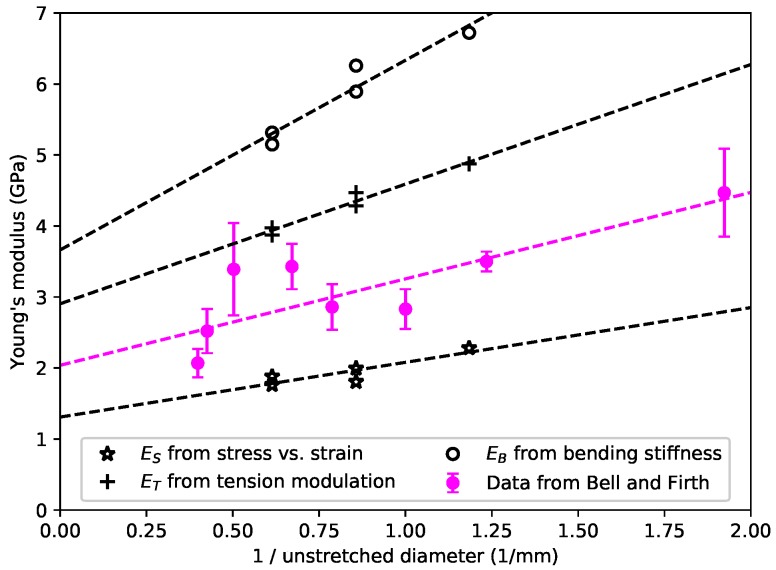
The average Young’s modulus values for the gut string sections from Figure 2c, plotted against the inverse of the unstretched string diameter. The equivalent results obtained by Bell and Firth [7] are shown for comparison. Bell and Firth tested multiple strings at each diameter, and the fitted line was obtained using a weighted linear regression analysis to reflect this. The vertical bars span ±1 standard deviation about the mean Young’s modulus for each string diameter.

Unlike the nylon strings, no obvious dependence on the string density was observed with the gut strings, but there did appear to be a correlation between all three measures of Young’s modulus and the inverse of the string diameter or cross-sectional area. Figure 3 shows the average Young’s modulus values from Figure 2c, plotted against the inverse of the diameter of the unstretched strings. The equivalent data from the study made by Bell and Firth [7] are also shown for comparison: they appeared to show a similar variation with the string diameter and/or cross-section. Their values lay between the $E_{S}$ and $E_{T}$ values obtained in this study, as would perhaps be expected from the way in which Bell and Firth conducted their measurements. Their methodology was similar to the $E_{S}$ measurements here, based on stress-strain tests, but conducted over a much shorter time scale so that the effective stiffness was pushed towards the $E_{T}$ values. Studies currently under way to explore the effects of varying the string twist ratio suggest that the behaviour shown in Figure 3 may actually be due to different gauge strings being twisted to different extents during manufacture.

**Figure 4:**
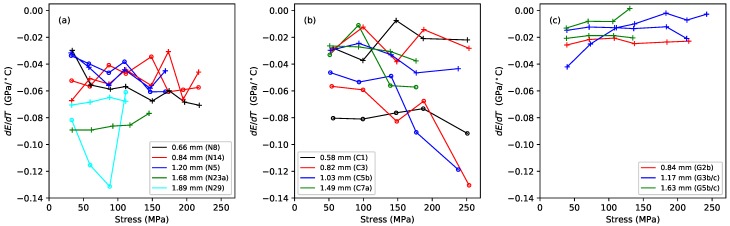
The rates of thermal variation in the Young’s modulus results obtained using tension modulation (+), and from the string bending stiffness (o), about the base temperature of 20 °C, for (**a**) nylon, (**b**) fluorocarbon, and (**c**) gut.

Figure 4 shows the gradients, plotted against the applied stress, of lines fitted to the Young’s modulus versus temperature responses, for the Young’s modulus values obtained using tension modulation (+) and from the string bending stiffness (o). For the latter, data were only obtained for the nylon strings tested after the automated bending stiffness analysis capability had been added to the test rig control program [1]. As explained in Section 2, this approach did not give good results for the gut strings, so only the thermal sensitivity in the $E_{T}$ values is shown for these strings. The noise level apparent in these results was much higher than in previous plots, as a result of the accumulated inaccuracies implicit in the estimation of this gradient. This noise would, of course, be reduced if a larger number of strings of each type could be tested, but this was not practical given the length of time occupied by each individual test sequence, as explained earlier. Nevertheless, some general trends can be seen with a moderate degree of confidence. In very nearly every case, the values are negative: the Young’s modulus fell as the temperature increased, making the string easier to stretch.

For each of the three materials, the thermal sensitivity in $E_{T}$ appeared to remain at a fairly consistent level as the stress was varied. There was some inconsistency between the results for one of the G3 string sections (G3b) and all other tested strings, including another section of that same string (G3c). The somewhat anomalous behaviour of string G3b, which may have been due to some material inconsistency somewhere along its length, was part of the motivation for repeating the tests with strings G3c and G5c; along with the desire simply to gather more data. It is interesting that, other than the anomalous behaviour of string G3b, the gut results showed a much lower degree of variation than either of the synthetic materials.

The results for the Young’s modulus derived from the string bending stiffness, $E_{B}$, were less clear. For nylon, the thermal sensitivity again appeared to remain fairly constant as the stress was varied, although string N29 was something of an outlier. For the fluorocarbon strings, however, the thermal sensitivity appeared to become increasingly negative as the stress was increased.

### 3.2. Results for Density and Tension

**Figure 5:**
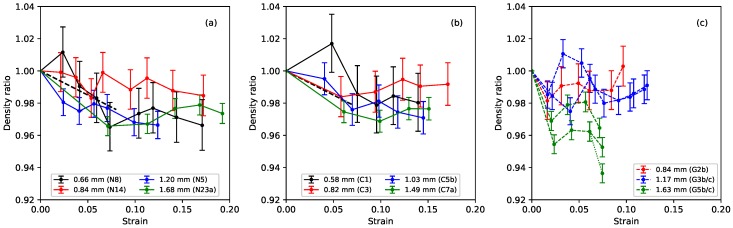
The ratio of the bulk density of the stretched strings to that of the unstretched strings, plotted against the strain, for (**a**) nylon, (**b**) fluorocarbon, and (**c**) gut. The error bars indicate the range of values that could be expected due to the resolution limit of the manual micrometer used to measure the string diameter. The dashed lines in Plots (**a**,**b**) show the expected reduction in the density ratio for small values of strain [1].

Figure 5 shows the ratio of the measured bulk density of the stretched strings to their unstretched bulk density, plotted against the strain. The stretched density values here were calculated from the linear density at a base temperature of 20 °C, obtained from the constant-length tests, and the string diameter, which was measured using a manual micrometer with a resolution of 0.01 mm. With the string mounted in the test rig, the diameter could only be measured in a few places, and could therefore not be determined as accurately as when the string was first measured prior to testing (when multiple measurements were taken to reduce the measurement error and take better account of any variations along the string length). The error bars in Figure 5 show the range in the density ratio values that could be expected given an error range of ±0.005 mm in the measured string diameter. In this case, although the results were very noisy, there was a very clear expectation of the general pattern based on theory and previous tests in other contexts where elasticity and plasticity were both present: at low strain, density is expected to decrease with a slope determined by Poisson’s ratio (shown as black dashed lines in Figure 5a,b). At larger strain, when plastic deformation takes over, the normal expectation would be volume-conserving deformation leading to roughly constant density. As observed in the previous study [1], the results for nylon, reproduced in Figure 5a, were entirely consistent with this behaviour.

The fluorocarbon strings showed very similar behaviour to the nylon strings, with the density ratio falling initially as expected for small values of strain, before settling to a more or less constant level: notice the very narrow range on the vertical axes in the plots (the nylon string N29 from the previous study was something of an outlier with a larger fall in its density ratio to about 0.92 [1], and so has not been included here). The results for the gut strings were more varied, but, overall, it would appear that the string densities again fell slightly on initial stretching (more so in the case of the thickest string sections, G5b and G5c, than for the others) and then, within the accuracy of these measurements, remained essentially constant as the strings were stretched further. It also appears that the initial density reduction proceeded more quickly with the gut strings, but stopped at a lower strain level than with the nylon and fluorocarbon strings in some cases.

A general comment that will come as no surprise to players is that the gut strings were found to be noticeably sensitive to changes in the ambient humidity levels, while the nylon and fluorocarbon strings were largely unaffected. These changes directly affected the linear density, and hence bulk density, values obtained. The successive data points for each of the gut strings were typically recorded two to three weeks apart, so the results contained a contribution from changes in the prevailing weather conditions. The dotted fork at the end of one of the green lines (for string G5c) in Figure 5c gives a particularly clear example: the string behaviour changed significantly over the course of just a few days during a single test (see Section 4). Dashed lines, rather than solid lines, have been used in this and other plots of the gut results to alert the reader to potential uncertainty where it is expected that such changes in the ambient conditions could have made a significant impact.

**Figure 6:**
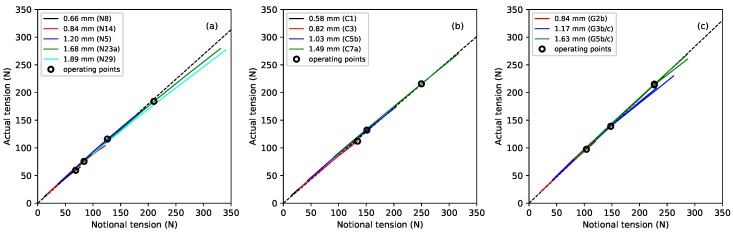
The measured tension of the strings versus the notional tension calculated using the unstretched linear density, for (**a**) nylon, (**b**) fluorocarbon, and (**c**) gut. Black circles show the results at the expected operating points. The dashed lines were fitted through these points and the origin for each material.

As a string is brought up to tension, to tune it to the required frequency, its linear density will necessarily be reduced (as string mass is removed from the vibrating section). From the density ratio results above, there will also be a corresponding reduction in the string diameter. Without detailed knowledge of the stretching behaviour of the string, including both the elastic and plastic (creep) stretching, it is difficult to predict the working tension once the string has finished creeping. Figure 6 shows the measured tension $F_{0}$ of the different strings, at the base temperature of 20 °C, plotted against the notional tension $F_{N}$ calculated from Equation (1) using the unstretched linear density. It can be seen that the actual tension fell further below the notional values as the tension increased (due to the progressive reduction in linear density). Linear regression analysis was used to fit lines through the operating points, shown as black circles, for each material. The lines were constrained to go through the origin and gave a very good fit ($r^{2}>0.999$) in all three cases. The gradient of the fitted line for the fluorocarbon strings, at 0.860, was lower than that previously obtained for the nylon strings (0.896), while the gradient for the gut strings was somewhat higher at 0.943. This suggests that, as a rule of thumb, the working tension of a musical string, on the harp at least, will be about 10% lower than the notional value (obtained from the unstretched linear density) for nylon, 14% lower for fluorocarbon, but only 6% lower for gut.

### 3.3. Results for Thermal Tuning Sensitivity

**Figure 7:**
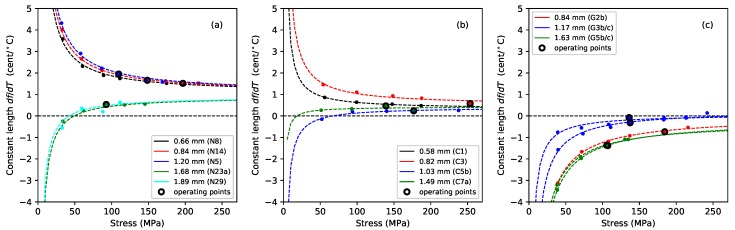
The thermal tuning sensitivity for the strings held at constant length with no tuning adjustments, for (**a**) nylon, (**b**) fluorocarbon, and (**c**) gut. Black circles show the results at the expected operating points. The dashed lines were fitted using linear regression analysis, with 1/stress as the explanatory variable.

Tuning deviations are conveniently expressed in cents (¢), there being 1200 ¢ in an octave [17]:

(2) $$\delta f_{¢}=1200\log_{2}\left(\frac{f}{f_{0}}\right)\;¢$$

where *f* and $f_{0}$ are the actual and desired frequencies, respectively. Figure 7 shows the thermal tuning sensitivity, in ¢/°C, for strings held at constant length with no tuning adjustments. All three materials showed a strong correlation between the tuning sensitivity and the inverse of the applied stress. The previous study of nylon strings [1] found that the thermal tuning sensitivity could be fitted rather well by an expression in the inverse of the stress and the inverse of the tension. The lower-density group of nylon strings consistently went sharp as the temperature rose, and their tuning sensitivity fell as the stress was increased. In contrast, the higher density nylon strings showed a tendency to go flat at low stress levels, but sharp at higher stress levels. Different regression fits were therefore required for the two groups of strings. The dashed lines in Figure 7a were plotted using these fitted expressions.

The fluorocarbon strings showed a similar pattern of behaviour to the nylon strings, but this time the distinction was between the monofilament strings, which consistently went sharp as the temperature rose, and the wound strings, which generally went sharp, but showed a tendency to go flat at low stress levels. The gut strings, however, nearly always went flat as the temperature was increased. For both nylon and fluorocarbon, the variation in behaviour with string diameter suggests that where a thin and thick string are used alongside each other, there is the possibility that one may go sharp as the other goes flat. Such behaviour has been directly observed on an octave pair of strings on a lute, strung with a wound fluorocarbon string alongside a thinner monofilament nylon string.

The degree of thermal tuning sensitivity of the gut and nylon strings was broadly similar, varying at rates of up to about 2 ¢/°C at their intended operating points. The thermal tuning sensitivity of the fluorocarbon strings was somewhat lower at less than 1 ¢/°C. It is generally accepted that the minimum perceptible change in pitch of a musical note is around 3 ¢, though this varies with the pitch and complexity of the sound [18]. Hence, even a modest change in temperature could be expected to lead to noticeable changes in the string pitch in many cases. This phenomenon, familiar to harpists, was the original motivation for this study, in the hope of being able to design a tuning compensator to offset the problem [19].

There was insufficient data to attempt a multiple regression analysis for the fluorocarbon and gut strings, similar to that undertaken for the nylon strings, so the dashed lines in Figure 7b,c were plotted using a separate linear regression analysis for each string, with the inverse of the applied stress as the explanatory variable. The fits for strings C1, C3, and C5b were fairly good with $r^{2}$ values of 0.99, 0.93, and 0.92, respectively. The fit for string C7a was not so good however, with a low $r^{2}$ value of just 0.28. The fits for the gut strings were particularly good for the thinnest and thickest strings (G2b, G5b, G5c) with $r^{2}\geq 0.998$ in each case, while the intermediate string sections, G3b and G3c, showed $r^{2}$ values of 0.97 and 0.87, respectively. There was also a high degree of consistency between the results for the thinnest string (G2) and the two sections of the thickest string (G5), despite string G5 being approximately twice the diameter of G2, and with the tests being run in three different years (Table 1). In contrast, the results for the two sections of string G3 were not particularly consistent, either with each other or with the rest of the group. It would appear that gut strings operating at higher stress levels (corresponding to the higher notes on the harp) should display lower thermal tuning sensitivities. In practice, though, other variations in the string, as exemplified here by string G3, may be more significant in determining the relative performance of the strings.

**Figure 8:**
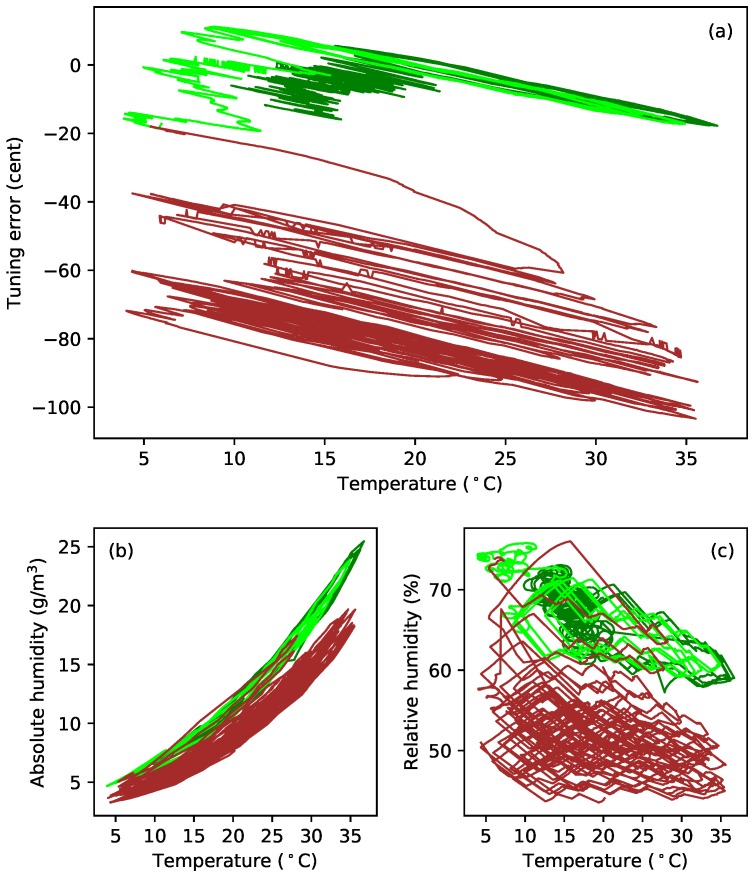
(**a**) The tuning error, (**b**) the absolute humidity, and (**c**) the relative humidity, all plotted against temperature, for the extended tests run with strings G5b (green) and G5c (lime) following completion of the initial series of tests at the last target fundamental frequency of 324 Hz. The brown sections show the responses for string G5c when the water reservoir was subsequently removed and heating reapplied.

Figure 8a plots the tuning error (in cents) against temperature during extended tests with gut strings G5b and G5c. After running the constant-length tests at the last target frequency (324 Hz; see Table 1), the heating was switched off, and the behaviour of these strings was simply monitored as the ambient temperature and humidity levels varied (Figure 8b,c). In the case of string G5c, the water reservoir, for maintaining the humidity levels in the test chamber, was subsequently removed, and the heating restarted, resulting in the brown sections of the responses for this string. The reasons for doing this are explained below (Section 4), but the feature to note here is that the gradients of the tuning error versus temperature responses remained remarkably constant despite significant changes in the overall temperature and humidity levels. This seems to be somewhat at odds with the general sensitivity of gut strings to environmental changes, explored in more detail in Section 4.

**Figure 9:**
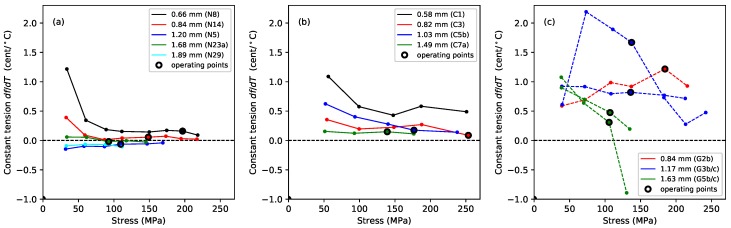
The thermal tuning sensitivity for the strings held at constant tension, for (**a**) nylon, (**b**) fluorocarbon, and (**c**) gut. Black circles show the results at the expected operating points.

Figure 9 shows the thermal tuning sensitivity when the strings were maintained at constant tension. The thermal variation in the string linear density was now the dominant influence. Note that holding the tension constant reduced the sensitivity to tension-related thermal effects significantly, but it did not remove them entirely: as the temperature rose and the string relaxed or contracted (see below), it had to be wound onto or off the winding shaft to compensate, thereby contributing additional changes to the linear density.

The thermal tuning sensitivities of the nylon strings were greatly reduced compared to the constant-length results seen in Figure 7a, with the sensitivities at the string operating points all smaller than 0.5 ¢/°C and often less than 0.3 ¢/°C; albeit with the thinner strings going slightly sharp as the temperature rose, while the thicker strings went slightly flat. The improvement was not as dramatic for the fluorocarbon strings, especially not for string C1, but all the fluorocarbon strings now consistently went sharp as the temperature rose. The overall form of the responses from the fluorocarbon strings was similar to that for the nylon strings, with the tuning sensitivity initially falling as the applied stress was increased and then settling at a more or less constant level.

In contrast to both kinds of synthetic string, the behaviour of the gut strings in this test was much more erratic. The gut strings now generally went sharp as the temperature was increased, but, rather than improving the tuning stability, holding the strings at constant tension actually made things worse in some cases. In striking contrast with the results from the constant-length tests shown in Figure 7c, the gut string responses shown in Figure 9c are neither smooth, nor particularly consistent between the different strings. The results for the two sections of string G5 were somewhat consistent with each other, but the results for the two sections of string G3 were very different. Given that changes in the linear density were now the dominant effect, and recalling the earlier remarks regarding sensitivity to changes in the prevailing weather conditions, these gut responses have again been plotted with dashed lines as a warning against taking the erratic curves at face value.

### 3.4. Variations in Tension and Linear Density

In accordance with Equations (1) and (2), the thermal tuning sensitivity, around a base temperature $T_{0}$, of a string held at constant length would be expected to be:

(3) $${\left.\frac{df_{¢}}{dT}\right|}_{L}\approx K\left(\frac{1}{F_{0}}\frac{dF}{dT}-\frac{1}{\mu_{0}}\frac{d\mu }{dT}\right)\;¢/{}^{\circ}C$$

where $K=600/\ln\left(2\right)$, and $F_{0}$ and $\mu_{0}$ are respectively the string tension and linear density at $T_{0}$. For convenience, the thermal variations in the string tension and linear density will be separated, and denoted λ and ψ respectively:

(4) $$\lambda =\frac{1}{F_{0}}\frac{dF}{dT}\;\;\mathrm{and}\;\;\psi =-\frac{1}{\mu_{0}}\frac{d\mu }{dT}.$$

**Figure 10:**
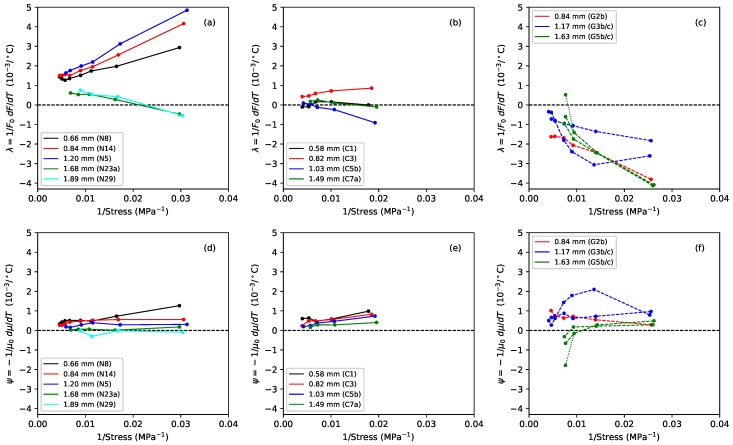
The thermal sensitivities in the string tension (top row) and linear density (bottom row) for the tests when the strings were held at constant length with no tuning adjustments, for (**a**,**d**) nylon, (**b**,**e**) fluorocarbon, and (**c**,**f**) gut. Adding these two sets of values together, and scaling by $K=600/\ln\left(2\right)$, gives the thermal tuning sensitivity responses shown in Figure 7.

Figure 10 shows the measured values of both quantities for the tests when the strings were held at constant length with no tuning adjustments. The thermal tension sensitivity values λ were obtained by fitting lines to the tension versus temperature responses. The corresponding values for ψ were similarly obtained from the linear density responses, the latter being calculated from the directly measured string frequency and tension responses [1]. Adding these two sets of values together, and scaling by *K*, gives the thermal tuning sensitivity responses shown in Figure 7.

The behaviour seen in Figure 8a for the G5b and G5c gut strings requires either that both λ and ψ remain largely constant, for a given applied stress, or that any change in one is offset by a corresponding change in the other. Similarly, the behaviour seen in Figure 7 for all three string materials would require either that both λ and ψ change linearly with the inverse of the applied stress or that any more complex change in one is offset by a corresponding change in the other. The responses in Figure 10 have been plotted against the inverse of the applied stress to explore this question.

For the nylon strings, both λ and ψ did appear to vary fairly linearly with the inverse of the stress, with the thermal tension sensitivity λ generally being the dominant term. It can clearly be seen how minimising the tension sensitivity, by holding the string at constant tension, will greatly reduce the overall thermal tuning sensitivity of a rectified nylon string. For the fluorocarbon strings, both λ and ψ again appeared to vary fairly linearly with the inverse of the stress, but now the contributions from the λ and ψ terms were more evenly matched. This explains why minimising the effects of the thermal tension variations did not provide such a marked improvement in the tuning sensitivity as it did for the nylon strings. It is interesting to note that the thermal sensitivity in the linear density ψ is of a very similar magnitude for both the nylon and fluorocarbon strings, with both materials losing mass (presumably through drying out) as the temperature increased. The main difference in the thermal behaviour of these two materials comes from their thermal tension sensitivity λ.

In contrast, the λ and ψ responses for the gut strings did not vary linearly, or even particularly smoothly, with the inverse of the applied stress, which strongly suggests that the thermal variations in the string tension and linear density must be coupled in some way. The values for ψ were generally, but not always, positive, indicating that in most cases the strings were again losing mass as the temperature rose. String G3b again showed a much higher degree of variation in its behaviour than the other section of the same string, or the thinner and thicker strings.

The reduction in linear density with an increase in temperature for all three materials, in most cases, would on its own tend to make the strings go sharp. For the nylon and gut strings, the thermal tension variation λ was generally the more dominant term, with the sign of this term largely determining whether the overall behaviour of the unadjusted string (held at constant length) was to go sharp or flat with changes in temperature. For nylon and fluorocarbon strings, the thermal variation in the string tension will be determined by changes in its stiffness and its unstretched length, with these effects being governed in turn by changes in the Young’s modulus and the coefficient of linear thermal expansion (CLTE). On this basis, a simple analysis [1] provided a means of estimating the CLTE from the observed thermal sensitivities in the string tension and Young’s modulus.

**Figure 11:**
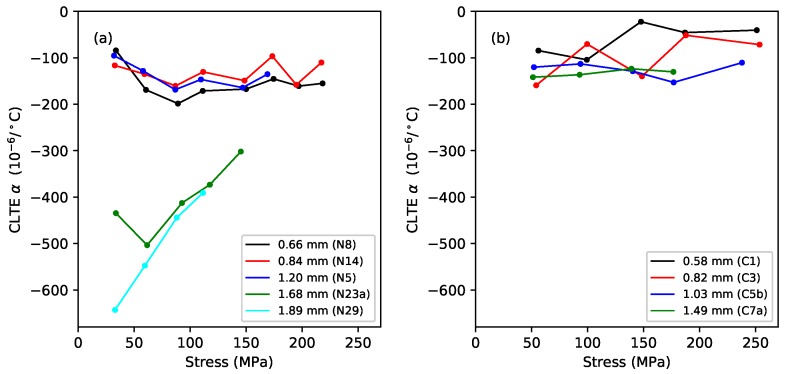
The coefficient of linear thermal expansion (CLTE) along the strings, plotted against the applied stress, for (**a**) nylon, and (**b**) fluorocarbon. The CLTE values were derived from the thermal tension sensitivity, in the absence of any tuning adjustments, and the thermal variation in the Young’s modulus.

Figure 11 shows the CLTE values obtained in this way for the nylon and fluorocarbon strings, plotted against the applied stress. In every case the CLTE was negative, indicating that the strings were trying to contract longitudinally as they were heated. This is an entropic effect [20], which would tend to increase the tension and make the strings go sharp. The CLTE values for the fluorocarbon strings were fairly similar to those obtained from the lower-density group of nylon strings, with no particular distinction between the wound and monofilament strings.

For gut strings, a third tension variation effect needs to be taken into account: as the humidity increases, the string fibres absorb moisture (increasing the linear density) and expand radially. It is believed that this expansion, within the twisted structure of the string, can in turn cause an increase in the string tension [21]. This effect could be expected to vary with the twist ratio and could provide a plausible explanation for the apparent coupling between changes in the string linear density and tension. This effect does not, however, appear to have been tested directly, and it is conceivable that some other mechanism may cause the longitudinal tension to change as the gut fibres absorb and release moisture.

If it could be assumed that the longitudinal CLTE and variations in the string’s Young’s modulus were not affected by changes in humidity, then it might be possible to estimate the CLTE by introducing an additional term, proportional to ψ, into the expression given for λ in the previous study of nylon strings [1]. Following such a route suggests that the longitudinal CLTE of the gut strings tested here was generally negative, as for the nylon and fluorocarbon strings, and with an approximately similar magnitude. This should properly be regarded as speculation, however, since the data were not clear enough to provide conclusive support for such an assumption.

For the nylon and fluorocarbon strings, the overall tension response to an increase in temperature will depend on the balance between the reduction in the Young’s modulus (Figure 4) and the contraction from the CLTE. These effects work against each other and tend to be fairly evenly matched [1]. For the nylon strings, the thermal contraction was usually the dominant effect, causing the strings to tighten and go sharp as the temperature was increased; while for the fluorocarbon strings, the balance varied from string to string.

For the gut strings, in nearly every case tested, the tension of the strings held at constant length fell as the temperature rose; the opposite behaviour to that seen for the nylon strings. The thermal tension sensitivities of the two materials were of about the same magnitude, but with opposite signs. If the speculation regarding the negative sign of the longitudinal CLTE for the gut strings is correct, then the reduction in the Young’s modulus must have been the more significant effect, causing the strings to relax and go flat.

The λ response plots in the top row of Figure 10 illustrate rather nicely how the balance between the various thermal tension effects varies across the different string materials. However, it should be noted (for all three materials) that if the temperature were to be increased too far, the string would be likely to start creeping again, causing it to go flat.

## 4. Additional Results for Gut Strings

**Figure 12:**
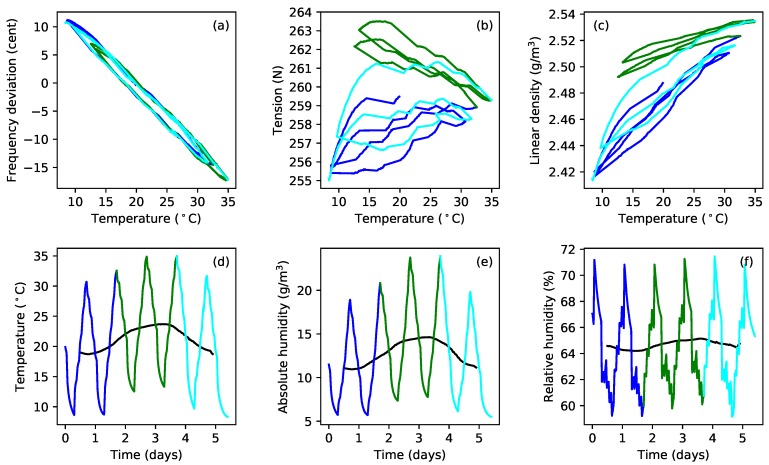
(**a**) The frequency deviation, (**b**) tension, and (**c**) linear density responses to temperature changes for the constant-length test with gut string G5c at a target fundamental frequency of 324 Hz. The variation in the tension and linear density response gradients appear more likely to correspond to a change in (**d**) the overall temperature or (**e**) the absolute humidity level, than to a change in (**f**) the relative humidity. The black lines show the average levels over a rolling 24-h period. The tuning sensitivity seems remarkably unaffected.

Figure 12 shows an interesting twist to the thermal tension sensitivity behaviour for gut. The first three plots (Figure 12a–c) show the frequency deviation, tension, and linear density responses to changes in temperature for the constant-length test with string G5c, at a target fundamental frequency of 324 Hz. The gradient of the tension response swings quite dramatically from a positive to a negative value, and then back again, during the course of the test; with a similarly distinct variation in the linear density response. The tuning response, however, maintains a fairly consistent gradient throughout and seems remarkably unaffected. Examination of the corresponding temperature and humidity results (Figure 12d–f) reveals that the variations in the tension and linear density response gradients seem to correlate with variation in the overall temperature or absolute humidity level, rather than with variation in the relative humidity. The black lines show the average levels over a rolling 24-h period.

Changes of this magnitude have implications for the derived values for the string bulk density. For the responses shown in Figure 5 and Figure 10, the values for the thermal variations in the string tension dF/dT and linear density dμ/dT have been taken from the green portions of the responses shown in Figure 12b,c, since the average temperature level over that period was more consistent with the other measurements made for that string. The dotted green forks in Figure 5c and Figure 10c,f show how those responses are affected if the values for dF/dT and dμ/dT are taken instead from the earlier blue sections of the responses in Figure 12b,c.

**Figure 13:**
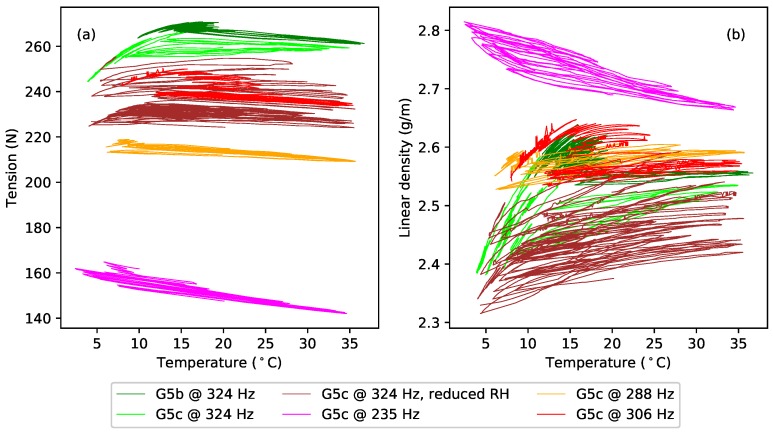
(**a**) Tension and (**b**) linear density versus temperature responses for strings G5b and G5c during the extended tests.

An examination of the responses obtained for strings G5b and G5c during the periods when they were left to follow the ambient changes in temperature and humidity, without any applied heating, showed that the behaviour seen in Figure 12 occurred to an even more pronounced extent as the temperature fell. Figure 13 shows the extended tension and linear density versus temperature responses for these string sections (green for G5b, lime for G5c), both during the period when heating was being applied, and during the longer subsequent period without heating. For both string sections, it can be seen that the dF/dT gradient was negative when the overall temperature levels were relatively high (during the applied heating cycles), but changed quite dramatically, becoming increasingly positive, as the overall temperature level fell. A similar range of variation can be seen in the dμ/dT gradients, though these did not generally appear to go negative. Looking back at the tuning deviation responses in Figure 8a, it seems even more remarkable that the tuning sensitivity gradient should remain so unchanging given the rather dramatic variations in the tension and linear density responses in Figure 13. This seems a further clear indication that λ and ψ must be coupled in some way.

Additional tests were run with string G5c. Following the initial extended test at a target frequency of 324 Hz, the water reservoir used to maintain the humidity levels [1] was removed from the test rig, and the heating cycle was restarted, to dry out the test chamber somewhat. This achieved a reduction in the relative humidity levels of approximately 10–15% RH. Once the humidity levels had fallen, the heating cycle was varied to reduce the overall temperature level while still keeping the chamber relatively dry. These tests resulted in the brown responses shown in Figure 13. The water reservoir was then replaced and the string tuned back down to, and tested at, a target frequency of 235 Hz (magenta responses), followed by tests at target frequencies of 288 Hz (orange responses), and 306 Hz (red responses). At each target frequency, the test rig was first run in its constant-frequency mode, with heating applied, until the string appeared to have stopped creeping. A test was then run with the string held at constant length, while the heating cycle was varied to change the overall (average) temperature level.

In each case, the same consistency in the thermal tuning sensitivity shown in Figure 8a was observed. With the exception of the test at 235 Hz (magenta), the tension and linear density responses all showed similar changes in their gradients as the overall temperature levels changed. For the test at 235 Hz, the tension and linear density responses both showed consistently negative gradients. The difference was particularly striking in the linear density response, as if some threshold had been crossed.

**Figure 14:**
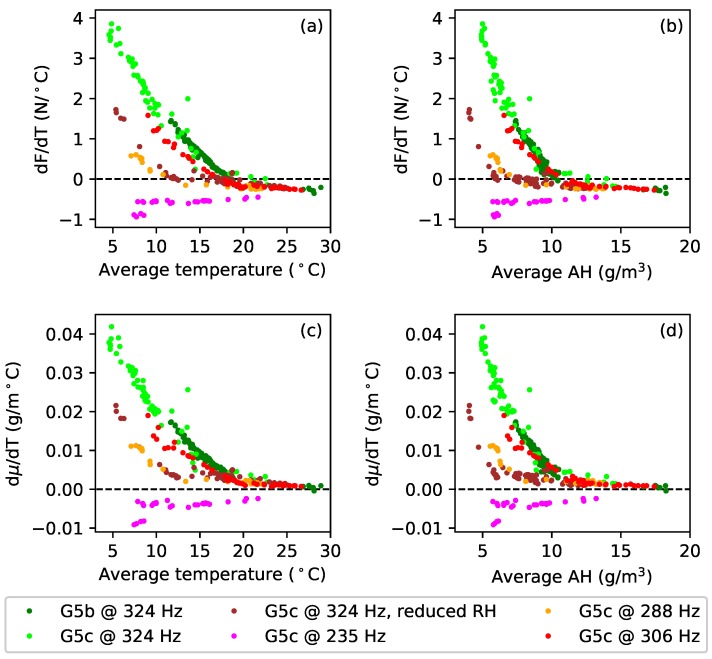
(**a**,**b**) The tension thermal sensitivity dF/dT values, and (**c**,**d**) the linear density thermal sensitivity dμ/dT values, for each temperature cycle during the extended tests, plotted against the average temperature and absolute humidity during the same periods.

To explore this behaviour in more detail, the dF/dT and dμ/dT gradients were calculated, between successive temperature minima and successive temperature maxima, for each temperature cycle in the extended test responses. Since many of the tension and linear density response cycles showed a clear curvature (Figure 13), quadratic functions were fitted to each cycle, and the dF/dT and dμ/dT gradients were calculated at the average temperature for that cycle. Figure 14 shows the dF/dT and dμ/dT values obtained in this way, plotted against the average temperature values (Figure 14a,c) and also against the corresponding average absolute humidity values (Figure 14b,d).

All four of these plots show a clear trend: the dF/dT and dμ/dT sensitivities increased progressively as the average temperature and absolute humidity fell, and with good consistency between the results for the two sections of the G5 string (green and lime). The results at 235 Hz were again the exception, with the dF/dT and dμ/dT values instead falling with the average temperature and absolute humidity. At all the higher test frequencies, all four responses also show a fairly sharp ‘elbow’; again possibly suggesting some sort of threshold effect. Using the average relative humidity as the abscissa did not show any such trend in the dF/dT or dμ/dT behaviour, confirming that this effect seems indeed to be a function of either temperature or absolute humidity. An implication is that many of the gut string results presented in this paper may only be applicable for average temperature levels in the range of about 20–30 °C, or absolute humidity levels in the range of about 10–20 g/m${}^{3}$. Below those ranges, something seems to change in the physical behaviour.

The brown (324 Hz, reduced RH) and orange (288 Hz) dF/dT and dμ/dT responses in Figure 14 all have their elbows at about the same points. Compared to the brown responses, the elbows in the orange responses for dF/dT and dμ/dT occurred at about the same or slightly lower average temperature levels, but at slightly higher average absolute humidity levels. The elbows in the red (306 Hz) responses showed a similar pattern of behaviour compared to the green and lime (324 Hz) responses. Looking at the corresponding tension and linear density responses in Figure 13, it is tempting to suggest that the position of the elbow may be tension or stress related. A similar test was run with the fluorocarbon string C5b, at its last target frequency (375 Hz), but showed virtually no variation in the levels of dF/dT and dμ/dT as the average temperature and absolute humidity levels were varied. Whatever this behaviour is, it appears to be specific to the gut strings.

### Results for Humidity Dependence

Ambient humidity levels tend to vary both with and relative to the temperature. As the temperature increases, the absolute humidity will usually increase with it, while the relative humidity falls. At other times, however, the humidity may change independently of the temperature, with both the absolute and relative humidity levels changing in the same direction. Both of these behaviours are illustrated in Figure 8b,c.

Within the test rig, a water bottle with its top cut off was used to provide a reservoir to maintain the humidity levels; without this, the test chamber would have dried out excessively. It was found that the humidity levels could be changed by varying the exposed surface area of the water, and also by varying the level of the water within the bottle. To keep the humidity levels fairly constant, the water level was topped up regularly. After a while, an automated water top-up system was added, which maintained the water level to within about 1 cm. These measures did not, however, isolate the test chamber (which was not completely sealed, to allow for expansion and contraction of the air within it) from changes in the external ambient humidity. It was found that even quite modest changes in the weather could have quite dramatic effects on the behaviour of gut strings, as seen above in Figure 12.

The preceding analysis has largely examined the behaviour with changes in humidity accompanying changes in temperature. In an attempt to examine the behaviour due to independent changes in humidity, lines were fitted to the tension and linear density versus temperature responses during the initial part of the extended tests, described above, when the full heating cycle was being applied. These fitted expressions were then used to estimate the parameter variations, which might be expected purely from the changes in temperature during the full duration of the extended tests, when various measures were taken to vary the overall temperature and humidity levels. The differences between the actual parameter responses and the expected responses due to the temperature variations were then examined. Given the variations that have been observed in dF/dT and dμ/dT with the average temperature level (Figure 13 and Figure 14), this approach will not fully remove the effects of temperature. It should, though, largely remove the effects on the string tension due to longitudinal thermal expansion and changes in the Young’s modulus, assuming these effects, both of which may be due primarily to intra-molecular effects, are relatively unaffected by the moisture content of the string.

**Figure 15:**
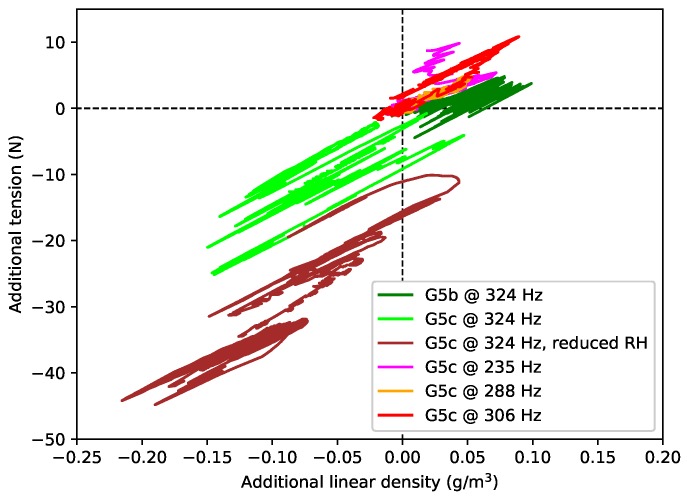
The additional (non-temperature related) string tension versus the additional linear density responses obtained during the extended tests with no tuning adjustments.

Figure 15 shows the estimated additional tension (the difference between the actual and expected values) plotted against the additional linear density. Each response starts from the origin. The gradients of the responses are generally linear and consistent, which matches the supposition that changes in humidity generally affect both the linear density and tension to an approximately equal extent. There are, however, additional and irregular shifts in the string tension. Noting that the string had been tuned up before the start of the tests at 324 Hz (green, lime, brown responses), during which the tension subsequently fell, and back down at the start of the test at 235 Hz (magenta), during which the tension rose, the behaviour rather suggests that the string had started creeping again.

## 5. Discussion and Conclusions

In this study, the long-term (post-creep) mechanical behaviour of fluorocarbon and natural gut harp strings has been examined, and the results compared with those from a previous study of rectified nylon harp strings [1]. The behaviour of the fluorocarbon strings was found to be similar in many ways to that of the nylon strings, despite the fluorocarbon strings having a different chemical formulation with significantly higher density.

The gut strings showed some similarities of behaviour to the synthetic materials, but also some major differences. Some of these differences probably stem from chemistry, while others are perhaps due to the wound structure of the gut strings studied, with no central core. The regulated biological growth processes for natural gut, compared to the more random molecular arrangement of synthetic strings formed from an initial melt, is also likely to be important.

A big difference between natural gut and the synthetic materials examined is the relatively high sensitivity of gut to changes in humidity. The consequences of this sensitivity were observed very clearly, and yet, some of the results, especially the thermal tuning sensitivity of the strings when held at constant length, displayed remarkable and quite unexpected regularities. The observed behaviour suggests very strongly that there is a direct coupling between humidity-related changes in the linear density of a gut string and complementary changes in its tension.

### 5.1. Stretching Behaviour

The stretching behaviour of the three string materials was studied via three different measures of the Young’s modulus, with very different test time scales: on the order of weeks, minutes, and milliseconds. In each case, and as expected, the Young’s modulus increased with the effective frequency of the test method (Figure 2).

For both the fluorocarbon and nylon strings, the faster measures of Young’s modulus $E_{B}$ and $E_{T}$ showed very similar responses; increasing linearly with the applied stress through much the same range of values and with more or less the same gradient. These measures of Young’s modulus are largely unaffected by the slow creep response of the strings, the relevant test cycles being too short for such creep to make a significant contribution. The similarity of the response ranges and gradients suggests that both were governed by the same mechanism, which seems likely to involve the progressive straightening of long-chain polymer molecules. The relatively constant levels of the slower measure of Young’s modulus $E_{S}$, in which the creep was specifically included, indicates that the progressive stiffening of the material as the applied stress and strain were increased was being offset by a progressive increase in creep deformation.

For the slow $E_{S}$ and intermediate $E_{T}$ measures of Young’s modulus, there was no particular distinction between the behaviour of monofilament and over-wrapped fluorocarbon strings. Presumably, the presence of the core in the over-wrapped strings governed their behaviour in these cases. For the fast measure $E_{B}$, which was derived from the string bending stiffness, the effective Young’s modulus of the over-wrapped strings was lower than that of the monofilament strings. This was to be expected, since a key motivation for using an over-wrapped string construction is to increase the linear density while keeping down the bending stiffness, and the associated inharmonicity in the string overtones.

The stretching behaviour of the gut strings was very different from that of the two synthetic materials. All three measures of Young’s modulus remained essentially constant as the stress and strain were increased, right up to the point where the strings broke: it appears that these gut strings can reasonably be modelled as a linear viscoelastic material over the entire operational range of stress and strain. A further consequence is that the bending stiffness of gut strings will generally be lower than that of the synthetic strings, with lower inharmonicity in the string overtones. A comparison with the Young’s modulus results reported in a previous study of gut strings [7] showed that the earlier results were consistent with those shown here, given the different methods by which the values were obtained.

All three materials maintained almost constant bulk density, after their initial stretching, as the strain was increased; with a progressive reduction in the linear density associated primarily with the string thinning as it was stretched (Figure 5). The initial density reduction appeared to proceed more quickly with the gut strings, but in some cases, stopped at a lower strain level than with the nylon and fluorocarbon strings.

### 5.2. Responses to Temperature and Humidity Changes

For all three string materials, the thermal tuning sensitivity with the strings held at constant length (with no tuning adjustments) showed a high degree of correlation with the inverse of the applied stress.

For rectified nylon strings, it was shown in the earlier study that thermally-induced changes in the string linear density can be largely ignored [1]. The thermal tuning behaviour of the string is determined primarily by the change in its tension λ, which in turn is determined by its longitudinal CLTE and the thermal variation in its Young’s modulus dE/dT. These effects have opposite sense: the Young’s modulus falls as the temperature rises, tending to reduce the string tension, while the CLTE is negative, working to contract the string and increase its tension. They are also of approximately equal magnitude, so the balance between them is quite sensitive. This is demonstrated by the difference in the behaviour of the two groups of nylon strings: for the thinner, lower-density strings, the net effect was for the tension to increase as the temperature rose, making the string go sharp. For the thicker, higher-density strings, the situation was more complicated: the strings usually went sharp as the temperature rose, except at low stress levels, where they went flat (Figure 7a).

The fluorocarbon strings tested here showed much lower thermal tuning sensitivities than the nylon strings (Figure 7). The distinction now was between the monofilament strings, which always went sharp as the temperature rose, and the over-wrapped strings, which generally went sharp except at low stress levels. The CLTE and dE/dT values had the same signs and appeared to have slightly smaller magnitudes compared to nylon (Figure 4 and Figure 11), but more importantly, the balance between them was closer, resulting in smaller values for the thermal tension sensitivity λ (Figure 10). In fact, the values of λ for the fluorocarbon strings appeared to be of comparable (small) size to the values for the thermal variation in the linear density ψ. A consequence was that holding the fluorocarbon strings at constant tension yielded only a small improvement in the overall thermal tuning sensitivity, whereas for the nylon strings, the improvement was more dramatic.

The thermal behaviour of the gut strings was again very different in some ways, compared to the two synthetic materials. In particular, the unadjusted gut strings generally went flat as the temperature was increased (Figure 7c). The sensitivity of the gut strings to changes in humidity resulted in much larger, and more erratic, thermal variations in their linear density ψ (Figure 10f). One consequence of this was that holding the gut strings at constant tension did not generally improve their tuning performance (Figure 9c). The thermal tension behaviour for gut strings is also more complicated than for the synthetic strings, due to the need to take account of the possibility that changes in the diameter of the gut fibres, as they absorb and release moisture, can also affect the string tension, possibly as a result of the twisted structure of the fibre bundle making up the string.

Extended tests with one of the gut strings, with the string held at constant length while the temperature and humidity levels were varied, demonstrated that the thermal tuning sensitivity in fact remained remarkably constant (Figure 8), even with significant changes in humidity and over long periods (the tests on the two sections of the G5 string were made two years apart). This behaviour suggests very strongly that the changes in the string linear density are largely cancelled out by complementary changes in the string tension, presumably through some coupling mechanism such as the twisted structure of the fibre bundle. It would appear, therefore, that the overall thermal tuning behaviour of gut strings may also depend primarily on the balance between the CLTE and dE/dT. As for the synthetic materials, the Young’s modulus of the gut strings generally fell as the temperature increased. The longitudinal CLTE could not be reliably determined for the gut strings, but was suspected to be generally negative, again as for the synthetic strings.

A more detailed examination of the thermal variations in the string tension dF/dT and linear density dμ/dT, during the extended tests with the G5 gut string, revealed the apparent presence of thresholds, with different regions of behaviour for dF/dT and dμ/dT (Figure 14). The cause and meaning of these is unknown, but the invariance in the overall thermal tuning sensitivity, and hence the apparent coupling between ψ and λ, was observed consistently across the different regions.

Finally, an attempt was made to decouple the effects of different types of humidity changes: those that accompany changes in temperature, and those that occur independently of the temperature (Figure 15). The clear linking between ψ and λ was again observed, coupled with additional irregular shifts in the string tension. It appeared that the latter may correspond to additional periods of string creep, though this observation is somewhat speculative at this stage. It is conceivable, though, that changes in humidity could alter the inter-molecular spacing within the gut fibres, weakening cross-links and allowing further creep to commence.

The results for gut strings raise a question about the role of the string’s twist ratio in the apparent coupling between changes in the string linear density and complementary changes in its tension. A target for future work would be to investigate how gut string behaviour is affected by deliberate changes in the twist ratio.

The original motivation behind this study was to investigate whether the behaviour of the different string materials could be understood sufficiently to develop pre-emptive tuning compensators to maintain the string tuning during a performance. As previously identified for rectified nylon strings [1], a practical approach for fluorocarbon strings may be to hold them at constant tension. It is debatable, though, whether the improvement that might be obtained is really worth the cost and effort involved. For gut strings, things are more difficult. It might be possible for an adaptive algorithm to learn the tuning sensitivity gradient for the regular, and apparently quite stable, thermal changes in string behaviour. However, it is hard to see how the additional and apparently irregular changes associated with humidity variations could be accounted for. Overall it would seem that the best approach, certainly for gut strings, would be to devise a means of measuring the string fundamental frequency more directly, but without the need for the string to be plucked or played, such as that proposed in [19].

## Figures and Tables

**Table 1:** The set of strings studied, showing the unstretched string parameters, and the target fundamental frequencies used for testing. The number in the first column provides a unique reference for each test string or string section. It can be used to cross-reference with the summary datasets submitted with this paper and the larger datasets available at https://doi.org/10.17863/CAM.9018 and https://doi.org/10.17863/CAM.31653 (Supplementary Material).

No.	Note	Diameter (mm)	Density (kg/m${}^{3}$)	Target Test Frequencies (Hz)	Comments or Test Period
				NYLON
N8	A6 (5)	0.658	1097	174, 235, 287, 323, 375, 403, 429 *, 453	Lower density group
N14	A5 (12)	0.841	1072	174, 235, 287, 323, 375 *, 403, 429, 453	Lower density group
N5	A4 (19)	1.196	1076	174, 235, 287, 323 *, 375, 403	Lower density group
N23a	A3 (26)	1.680	1151	174, 235, 287 *, 323, 360	Higher density group
N29	E3 (29)	1.894	1159	174, 235, 287, 323	Higher density group
				FLUOROCARBON
C1	A6 (5)	0.578	1819	174, 235, 287, 323, 375	Monofilament
C3	A5 (12)	0.819	1812	174, 235, 287, 323, 375 *	Monofilament
C5b	A4 (19)	1.031	1736	174, 235, 287, 323 *, 375	Wound construction
C7a	A3 (26)	1.495	1729	174, 235, 287 *, 323	Wound construction
				GUT
G2b	A5 (12)	0.844	1320	174, 235, 288, 324, 376 *, 404	August–December 2014
G3b	A4 (19)	1.167	1325	174, 235, 288, 324 *, 376, 404, 431	December 2014–May 2015
G3c	A4 (19)	1.167	1325	174, 235, 288, 324 *, 376, 404	May–August 2017
G5b	A3 (26)	1.629	1320	174, 235, 288 *, 324	May–November 2015
G5c	A3 (26)	1.629	1320	174, 235, 288 *, 306 ${}^{†}$, 324	September 2017–April 2018

* The test frequencies marked with an asterisk correspond to the expected operating points on the harp. These points are included in some of the figures as black circles. ${}^{†}$ The 306-Hz test frequency for G5c was used only for the extended gut tests exploring the effects of changes in the overall temperature and humidity levels.

## Supplementary Materials

The following are available online at http://www.mdpi.com/1996-1944/11/11/2160/s1: summary and measurement data files, plus a file guide and the Python code for generating the plots. Much larger comprehensive datasets are available at https://doi.org/10.17863/CAM.9018 (nylon) and https://doi.org/10.17863/CAM.31653 (fluorocarbon and gut).

## Abbreviations

The following abbreviations are used in this manuscript:

AH	Absolute humidity
CLTE	Coefficient of linear thermal expansion
DMA	Dynamic mechanical analyser
RH	Relative humidity
PVDF	Polyvinylidene fluoride
