# Supplementary material for: Comparison of Mechanical Properties of Natural Gut and Synthetic Polymer Harp Strings
Source: Materials (Basel). 2018 Nov 1;11(11):2160. doi: 10.3390/ma11112160 (PMC6266393; doi:10.3390/ma11112160)
Supplement: Supplementary file 1 [file materials-11-02160-s001.zip › Figures+for+comparison+paper.html]

Figures for comparison paper


# Figures for Comparison of Mechanical Properties of Natural Gut and Synthetic Polymer Harp Strings¶

In [1]:

```
import xlrd
import numpy as np
#import matplotlib as mlt
#mlt.use('nbagg') # makes plots interactive for zooming. Need to restart notebook to change this setting.
import matplotlib.pyplot as plt
import matplotlib.ticker as ticker
from mpl_toolkits.mplot3d import Axes3D
# set latex expressions to same font as other text
#mlt.rcParams['mathtext.fontset'] = 'custom'
#mlt.rcParams['mathtext.rm'] = 'Bitstream Vera Sans'
#mlt.rcParams['mathtext.it'] = 'Bitstream Vera Sans:italic'
#mlt.rcParams['mathtext.bf'] = 'Bitstream Vera Sans:bold'
#mlt.rcParams['mathtext.cal'] = 'Bitstream Vera Sans:cursive'
# enable plots to be shown in cells
%matplotlib inline

def r_squared(y_act, y_est):
    y_mean = np.mean(y_act)
    sumsq_tot = np.sum((y_act - y_mean)**2)
    sumsq_res = np.sum((y_act - y_est)**2)
    return (1.0 - sumsq_res/sumsq_tot)

op_pen = 'black'
op_marker = 'o' # 'x'
op_ms = 6
op_mfc = 'none'
op_mew = 2
op_leg = 'operating points'
```

## Read data for rectified nylon¶

In [2]:

```
# READ DATA FOR RECTIFIED NYLON
# connect to data source
file_location = "./NLTP master data file.xls"
workbook1 = xlrd.open_workbook(file_location)
data_sheet = workbook1.sheet_by_index(0)
#
# List sheet names
sheet_names = workbook1.sheet_names()
print('Sheet Names 1:', sheet_names)
#
# define data arrays
# Note: use zero-indexing for row and column numbers
# row spans
n8_start_row = 15
n8_stop_row = 23
n14_start_row = 32
n14_stop_row = 40
n5_start_row = 49
n5_stop_row = 55
n23a_start_row = 64
n23a_stop_row = 69
n29_start_row = 78
n29_stop_row = 82
n10b_start_row = 91
n10b_stop_row = 93
nop_start_row = 105
nop_stop_row = 114
nop_range = [106,107,110,111]
# columns
init_d_col = 13
rho_n_col = 16
Fn_col = 17
Fw_col = 26
F0_col = 51
stress_n_col = 18
stress_col = 27
strain_col = 21
Et_col = 37
Eb_col = 44
Es_col = 34
dfdT_L_col = 54
dfdT_F_col = 57
dFdT_col = 50
dEtdT_col = 38
dEbdT_col = 43
CLTE_col = 46
psi_col = 53
den_ratio_col = 69
den_err_plus_col = 70
den_err_minus_col = 71
#
# n8 - A6
n8_dia_n = data_sheet.cell_value(n8_start_row, init_d_col)
n8_rho_n = [data_sheet.cell_value(n8_start_row, rho_n_col) for i in range(n8_start_row, n8_stop_row)]
n8_Fn = [data_sheet.cell_value(i, Fn_col) for i in range(n8_start_row, n8_stop_row)]
n8_Fw = [data_sheet.cell_value(i, Fw_col) for i in range(n8_start_row, n8_stop_row)]
n8_F0 = [data_sheet.cell_value(i, F0_col) for i in range(n8_start_row, n8_stop_row)]
n8_stress_n = [data_sheet.cell_value(i, stress_n_col) for i in range(n8_start_row, n8_stop_row)]
n8_stress = [data_sheet.cell_value(i, stress_col) for i in range(n8_start_row, n8_stop_row)]
n8_strain = [data_sheet.cell_value(i, strain_col) for i in range(n8_start_row, n8_stop_row)]
n8_Et = [data_sheet.cell_value(i, Et_col) for i in range(n8_start_row, n8_stop_row)]
n8_Eb = [data_sheet.cell_value(i, Eb_col) for i in range(n8_start_row, n8_stop_row)]
n8_Es = [data_sheet.cell_value(i, Es_col) for i in range(n8_start_row, n8_stop_row)]
n8_dfdT_L = [data_sheet.cell_value(i, dfdT_L_col) for i in range(n8_start_row, n8_stop_row)]
n8_dfdT_F = [data_sheet.cell_value(i, dfdT_F_col) for i in range(n8_start_row, n8_stop_row)]
n8_dFdT = [data_sheet.cell_value(i, dFdT_col) for i in range(n8_start_row, n8_stop_row)]
n8_dEtdT = [data_sheet.cell_value(i, dEtdT_col) for i in range(n8_start_row, n8_stop_row)]
n8_CLTE = [(data_sheet.cell_value(i, CLTE_col)*1e6) for i in range(n8_start_row, n8_stop_row)]
n8_psi = [(data_sheet.cell_value(i, psi_col)*1000) for i in range(n8_start_row, n8_stop_row)]
#
n8_stress0 = [data_sheet.cell_value(i, stress_col) for i in range(n8_start_row-1, n8_stop_row)]
n8_strain0 = [data_sheet.cell_value(i, strain_col) for i in range(n8_start_row-1, n8_stop_row)]
n8_den_ratio = [data_sheet.cell_value(i, den_ratio_col) for i in range(n8_start_row-1, n8_stop_row)]
n8_den_err_plus = [data_sheet.cell_value(i, den_err_plus_col) for i in range(n8_start_row-1, n8_stop_row)]
n8_den_err_minus = [data_sheet.cell_value(i, den_err_minus_col) for i in range(n8_start_row-1, n8_stop_row)]
#
# n14 - A5
n14_dia_n = data_sheet.cell_value(n14_start_row, init_d_col)
n14_rho_n = [data_sheet.cell_value(n14_start_row, rho_n_col) for i in range(n14_start_row, n14_stop_row)]
n14_Fn = [data_sheet.cell_value(i, Fn_col) for i in range(n14_start_row, n14_stop_row)]
n14_Fw = [data_sheet.cell_value(i, Fw_col) for i in range(n14_start_row, n14_stop_row)]
n14_F0 = [data_sheet.cell_value(i, F0_col) for i in range(n14_start_row, n14_stop_row)]
n14_stress_n = [data_sheet.cell_value(i, stress_n_col) for i in range(n14_start_row, n14_stop_row)]
n14_stress = [data_sheet.cell_value(i, stress_col) for i in range(n14_start_row, n14_stop_row)]
n14_strain = [data_sheet.cell_value(i, strain_col) for i in range(n14_start_row, n14_stop_row)]
n14_Et = [data_sheet.cell_value(i, Et_col) for i in range(n14_start_row, n14_stop_row)]
n14_Eb = [data_sheet.cell_value(i, Eb_col) for i in range(n14_start_row, n14_stop_row)]
n14_Es = [data_sheet.cell_value(i, Es_col) for i in range(n14_start_row, n14_stop_row)]
n14_dfdT_L = [data_sheet.cell_value(i, dfdT_L_col) for i in range(n14_start_row, n14_stop_row)]
n14_dfdT_F = [data_sheet.cell_value(i, dfdT_F_col) for i in range(n14_start_row, n14_stop_row)]
n14_dFdT = [data_sheet.cell_value(i, dFdT_col) for i in range(n14_start_row, n14_stop_row)]
n14_dEtdT = [data_sheet.cell_value(i, dEtdT_col) for i in range(n14_start_row, n14_stop_row)]
n14_dEbdT = [data_sheet.cell_value(i, dEbdT_col) for i in range(n14_start_row, n14_stop_row)]
n14_CLTE = [(data_sheet.cell_value(i, CLTE_col)*1e6) for i in range(n14_start_row, n14_stop_row)]
n14_psi = [(data_sheet.cell_value(i, psi_col)*1000) for i in range(n14_start_row, n14_stop_row)]
#
n14_stress0 = [data_sheet.cell_value(i, stress_col) for i in range(n14_start_row-1, n14_stop_row)]
n14_strain0 = [data_sheet.cell_value(i, strain_col) for i in range(n14_start_row-1, n14_stop_row)]
n14_den_ratio = [data_sheet.cell_value(i, den_ratio_col) for i in range(n14_start_row-1, n14_stop_row)]
n14_den_err_plus = [data_sheet.cell_value(i, den_err_plus_col) for i in range(n14_start_row-1, n14_stop_row)]
n14_den_err_minus = [data_sheet.cell_value(i, den_err_minus_col) for i in range(n14_start_row-1, n14_stop_row)]
#
# n5 - A4
n5_dia_n = data_sheet.cell_value(n5_start_row, init_d_col)
n5_rho_n = [data_sheet.cell_value(n5_start_row, rho_n_col) for i in range(n5_start_row, n5_stop_row)]
n5_Fn = [data_sheet.cell_value(i, Fn_col) for i in range(n5_start_row, n5_stop_row)]
n5_Fw = [data_sheet.cell_value(i, Fw_col) for i in range(n5_start_row, n5_stop_row)]
n5_F0 = [data_sheet.cell_value(i, F0_col) for i in range(n5_start_row, n5_stop_row)]
n5_stress_n = [data_sheet.cell_value(i, stress_n_col) for i in range(n5_start_row, n5_stop_row)]
n5_stress = [data_sheet.cell_value(i, stress_col) for i in range(n5_start_row, n5_stop_row)]
n5_strain = [data_sheet.cell_value(i, strain_col) for i in range(n5_start_row, n5_stop_row)]
n5_Et = [data_sheet.cell_value(i, Et_col) for i in range(n5_start_row, n5_stop_row)]
n5_Eb = [data_sheet.cell_value(i, Eb_col) for i in range(n5_start_row, n5_stop_row)]
n5_Es = [data_sheet.cell_value(i, Es_col) for i in range(n5_start_row, n5_stop_row)]
n5_dfdT_L = [data_sheet.cell_value(i, dfdT_L_col) for i in range(n5_start_row, n5_stop_row)]
n5_dfdT_F = [data_sheet.cell_value(i, dfdT_F_col) for i in range(n5_start_row, n5_stop_row)]
n5_dFdT = [data_sheet.cell_value(i, dFdT_col) for i in range(n5_start_row, n5_stop_row)]
n5_dEtdT = [data_sheet.cell_value(i, dEtdT_col) for i in range(n5_start_row, n5_stop_row)]
n5_dEbdT = [data_sheet.cell_value(i, dEbdT_col) for i in range(n5_start_row, n5_stop_row)]
n5_CLTE = [(data_sheet.cell_value(i, CLTE_col)*1e6) for i in range(n5_start_row, n5_stop_row)]
n5_psi = [(data_sheet.cell_value(i, psi_col)*1000) for i in range(n5_start_row, n5_stop_row)]
#
n5_stress0 = [data_sheet.cell_value(i, stress_col) for i in range(n5_start_row-1, n5_stop_row)]
n5_strain0 = [data_sheet.cell_value(i, strain_col) for i in range(n5_start_row-1, n5_stop_row)]
n5_den_ratio = [data_sheet.cell_value(i, den_ratio_col) for i in range(n5_start_row-1, n5_stop_row)]
n5_den_err_plus = [data_sheet.cell_value(i, den_err_plus_col) for i in range(n5_start_row-1, n5_stop_row)]
n5_den_err_minus = [data_sheet.cell_value(i, den_err_minus_col) for i in range(n5_start_row-1, n5_stop_row)]
#
# n23a - A3
n23a_dia_n = data_sheet.cell_value(n23a_start_row, init_d_col)
n23a_rho_n = [data_sheet.cell_value(n23a_start_row, rho_n_col) for i in range(n23a_start_row, n23a_stop_row)]
n23a_Fn = [data_sheet.cell_value(i, Fn_col) for i in range(n23a_start_row, n23a_stop_row)]
n23a_Fw = [data_sheet.cell_value(i, Fw_col) for i in range(n23a_start_row, n23a_stop_row)]
n23a_F0 = [data_sheet.cell_value(i, F0_col) for i in range(n23a_start_row, n23a_stop_row)]
n23a_stress_n = [data_sheet.cell_value(i, stress_n_col) for i in range(n23a_start_row, n23a_stop_row)]
n23a_stress = [data_sheet.cell_value(i, stress_col) for i in range(n23a_start_row, n23a_stop_row)]
n23a_strain = [data_sheet.cell_value(i, strain_col) for i in range(n23a_start_row, n23a_stop_row)]
n23a_Et = [data_sheet.cell_value(i, Et_col) for i in range(n23a_start_row, n23a_stop_row)]
n23a_Eb = [data_sheet.cell_value(i, Eb_col) for i in range(n23a_start_row, n23a_stop_row)]
n23a_Es = [data_sheet.cell_value(i, Es_col) for i in range(n23a_start_row, n23a_stop_row)]
n23a_dfdT_L = [data_sheet.cell_value(i, dfdT_L_col) for i in range(n23a_start_row, n23a_stop_row)]
n23a_dfdT_F = [data_sheet.cell_value(i, dfdT_F_col) for i in range(n23a_start_row, n23a_stop_row)]
n23a_dFdT = [data_sheet.cell_value(i, dFdT_col) for i in range(n23a_start_row, n23a_stop_row)]
n23a_dEtdT = [data_sheet.cell_value(i, dEtdT_col) for i in range(n23a_start_row, n23a_stop_row)]
n23a_CLTE = [(data_sheet.cell_value(i, CLTE_col)*1e6) for i in range(n23a_start_row, n23a_stop_row)]
n23a_psi = [(data_sheet.cell_value(i, psi_col)*1000) for i in range(n23a_start_row, n23a_stop_row)]
#
n23a_stress0 = [data_sheet.cell_value(i, stress_col) for i in range(n23a_start_row-1, n23a_stop_row)]
n23a_strain0 = [data_sheet.cell_value(i, strain_col) for i in range(n23a_start_row-1, n23a_stop_row)]
n23a_den_ratio = [data_sheet.cell_value(i, den_ratio_col) for i in range(n23a_start_row-1, n23a_stop_row)]
n23a_den_err_plus = [data_sheet.cell_value(i, den_err_plus_col) for i in range(n23a_start_row-1, n23a_stop_row)]
n23a_den_err_minus = [data_sheet.cell_value(i, den_err_minus_col) for i in range(n23a_start_row-1, n23a_stop_row)]
#
# n29 - E3
n29_dia_n = data_sheet.cell_value(n29_start_row, init_d_col)
n29_rho_n = [data_sheet.cell_value(n29_start_row, rho_n_col) for i in range(n29_start_row, n29_stop_row)]
n29_Fn = [data_sheet.cell_value(i, Fn_col) for i in range(n29_start_row, n29_stop_row)]
n29_Fw = [data_sheet.cell_value(i, Fw_col) for i in range(n29_start_row, n29_stop_row)]
n29_F0 = [data_sheet.cell_value(i, F0_col) for i in range(n29_start_row, n29_stop_row)]
n29_stress_n = [data_sheet.cell_value(i, stress_n_col) for i in range(n29_start_row, n29_stop_row)]
n29_stress = [data_sheet.cell_value(i, stress_col) for i in range(n29_start_row, n29_stop_row)]
n29_strain = [data_sheet.cell_value(i, strain_col) for i in range(n29_start_row, n29_stop_row)]
n29_Et = [data_sheet.cell_value(i, Et_col) for i in range(n29_start_row, n29_stop_row)]
n29_Eb = [data_sheet.cell_value(i, Eb_col) for i in range(n29_start_row, n29_stop_row)]
n29_Es = [data_sheet.cell_value(i, Es_col) for i in range(n29_start_row, n29_stop_row)]
n29_dfdT_L = [data_sheet.cell_value(i, dfdT_L_col) for i in range(n29_start_row, n29_stop_row)]
n29_dfdT_F = [data_sheet.cell_value(i, dfdT_F_col) for i in range(n29_start_row, n29_stop_row)]
n29_dFdT = [data_sheet.cell_value(i, dFdT_col) for i in range(n29_start_row, n29_stop_row)]
n29_dEtdT = [data_sheet.cell_value(i, dEtdT_col) for i in range(n29_start_row, n29_stop_row)]
n29_dEbdT = [data_sheet.cell_value(i, dEbdT_col) for i in range(n29_start_row, n29_stop_row)]
n29_CLTE = [(data_sheet.cell_value(i, CLTE_col)*1e6) for i in range(n29_start_row, n29_stop_row)]
n29_psi = [(data_sheet.cell_value(i, psi_col)*1000) for i in range(n29_start_row, n29_stop_row)]
#
n29_stress0 = [data_sheet.cell_value(i, stress_col) for i in range(n29_start_row-1, n29_stop_row)]
n29_strain0 = [data_sheet.cell_value(i, strain_col) for i in range(n29_start_row-1, n29_stop_row)]
n29_den_ratio = [data_sheet.cell_value(i, den_ratio_col) for i in range(n29_start_row-1, n29_stop_row)]
n29_den_err_plus = [data_sheet.cell_value(i, den_err_plus_col) for i in range(n29_start_row-1, n29_stop_row)]
n29_den_err_minus = [data_sheet.cell_value(i, den_err_minus_col) for i in range(n29_start_row-1, n29_stop_row)]
#
# n10b - A6
n10b_rho_n = [data_sheet.cell_value(n10b_start_row, rho_n_col) for i in range(n10b_start_row, n10b_stop_row)]
n10b_stress_n = [data_sheet.cell_value(i, stress_n_col) for i in range(n10b_start_row, n10b_stop_row)]
n10b_Et = [data_sheet.cell_value(i, Et_col) for i in range(n10b_start_row, n10b_stop_row)]
n10b_Eb = [data_sheet.cell_value(i, Eb_col) for i in range(n10b_start_row, n10b_stop_row)]
#
# operating points
nop_Fn_full = [data_sheet.cell_value(i, Fn_col) for i in range(nop_start_row, nop_stop_row)]
nop_Fw_full = [data_sheet.cell_value(i, Fw_col) for i in range(nop_start_row, nop_stop_row)]
nop_Fn = [data_sheet.cell_value(i, Fn_col) for i in nop_range]
nop_Fw = [data_sheet.cell_value(i, Fw_col) for i in nop_range]
nop_stress = [data_sheet.cell_value(i, stress_col) for i in nop_range]
nop_dfdT_L = [data_sheet.cell_value(i, dfdT_L_col) for i in nop_range]
nop_dfdT_F = [data_sheet.cell_value(i, dfdT_F_col) for i in nop_range]

# inverse stress values
n8_inv_stress = 1.0 / np.asarray(n8_stress)
n14_inv_stress = 1.0 / np.asarray(n14_stress)
n5_inv_stress = 1.0 / np.asarray(n5_stress)
n23a_inv_stress = 1.0 / np.asarray(n23a_stress)
n29_inv_stress = 1.0 / np.asarray(n29_stress)
nop_inv_stress = 1.0 / np.asarray(nop_stress)

# Es stress values
n8_Es_stress = []
for i in range (0, len(n8_Es), 1):
    n8_Es_stress.append((n8_stress0[i]+n8_stress0[i+1])/2.0)
n14_Es_stress = []
for i in range (0, len(n14_Es), 1):
    n14_Es_stress.append((n14_stress0[i]+n14_stress0[i+1])/2.0)
n5_Es_stress = []
for i in range (0, len(n5_Es), 1):
    n5_Es_stress.append((n5_stress0[i]+n5_stress0[i+1])/2.0)
n23a_Es_stress = []
for i in range (0, len(n23a_Es), 1):
    n23a_Es_stress.append((n23a_stress0[i]+n23a_stress0[i+1])/2.0)
n29_Es_stress = []
for i in range (0, len(n29_Es), 1):
    n29_Es_stress.append((n29_stress0[i]+n29_stress0[i+1])/2.0)

# (1/F).dF/dT
n8_dFdTbyF = 1000.0 * np.asarray(n8_dFdT) / np.asarray(n8_F0)
n14_dFdTbyF = 1000.0 * np.asarray(n14_dFdT) / np.asarray(n14_F0)
n5_dFdTbyF = 1000.0 * np.asarray(n5_dFdT) / np.asarray(n5_F0)
n23a_dFdTbyF = 1000.0 * np.asarray(n23a_dFdT) / np.asarray(n23a_F0)
n29_dFdTbyF = 1000.0 * np.asarray(n29_dFdT) / np.asarray(n29_F0)

# plot colours
n8_pen = 'black'
n14_pen = 'red'
n5_pen = 'blue'
n23a_pen = 'green'
n29_pen = 'cyan'

# legends
n8_leg = '0.66 mm (N8)'
n14_leg = '0.84 mm (N14)'
n5_leg = '1.20 mm (N5)'
n23a_leg = '1.68 mm (N23a)'
n29_leg = '1.89 mm (N29)'
```

```
Sheet Names 1: ['lin-lin & graphs', 'octave scalers', 'act.notnl.stress', 'Eb', 'E vs. rho', 'dF.dT', 'df.dT_L study', 'df.dT_L', 'df.dT_F']
```

## Read data for fluorocarbon strings¶

In [3]:

```
# READ DATA FOR FLUOROCARBON STRINGS
# connect to data source
file_location = "./fluorocarbon master data file.xls"
workbook3 = xlrd.open_workbook(file_location)
data_sheet = workbook3.sheet_by_index(0)
#
# List sheet names
sheet_names = workbook3.sheet_names()
print('Sheet Names 3:', sheet_names)
#
# define data arrays
# Note: use zero-indexing for row and column numbers
# row spans
c1_start_row = 6
c1_stop_row = 11
c3_start_row = 23
c3_stop_row = 28
c5b_start_row = 57
c5b_stop_row = 62
c7a_start_row = 74
c7a_stop_row = 78
cop_range = [27,60,76]
# columns
rho_n_col = 13
Fn_col = 14
Fw_col = 23
F0_col = 49
stress_n_col = 15
stress_col = 24
strain_col = 18
Et_col = 35
Eb_col = 41
Es_col = 32
dfdT_L_col = 52
dfdT_F_col = 55
dFdT_col = 48
dEtdT_col = 36
dEbdT_col = 40
CLTE_col = 43
psi_col = 51
den_ratio_col = 64
den_err_plus_col = 65
den_err_minus_col = 66
#
# c1 - Savarez 0.57 mm, A6
c1_rho_n = [data_sheet.cell_value(c1_start_row, rho_n_col) for i in range(c1_start_row, c1_stop_row)]
c1_Fn = [data_sheet.cell_value(i, Fn_col) for i in range(c1_start_row, c1_stop_row)]
c1_Fw = [data_sheet.cell_value(i, Fw_col) for i in range(c1_start_row, c1_stop_row)]
c1_F0 = [data_sheet.cell_value(i, F0_col) for i in range(c1_start_row, c1_stop_row)]
c1_stress_n = [data_sheet.cell_value(i, stress_n_col) for i in range(c1_start_row, c1_stop_row)]
c1_stress = [data_sheet.cell_value(i, stress_col) for i in range(c1_start_row, c1_stop_row)]
c1_strain = [data_sheet.cell_value(i, strain_col) for i in range(c1_start_row, c1_stop_row)]
c1_Et = [data_sheet.cell_value(i, Et_col) for i in range(c1_start_row, c1_stop_row)]
c1_Eb = [data_sheet.cell_value(i, Eb_col) for i in range(c1_start_row, c1_stop_row)]
c1_Es = [data_sheet.cell_value(i, Es_col) for i in range(c1_start_row, c1_stop_row)]
c1_dfdT_L = [data_sheet.cell_value(i, dfdT_L_col) for i in range(c1_start_row, c1_stop_row)]
c1_dfdT_F = [data_sheet.cell_value(i, dfdT_F_col) for i in range(c1_start_row, c1_stop_row)]
c1_dFdT = [data_sheet.cell_value(i, dFdT_col) for i in range(c1_start_row, c1_stop_row)]
c1_dEtdT = [data_sheet.cell_value(i, dEtdT_col) for i in range(c1_start_row, c1_stop_row)]
c1_dEbdT = [data_sheet.cell_value(i, dEbdT_col) for i in range(c1_start_row, c1_stop_row)]
c1_CLTE = [(data_sheet.cell_value(i, CLTE_col)*1e6) for i in range(c1_start_row, c1_stop_row)]
c1_psi = [(data_sheet.cell_value(i, psi_col)*1000) for i in range(c1_start_row, c1_stop_row)]
#
c1_stress0 = [data_sheet.cell_value(i, stress_col) for i in range(c1_start_row-1, c1_stop_row)]
c1_strain0 = [data_sheet.cell_value(i, strain_col) for i in range(c1_start_row-1, c1_stop_row)]
c1_den_ratio = [data_sheet.cell_value(i, den_ratio_col) for i in range(c1_start_row-1, c1_stop_row)]
c1_den_err_plus = [data_sheet.cell_value(i, den_err_plus_col) for i in range(c1_start_row-1, c1_stop_row)]
c1_den_err_minus = [data_sheet.cell_value(i, den_err_minus_col) for i in range(c1_start_row-1, c1_stop_row)]
#
# c3 - Savarez 0.81 mm, A5
c3_rho_n = [data_sheet.cell_value(c3_start_row, rho_n_col) for i in range(c3_start_row, c3_stop_row)]
c3_Fn = [data_sheet.cell_value(i, Fn_col) for i in range(c3_start_row, c3_stop_row)]
c3_Fw = [data_sheet.cell_value(i, Fw_col) for i in range(c3_start_row, c3_stop_row)]
c3_F0 = [data_sheet.cell_value(i, F0_col) for i in range(c3_start_row, c3_stop_row)]
c3_stress_n = [data_sheet.cell_value(i, stress_n_col) for i in range(c3_start_row, c3_stop_row)]
c3_stress = [data_sheet.cell_value(i, stress_col) for i in range(c3_start_row, c3_stop_row)]
c3_strain = [data_sheet.cell_value(i, strain_col) for i in range(c3_start_row, c3_stop_row)]
c3_Et = [data_sheet.cell_value(i, Et_col) for i in range(c3_start_row, c3_stop_row)]
c3_Eb = [data_sheet.cell_value(i, Eb_col) for i in range(c3_start_row, c3_stop_row)]
c3_Es = [data_sheet.cell_value(i, Es_col) for i in range(c3_start_row, c3_stop_row)]
c3_dfdT_L = [data_sheet.cell_value(i, dfdT_L_col) for i in range(c3_start_row, c3_stop_row)]
c3_dfdT_F = [data_sheet.cell_value(i, dfdT_F_col) for i in range(c3_start_row, c3_stop_row)]
c3_dFdT = [data_sheet.cell_value(i, dFdT_col) for i in range(c3_start_row, c3_stop_row)]
c3_dEtdT = [data_sheet.cell_value(i, dEtdT_col) for i in range(c3_start_row, c3_stop_row)]
c3_dEbdT = [data_sheet.cell_value(i, dEbdT_col) for i in range(c3_start_row, c3_stop_row)]
c3_CLTE = [(data_sheet.cell_value(i, CLTE_col)*1e6) for i in range(c3_start_row, c3_stop_row)]
c3_psi = [(data_sheet.cell_value(i, psi_col)*1000) for i in range(c3_start_row, c3_stop_row)]
#
c3_stress0 = [data_sheet.cell_value(i, stress_col) for i in range(c3_start_row-1, c3_stop_row)]
c3_strain0 = [data_sheet.cell_value(i, strain_col) for i in range(c3_start_row-1, c3_stop_row)]
c3_den_ratio = [data_sheet.cell_value(i, den_ratio_col) for i in range(c3_start_row-1, c3_stop_row)]
c3_den_err_plus = [data_sheet.cell_value(i, den_err_plus_col) for i in range(c3_start_row-1, c3_stop_row)]
c3_den_err_minus = [data_sheet.cell_value(i, den_err_minus_col) for i in range(c3_start_row-1, c3_stop_row)]
#
# c5b - Savarez 1.08 mm, A4
c5b_rho_n = [data_sheet.cell_value(c5b_start_row, rho_n_col) for i in range(c5b_start_row, c5b_stop_row)]
c5b_Fn = [data_sheet.cell_value(i, Fn_col) for i in range(c5b_start_row, c5b_stop_row)]
c5b_Fw = [data_sheet.cell_value(i, Fw_col) for i in range(c5b_start_row, c5b_stop_row)]
c5b_F0 = [data_sheet.cell_value(i, F0_col) for i in range(c5b_start_row, c5b_stop_row)]
c5b_stress_n = [data_sheet.cell_value(i, stress_n_col) for i in range(c5b_start_row, c5b_stop_row)]
c5b_stress = [data_sheet.cell_value(i, stress_col) for i in range(c5b_start_row, c5b_stop_row)]
c5b_strain = [data_sheet.cell_value(i, strain_col) for i in range(c5b_start_row, c5b_stop_row)]
c5b_Et = [data_sheet.cell_value(i, Et_col) for i in range(c5b_start_row, c5b_stop_row)]
c5b_Eb = [data_sheet.cell_value(i, Eb_col) for i in range(c5b_start_row, c5b_stop_row)]
c5b_Es = [data_sheet.cell_value(i, Es_col) for i in range(c5b_start_row, c5b_stop_row)]
c5b_dfdT_L = [data_sheet.cell_value(i, dfdT_L_col) for i in range(c5b_start_row, c5b_stop_row)]
c5b_dfdT_F = [data_sheet.cell_value(i, dfdT_F_col) for i in range(c5b_start_row, c5b_stop_row)]
c5b_dFdT = [data_sheet.cell_value(i, dFdT_col) for i in range(c5b_start_row, c5b_stop_row)]
c5b_dEtdT = [data_sheet.cell_value(i, dEtdT_col) for i in range(c5b_start_row, c5b_stop_row)]
c5b_dEbdT = [data_sheet.cell_value(i, dEbdT_col) for i in range(c5b_start_row, c5b_stop_row)]
c5b_CLTE = [(data_sheet.cell_value(i, CLTE_col)*1e6) for i in range(c5b_start_row, c5b_stop_row)]
c5b_psi = [(data_sheet.cell_value(i, psi_col)*1000) for i in range(c5b_start_row, c5b_stop_row)]
#
c5b_stress0 = [data_sheet.cell_value(i, stress_col) for i in range(c5b_start_row-1, c5b_stop_row)]
c5b_strain0 = [data_sheet.cell_value(i, strain_col) for i in range(c5b_start_row-1, c5b_stop_row)]
c5b_den_ratio = [data_sheet.cell_value(i, den_ratio_col) for i in range(c5b_start_row-1, c5b_stop_row)]
c5b_den_err_plus = [data_sheet.cell_value(i, den_err_plus_col) for i in range(c5b_start_row-1, c5b_stop_row)]
c5b_den_err_minus = [data_sheet.cell_value(i, den_err_minus_col) for i in range(c5b_start_row-1, c5b_stop_row)]
#
# c7a - Savarez 1.50 mm, A3
c7a_rho_n = [data_sheet.cell_value(c7a_start_row, rho_n_col) for i in range(c7a_start_row, c7a_stop_row)]
c7a_Fn = [data_sheet.cell_value(i, Fn_col) for i in range(c7a_start_row, c7a_stop_row)]
c7a_Fw = [data_sheet.cell_value(i, Fw_col) for i in range(c7a_start_row, c7a_stop_row)]
c7a_F0 = [data_sheet.cell_value(i, F0_col) for i in range(c7a_start_row, c7a_stop_row)]
c7a_stress_n = [data_sheet.cell_value(i, stress_n_col) for i in range(c7a_start_row, c7a_stop_row)]
c7a_stress = [data_sheet.cell_value(i, stress_col) for i in range(c7a_start_row, c7a_stop_row)]
c7a_strain = [data_sheet.cell_value(i, strain_col) for i in range(c7a_start_row, c7a_stop_row)]
c7a_Et = [data_sheet.cell_value(i, Et_col) for i in range(c7a_start_row, c7a_stop_row)]
c7a_Eb = [data_sheet.cell_value(i, Eb_col) for i in range(c7a_start_row, c7a_stop_row)]
c7a_Es = [data_sheet.cell_value(i, Es_col) for i in range(c7a_start_row, c7a_stop_row)]
c7a_dfdT_L = [data_sheet.cell_value(i, dfdT_L_col) for i in range(c7a_start_row, c7a_stop_row)]
c7a_dfdT_F = [data_sheet.cell_value(i, dfdT_F_col) for i in range(c7a_start_row, c7a_stop_row)]
c7a_dFdT = [data_sheet.cell_value(i, dFdT_col) for i in range(c7a_start_row, c7a_stop_row)]
c7a_dEtdT = [data_sheet.cell_value(i, dEtdT_col) for i in range(c7a_start_row, c7a_stop_row)]
c7a_dEbdT = [data_sheet.cell_value(i, dEbdT_col) for i in range(c7a_start_row, c7a_stop_row)]
c7a_CLTE = [(data_sheet.cell_value(i, CLTE_col)*1e6) for i in range(c7a_start_row, c7a_stop_row)]
c7a_psi = [(data_sheet.cell_value(i, psi_col)*1000) for i in range(c7a_start_row, c7a_stop_row)]
#
c7a_stress0 = [data_sheet.cell_value(i, stress_col) for i in range(c7a_start_row-1, c7a_stop_row)]
c7a_strain0 = [data_sheet.cell_value(i, strain_col) for i in range(c7a_start_row-1, c7a_stop_row)]
c7a_den_ratio = [data_sheet.cell_value(i, den_ratio_col) for i in range(c7a_start_row-1, c7a_stop_row)]
c7a_den_err_plus = [data_sheet.cell_value(i, den_err_plus_col) for i in range(c7a_start_row-1, c7a_stop_row)]
c7a_den_err_minus = [data_sheet.cell_value(i, den_err_minus_col) for i in range(c7a_start_row-1, c7a_stop_row)]
#
# operating points
cop_Fn = [data_sheet.cell_value(i, Fn_col) for i in cop_range]
cop_Fw = [data_sheet.cell_value(i, Fw_col) for i in cop_range]
cop_stress = [data_sheet.cell_value(i, stress_col) for i in cop_range]
cop_dfdT_L = [data_sheet.cell_value(i, dfdT_L_col) for i in cop_range]
cop_dfdT_F = [data_sheet.cell_value(i, dfdT_F_col) for i in cop_range]

# inverse stress values
c1_inv_stress = 1.0 / np.asarray(c1_stress)
c3_inv_stress = 1.0 / np.asarray(c3_stress)
c5b_inv_stress = 1.0 / np.asarray(c5b_stress)
c7a_inv_stress = 1.0 / np.asarray(c7a_stress)
cop_inv_stress = 1.0 / np.asarray(cop_stress)

# Es stress values
c1_Es_stress = []
for i in range (0, len(c1_Es), 1):
    c1_Es_stress.append((c1_stress0[i]+c1_stress0[i+1])/2.0)
c3_Es_stress = []
for i in range (0, len(c3_Es), 1):
    c3_Es_stress.append((c3_stress0[i]+c3_stress0[i+1])/2.0)
c5b_Es_stress = []
for i in range (0, len(c5b_Es), 1):
    c5b_Es_stress.append((c5b_stress0[i]+c5b_stress0[i+1])/2.0)
c7a_Es_stress = []
for i in range (0, len(c7a_Es), 1):
    c7a_Es_stress.append((c7a_stress0[i]+c7a_stress0[i+1])/2.0)

# (1/F).dF/dT
c1_dFdTbyF = 1000.0 * np.asarray(c1_dFdT) / np.asarray(c1_F0)
c3_dFdTbyF = 1000.0 * np.asarray(c3_dFdT) / np.asarray(c3_F0)
c5b_dFdTbyF = 1000.0 * np.asarray(c5b_dFdT) / np.asarray(c5b_F0)
c7a_dFdTbyF = 1000.0 * np.asarray(c7a_dFdT) / np.asarray(c7a_F0)

# plot colours
c1_pen = 'black'
c3_pen = 'red'
c5b_pen = 'blue'
c7a_pen = 'green'

# legends
c1_leg = '0.58 mm (C1)'
c3_leg = '0.82 mm (C3)'
c5b_leg = '1.03 mm (C5b)'
c7a_leg = '1.49 mm (C7a)'
```

```
Sheet Names 3: ['lin-lin & graphs', 'E']
```

## Read data for gut¶

In [4]:

```
# READ DATA FOR GUT
# connect to data source
file_location = "./gut master data file.xls"
workbook = xlrd.open_workbook(file_location)
data_sheet = workbook.sheet_by_index(0)
bell_firth = workbook.sheet_by_index(1)
#
# List sheet names
sheet_names = workbook.sheet_names()
print('Sheet Names', sheet_names)
#
#
# define data arrays
# Note: use zero-indexing for row and column numbers
# row spans
g2b_start_row = 15
g2b_stop_row = 21
g3b_start_row = 32
g3b_stop_row = 39
g3c_start_row = 50
g3c_stop_row = 56
g5b_start_row = 68
g5b_stop_row = 72
g5c_start_row = 86
g5c_stop_row = 91
gop_range = [19,35,53,70,88]
# columns
inv_d_col = 8
Fn_col = 13
Fw_col = 23
F0_col = 45
stress_col = 24
strain_col = 18
Et_col = 30
Eb_man_col = 35
Es_col = 28
dfdT_L_col = 53
dfdT_F_col = 56
dFdT_col = 44
dEtdT_col = 31
CLTE_col = 41
psi_col = 52
den_ratio_col = 65
den_err_plus_col = 66
den_err_minus_col = 67

# G2b - A5
g2b_Fn = [data_sheet.cell_value(i, Fn_col) for i in range(g2b_start_row, g2b_stop_row)]
g2b_Fw = [data_sheet.cell_value(i, Fw_col) for i in range(g2b_start_row, g2b_stop_row)]
g2b_F0 = [data_sheet.cell_value(i, F0_col) for i in range(g2b_start_row, g2b_stop_row)]
g2b_stress = [data_sheet.cell_value(i, stress_col) for i in range(g2b_start_row, g2b_stop_row)]
g2b_strain = [data_sheet.cell_value(i, strain_col) for i in range(g2b_start_row, g2b_stop_row)]
g2b_Et = [data_sheet.cell_value(i, Et_col) for i in range(g2b_start_row, g2b_stop_row)]
g2b_Eb_man = [data_sheet.cell_value(i, Eb_man_col) for i in range(g2b_start_row, g2b_stop_row)]
g2b_Es = [data_sheet.cell_value(i, Es_col) for i in range(g2b_start_row, g2b_stop_row)]
g2b_dfdT_L = [data_sheet.cell_value(i, dfdT_L_col) for i in range(g2b_start_row, g2b_stop_row)]
g2b_dfdT_F = [data_sheet.cell_value(i, dfdT_F_col) for i in range(g2b_start_row, g2b_stop_row)]
g2b_dFdT = [data_sheet.cell_value(i, dFdT_col) for i in range(g2b_start_row, g2b_stop_row)]
g2b_dEtdT = [data_sheet.cell_value(i, dEtdT_col) for i in range(g2b_start_row, g2b_stop_row)]
g2b_CLTE = [(data_sheet.cell_value(i, CLTE_col)*1e6) for i in range(g2b_start_row, g2b_stop_row)]
g2b_psi = [(data_sheet.cell_value(i, psi_col)*1000) for i in range(g2b_start_row, g2b_stop_row)]
#
g2b_stress0 = [data_sheet.cell_value(i, stress_col) for i in range(g2b_start_row-1, g2b_stop_row)]
g2b_strain0 = [data_sheet.cell_value(i, strain_col) for i in range(g2b_start_row-1, g2b_stop_row)]
g2b_den_ratio = [data_sheet.cell_value(i, den_ratio_col) for i in range(g2b_start_row-1, g2b_stop_row)]
g2b_den_err_plus = [data_sheet.cell_value(i, den_err_plus_col) for i in range(g2b_start_row-1, g2b_stop_row)]
g2b_den_err_minus = [data_sheet.cell_value(i, den_err_minus_col) for i in range(g2b_start_row-1, g2b_stop_row)]
#
# G3b - A4
g3b_Fn = [data_sheet.cell_value(i, Fn_col) for i in range(g3b_start_row, g3b_stop_row)]
g3b_Fw = [data_sheet.cell_value(i, Fw_col) for i in range(g3b_start_row, g3b_stop_row)]
g3b_F0 = [data_sheet.cell_value(i, F0_col) for i in range(g3b_start_row, g3b_stop_row)]
g3b_stress = [data_sheet.cell_value(i, stress_col) for i in range(g3b_start_row, g3b_stop_row)]
g3b_strain = [data_sheet.cell_value(i, strain_col) for i in range(g3b_start_row, g3b_stop_row)]
g3b_Et = [data_sheet.cell_value(i, Et_col) for i in range(g3b_start_row, g3b_stop_row)]
g3b_Eb_man = [data_sheet.cell_value(i, Eb_man_col) for i in range(g3b_start_row, g3b_stop_row)]
g3b_Eb_stress = [data_sheet.cell_value(i, stress_col) for i in range(g3b_start_row+2, g3b_stop_row)]
g3b_Eb_strain = [data_sheet.cell_value(i, strain_col) for i in range(g3b_start_row+2, g3b_stop_row)]
g3b_Es = [data_sheet.cell_value(i, Es_col) for i in range(g3b_start_row, g3b_stop_row)]
g3b_dfdT_L = [data_sheet.cell_value(i, dfdT_L_col) for i in range(g3b_start_row, g3b_stop_row)]
g3b_dfdT_F = [data_sheet.cell_value(i, dfdT_F_col) for i in range(g3b_start_row, g3b_stop_row)]
g3b_dFdT = [data_sheet.cell_value(i, dFdT_col) for i in range(g3b_start_row, g3b_stop_row)]
g3b_dEtdT = [data_sheet.cell_value(i, dEtdT_col) for i in range(g3b_start_row, g3b_stop_row)]
g3b_CLTE = [(data_sheet.cell_value(i, CLTE_col)*1e6) for i in range(g3b_start_row, g3b_stop_row)]
g3b_psi = [(data_sheet.cell_value(i, psi_col)*1000) for i in range(g3b_start_row, g3b_stop_row)]
#
g3b_stress0 = [data_sheet.cell_value(i, stress_col) for i in range(g3b_start_row-1, g3b_stop_row)]
g3b_strain0 = [data_sheet.cell_value(i, strain_col) for i in range(g3b_start_row-1, g3b_stop_row)]
g3b_den_ratio = [data_sheet.cell_value(i, den_ratio_col) for i in range(g3b_start_row-1, g3b_stop_row)]
g3b_den_err_plus = [data_sheet.cell_value(i, den_err_plus_col) for i in range(g3b_start_row-1, g3b_stop_row)]
g3b_den_err_minus = [data_sheet.cell_value(i, den_err_minus_col) for i in range(g3b_start_row-1, g3b_stop_row)]
#
# G3c - A4
g3c_Fn = [data_sheet.cell_value(i, Fn_col) for i in range(g3c_start_row, g3c_stop_row)]
g3c_Fw = [data_sheet.cell_value(i, Fw_col) for i in range(g3c_start_row, g3c_stop_row)]
g3c_F0 = [data_sheet.cell_value(i, F0_col) for i in range(g3c_start_row, g3c_stop_row)]
g3c_stress = [data_sheet.cell_value(i, stress_col) for i in range(g3c_start_row, g3c_stop_row)]
g3c_strain = [data_sheet.cell_value(i, strain_col) for i in range(g3c_start_row, g3c_stop_row)]
g3c_Et = [data_sheet.cell_value(i, Et_col) for i in range(g3c_start_row, g3c_stop_row)]
g3c_Eb_man = [data_sheet.cell_value(i, Eb_man_col) for i in range(g3c_start_row, g3c_stop_row)]
g3c_Es = [data_sheet.cell_value(i, Es_col) for i in range(g3c_start_row, g3c_stop_row)]
g3c_dfdT_L = [data_sheet.cell_value(i, dfdT_L_col) for i in range(g3c_start_row, g3c_stop_row)]
g3c_dfdT_F = [data_sheet.cell_value(i, dfdT_F_col) for i in range(g3c_start_row, g3c_stop_row)]
g3c_dFdT = [data_sheet.cell_value(i, dFdT_col) for i in range(g3c_start_row, g3c_stop_row)]
g3c_dEtdT = [data_sheet.cell_value(i, dEtdT_col) for i in range(g3c_start_row, g3c_stop_row)]
g3c_CLTE = [(data_sheet.cell_value(i, CLTE_col)*1e6) for i in range(g3c_start_row, g3c_stop_row)]
g3c_psi = [(data_sheet.cell_value(i, psi_col)*1000) for i in range(g3c_start_row, g3c_stop_row)]
#
g3c_stress0 = [data_sheet.cell_value(i, stress_col) for i in range(g3c_start_row-1, g3c_stop_row)]
g3c_strain0 = [data_sheet.cell_value(i, strain_col) for i in range(g3c_start_row-1, g3c_stop_row)]
g3c_den_ratio = [data_sheet.cell_value(i, den_ratio_col) for i in range(g3c_start_row-1, g3c_stop_row)]
g3c_den_err_plus = [data_sheet.cell_value(i, den_err_plus_col) for i in range(g3c_start_row-1, g3c_stop_row)]
g3c_den_err_minus = [data_sheet.cell_value(i, den_err_minus_col) for i in range(g3c_start_row-1, g3c_stop_row)]
#
# G5b - A3
g5b_Fn = [data_sheet.cell_value(i, Fn_col) for i in range(g5b_start_row, g5b_stop_row)]
g5b_Fw = [data_sheet.cell_value(i, Fw_col) for i in range(g5b_start_row, g5b_stop_row)]
g5b_F0 = [data_sheet.cell_value(i, F0_col) for i in range(g5b_start_row, g5b_stop_row)]
g5b_stress = [data_sheet.cell_value(i, stress_col) for i in range(g5b_start_row, g5b_stop_row)]
g5b_strain = [data_sheet.cell_value(i, strain_col) for i in range(g5b_start_row, g5b_stop_row)]
g5b_Et = [data_sheet.cell_value(i, Et_col) for i in range(g5b_start_row, g5b_stop_row)]
g5b_Eb_man = [data_sheet.cell_value(i, Eb_man_col) for i in range(g5b_start_row, g5b_stop_row)]
g5b_Es = [data_sheet.cell_value(i, Es_col) for i in range(g5b_start_row, g5b_stop_row)]
g5b_dfdT_L = [data_sheet.cell_value(i, dfdT_L_col) for i in range(g5b_start_row, g5b_stop_row)]
g5b_dfdT_F = [data_sheet.cell_value(i, dfdT_F_col) for i in range(g5b_start_row, g5b_stop_row)]
g5b_dFdT = [data_sheet.cell_value(i, dFdT_col) for i in range(g5b_start_row, g5b_stop_row)]
g5b_dEtdT = [data_sheet.cell_value(i, dEtdT_col) for i in range(g5b_start_row, g5b_stop_row)]
g5b_CLTE = [(data_sheet.cell_value(i, CLTE_col)*1e6) for i in range(g5b_start_row, g5b_stop_row)]
g5b_psi = [(data_sheet.cell_value(i, psi_col)*1000) for i in range(g5b_start_row, g5b_stop_row)]
#
g5b_stress0 = [data_sheet.cell_value(i, stress_col) for i in range(g5b_start_row-1, g5b_stop_row)]
g5b_strain0 = [data_sheet.cell_value(i, strain_col) for i in range(g5b_start_row-1, g5b_stop_row)]
g5b_den_ratio = [data_sheet.cell_value(i, den_ratio_col) for i in range(g5b_start_row-1, g5b_stop_row)]
g5b_den_err_plus = [data_sheet.cell_value(i, den_err_plus_col) for i in range(g5b_start_row-1, g5b_stop_row)]
g5b_den_err_minus = [data_sheet.cell_value(i, den_err_minus_col) for i in range(g5b_start_row-1, g5b_stop_row)]
#
# G5c - A3
g5c_Fn = [data_sheet.cell_value(i, Fn_col) for i in range(g5c_start_row, g5c_stop_row)]
g5c_Fw = [data_sheet.cell_value(i, Fw_col) for i in range(g5c_start_row, g5c_stop_row)]
g5c_F0 = [data_sheet.cell_value(i, F0_col) for i in range(g5c_start_row, g5c_stop_row)]
g5c_stress = [data_sheet.cell_value(i, stress_col) for i in range(g5c_start_row, g5c_stop_row)]
g5c_strain = [data_sheet.cell_value(i, strain_col) for i in range(g5c_start_row, g5c_stop_row)]
g5c_Et = [data_sheet.cell_value(i, Et_col) for i in range(g5c_start_row, g5c_stop_row)]
g5c_Eb_man = [data_sheet.cell_value(i, Eb_man_col) for i in range(g5c_start_row, g5c_stop_row)]
g5c_Es = [data_sheet.cell_value(i, Es_col) for i in range(g5c_start_row, g5c_stop_row)]
g5c_dfdT_L = [data_sheet.cell_value(i, dfdT_L_col) for i in range(g5c_start_row, g5c_stop_row)]
g5c_dfdT_F = [data_sheet.cell_value(i, dfdT_F_col) for i in range(g5c_start_row, g5c_stop_row)]
g5c_dFdT = [data_sheet.cell_value(i, dFdT_col) for i in range(g5c_start_row, g5c_stop_row)]
g5c_dEtdT = [data_sheet.cell_value(i, dEtdT_col) for i in range(g5c_start_row, g5c_stop_row)]
g5c_CLTE = [(data_sheet.cell_value(i, CLTE_col)*1e6) for i in range(g5c_start_row, g5c_stop_row)]
g5c_psi = [(data_sheet.cell_value(i, psi_col)*1000) for i in range(g5c_start_row, g5c_stop_row)]
#
g5c_stress0 = [data_sheet.cell_value(i, stress_col) for i in range(g5c_start_row-1, g5c_stop_row)]
g5c_strain0 = [data_sheet.cell_value(i, strain_col) for i in range(g5c_start_row-1, g5c_stop_row)]
g5c_den_ratio = [data_sheet.cell_value(i, den_ratio_col) for i in range(g5c_start_row-1, g5c_stop_row)]
g5c_den_err_plus = [data_sheet.cell_value(i, den_err_plus_col) for i in range(g5c_start_row-1, g5c_stop_row)]
g5c_den_err_minus = [data_sheet.cell_value(i, den_err_minus_col) for i in range(g5c_start_row-1, g5c_stop_row)]
#
# Operating points
gop_Fn = [data_sheet.cell_value(i, Fn_col) for i in gop_range]
gop_Fw = [data_sheet.cell_value(i, Fw_col) for i in gop_range]
gop_stress = [data_sheet.cell_value(i, stress_col) for i in gop_range]
gop_inv_d = [data_sheet.cell_value(i, inv_d_col) for i in gop_range]
gop_Et = [data_sheet.cell_value(i, Et_col) for i in gop_range]
gop_Eb_man = [data_sheet.cell_value(i, Eb_man_col) for i in gop_range]
gop_dfdT_L = [data_sheet.cell_value(i, dfdT_L_col) for i in gop_range]
gop_dfdT_F = [data_sheet.cell_value(i, dfdT_F_col) for i in gop_range]

avg_Es = []
avg_Es.append(np.average(g2b_Es))
avg_Es.append(np.average(g3b_Es))
avg_Es.append(np.average(g3c_Es))
avg_Es.append(np.average(g5b_Es))
avg_Es.append(np.average(g5c_Es[:-1]))

avg_Et = []
avg_Et.append(np.average(g2b_Et))
avg_Et.append(np.average(g3b_Et))
avg_Et.append(np.average(g3c_Et))
avg_Et.append(np.average(g5b_Et))
avg_Et.append(np.average(g5c_Et[:-1]))

avg_Eb_man = []
avg_Eb_man.append(np.average(g2b_Eb_man))
avg_Eb_man.append(np.average(g3b_Eb_man))
avg_Eb_man.append(np.average(g3c_Eb_man))
avg_Eb_man.append(np.average(g5b_Eb_man))
avg_Eb_man.append(np.average(g5c_Eb_man[:-1]))

# inverse stress values
g2b_inv_stress = 1.0 / np.asarray(g2b_stress)
g3b_inv_stress = 1.0 / np.asarray(g3b_stress)
g3c_inv_stress = 1.0 / np.asarray(g3c_stress)
g5b_inv_stress = 1.0 / np.asarray(g5b_stress)
g5c_inv_stress = 1.0 / np.asarray(g5c_stress)
gop_inv_stress = 1.0 / np.asarray(gop_stress)

# Es stress values
g2b_Es_stress = []
for i in range (0, len(g2b_Es), 1):
    g2b_Es_stress.append((g2b_stress0[i]+g2b_stress0[i+1])/2.0)
g3b_Es_stress = []
for i in range (0, len(g3b_Es), 1):
    g3b_Es_stress.append((g3b_stress0[i]+g3b_stress0[i+1])/2.0)
g3c_Es_stress = []
for i in range (0, len(g3c_Es), 1):
    g3c_Es_stress.append((g3c_stress0[i]+g3c_stress0[i+1])/2.0)
g5b_Es_stress = []
for i in range (0, len(g5b_Es), 1):
    g5b_Es_stress.append((g5b_stress0[i]+g5b_stress0[i+1])/2.0)
g5c_Es_stress = []
for i in range (0, len(g5c_Es), 1):
    g5c_Es_stress.append((g5c_stress0[i]+g5c_stress0[i+1])/2.0)
    
# (1/F).dF/dT
g2b_dFdTbyF = 1000.0 * np.asarray(g2b_dFdT) / np.asarray(g2b_F0)
g3b_dFdTbyF = 1000.0 * np.asarray(g3b_dFdT) / np.asarray(g3b_F0)
g3c_dFdTbyF = 1000.0 * np.asarray(g3c_dFdT) / np.asarray(g3c_F0)
g5b_dFdTbyF = 1000.0 * np.asarray(g5b_dFdT) / np.asarray(g5b_F0)
g5c_dFdTbyF = 1000.0 * np.asarray(g5c_dFdT) / np.asarray(g5c_F0)

# plot colours
g2b_pen = 'red'
g3b_pen = 'blue'
g3c_pen = 'blue'
g5b_pen = 'green'
g5c_pen = 'green'

# legends
g2b_leg = '0.84 mm (G2b)'
g3b_leg = '1.17 mm (G3b)'
g3c_leg = '1.17 mm (G3c)'
g3_leg = '1.17 mm (G3b/c)'
g5b_leg = '1.63 mm (G5b)'
g5c_leg = '1.63 mm (G5c)'
g5_leg = '1.63 mm (G5b/c)'


# Bell & Firth data
bf_inv_d = [bell_firth.cell_value(i, 9) for i in range(3,11)]
bf_Et = [bell_firth.cell_value(i, 6) for i in range(3,11)]
bf_Et_SD = [bell_firth.cell_value(i, 7) for i in range(3,11)]
bf_weights = [bell_firth.cell_value(i, 1) for i in range(3,11)]
```

```
Sheet Names ['lin-lin & graphs', 'Bell.Firth']
```

## Stress vs. strain¶

In [5]:

```
fig=plt.figure(figsize=(12,3.9))
axis_limits = [0, 0.22, 0, 270]
x_tick_spec = np.arange(0, 0.25, 0.05)
y_tick_spec = np.arange(0, 300, 50)
# Ground nylon
ax1=fig.add_subplot(131)
ax1.plot(n8_strain0, n8_stress0, color=n8_pen, lw=1, marker='*', mec=n8_pen, mfc='none', mew=1, label=n8_leg)
ax1.plot(n14_strain0, n14_stress0, color=n14_pen, lw=1, marker='*', mec=n14_pen, mfc='none', mew=1, label=n14_leg)
ax1.plot(n5_strain0, n5_stress0, color=n5_pen, lw=1, marker='*', mec=n5_pen, mfc='none', mew=1, label=n5_leg)
ax1.plot(n23a_strain0, n23a_stress0, color=n23a_pen, lw=1, marker='*', mec=n23a_pen, mfc='none', mew=1, label=n23a_leg)
ax1.plot(n29_strain0, n29_stress0, color=n29_pen, lw=1, marker='*', mec=n29_pen, mfc='none', mew=1, label=n29_leg)
ax1.legend(loc='best', ncol = 1, prop={'size':8})
ax1.axis(axis_limits)
ax1.set_xticks(x_tick_spec)
ax1.set_yticks(y_tick_spec)
ax1.set_xlabel('Strain')
ax1.set_ylabel('Stress (MPa)')
# Carbon
ax2=fig.add_subplot(132)
ax2.plot(c1_strain0, c1_stress0, color=c1_pen, lw=1, marker='*', mec=c1_pen, mfc='none', mew=1, label=c1_leg)
ax2.plot(c3_strain0, c3_stress0, color=c3_pen, lw=1, marker='*', mec=c3_pen, mfc='none', mew=1, label=c3_leg)
ax2.plot(c5b_strain0, c5b_stress0, color=c5b_pen, lw=1, marker='*', mec=c5b_pen, mfc='none', mew=1, label=c5b_leg)
ax2.plot(c7a_strain0, c7a_stress0, color=c7a_pen, lw=1, marker='*', mec=c7a_pen, mfc='none', mew=1, label=c7a_leg)
ax2.legend(loc='best', ncol = 1, prop={'size':8})
ax2.axis(axis_limits)
ax2.set_xticks(x_tick_spec)
ax2.set_yticks(y_tick_spec)
ax2.set_xlabel('Strain')
ax2.set_ylabel('Stress (MPa)')
# Gut
ax3=fig.add_subplot(133)
ax3.plot(g2b_strain0, g2b_stress0, color=g2b_pen, lw=1, marker='*', mec=g2b_pen, mfc='none', mew=1, label=g2b_leg)
ax3.plot(g3b_strain0, g3b_stress0, color=g3b_pen, lw=1, marker='*', mec=g3b_pen, mfc='none', mew=1, label=g3_leg)
ax3.plot(g3c_strain0, g3c_stress0, color=g3c_pen, lw=1, marker='*', mec=g3c_pen, mfc='none', mew=1)
ax3.plot(g5b_strain0, g5b_stress0, color=g5b_pen, lw=1, marker='*', mec=g5b_pen, mfc='none', mew=1, label=g5_leg)
ax3.plot(g5c_strain0[:-1], g5c_stress0[:-1], color=g5c_pen, lw=1, marker='*', mec=g5c_pen, mfc='none', mew=1)
ax3.legend(loc='lower right', ncol = 1, prop={'size':8})
ax3.axis(axis_limits)
ax3.set_xticks(x_tick_spec)
ax3.set_yticks(y_tick_spec)
ax3.set_xlabel('Strain')
ax3.set_ylabel('Stress (MPa)')
#
plt.tight_layout()
ax1.text(0.87, 0.9,'(a)', transform=ax1.transAxes)
ax2.text(0.87, 0.9,'(b)', transform=ax2.transAxes)
ax3.text(0.87, 0.9,'(c)', transform=ax3.transAxes)
plt.savefig('./01abc_stress_strain.eps', format='eps', dpi=1000)
plt.show()
```

## Gut stress-strain gradients¶

In [6]:

```
# Notes on regression through the origin
# see: https://online.stat.psu.edu/~ajw13/stat501/SpecialTopics/Reg_thru_origin.pdf
# Joseph G. Eisenhauer. Regression through the Origin. Teaching Statistics. Volume 25, Number 3, pp. 76-80, Autumn 2003
# 
# slope of least squares fit line through origin, y_est = a.x
# a = sum(x.y)/sum(x**2) = x.dot(y)/x.dot(x)
#
# for the coefficient of determination for regression through the origin:
# r^2 = sum(y_est**2)/sum(y**2) = y_est.dot(y_est)/y.dot(y) = a**2 * x.dot(x)/y.dot(y)

# G2b
x = np.asarray(g2b_strain0)
y = np.asarray(g2b_stress0)
a = x.dot(y)/x.dot(x)   # slope of least squares fit line through origin, y = a.x
r_sq = a**2 * x.dot(x)/y.dot(y)
print('G2b:', a, r_sq)
# G3b
x = np.asarray(g3b_strain0)
y = np.asarray(g3b_stress0)
a = x.dot(y)/x.dot(x)   # slope of least squares fit line through origin, y = a.x
r_sq = a**2 * x.dot(x)/y.dot(y)
print('G3b:', a, r_sq)
# G3c
x = np.asarray(g3c_strain0)
y = np.asarray(g3c_stress0)
a = x.dot(y)/x.dot(x)   # slope of least squares fit line through origin, y = a.x
r_sq = a**2 * x.dot(x)/y.dot(y)
print('G3c:', a, r_sq)
# G5b
x = np.asarray(g5b_strain0)
y = np.asarray(g5b_stress0)
a = x.dot(y)/x.dot(x)   # slope of least squares fit line through origin, y = a.x
r_sq = a**2 * x.dot(x)/y.dot(y)
print('G5b:', a, r_sq)
# G5c
x = np.asarray(g5c_strain0[:-1])
y = np.asarray(g5c_stress0[:-1])
a = x.dot(y)/x.dot(x)   # slope of least squares fit line through origin, y = a.x
r_sq = a**2 * x.dot(x)/y.dot(y)
print('G5c:', a, r_sq)
# full set
x = np.concatenate([g2b_strain0, g3b_strain0, g3c_strain0, g5b_strain0, g5c_strain0[:-1]])
y = np.concatenate([g2b_stress0, g3b_stress0, g3c_stress0, g5b_stress0, g5c_stress0[:-1]])
a = x.dot(y)/x.dot(x)   # slope of least squares fit line through origin, y = a.x
r_sq = a**2 * x.dot(x)/y.dot(y)
print('All:', a, r_sq)
```

```
G2b: 2226.58091848 0.999778522623
G3b: 2009.51167966 0.999221188712
G3c: 1778.88745462 0.999946259334
G5b: 1880.94290498 0.999994804567
G5c: 1722.31071987 0.999535252518
All: 1946.2922994 0.991920901787
```

## Young's modulus vs. stress¶

In [7]:

```
fig=plt.figure(figsize=(12,3.9))
axis_limits = [0, 270, 0, 17.5]
x_tick_spec = np.arange(0, 300, 50)
y_tick_spec = np.arange(0, 18, 2)
#
# Ground nylon
ax1=fig.add_subplot(131)
# Es
ax1.plot(n8_Es_stress, n8_Es, color=n8_pen, ls='-', lw=1, marker='*', mec=n8_pen, mfc='none', mew=1)
ax1.plot(n14_Es_stress, n14_Es, color=n14_pen, ls='-', lw=1, marker='*', mec=n14_pen, mfc='none', mew=1)
ax1.plot(n5_Es_stress, n5_Es, color=n5_pen, ls='-', lw=1, marker='*', mec=n5_pen, mfc='none', mew=1)
ax1.plot(n23a_Es_stress, n23a_Es, color=n23a_pen, ls='-', lw=1, marker='*', mec=n23a_pen, mfc='none', mew=1)
ax1.plot(n29_Es_stress, n29_Es, color=n29_pen, ls='-', lw=1, marker='*', mec=n29_pen, mfc='none', mew=1)
# Et
ax1.plot(n8_stress, n8_Et, color=n8_pen, ls='-', lw=1, marker='+', mec=n8_pen, mfc='none', mew=1)
ax1.plot(n14_stress, n14_Et, color=n14_pen, ls='-', lw=1, marker='+', mec=n14_pen, mfc='none', mew=1)
ax1.plot(n5_stress, n5_Et, color=n5_pen, ls='-', lw=1, marker='+', mec=n5_pen, mfc='none', mew=1)
ax1.plot(n23a_stress, n23a_Et, color=n23a_pen, ls='-', lw=1, marker='+', mec=n23a_pen, mfc='none', mew=1)
ax1.plot(n29_stress, n29_Et, color=n29_pen, ls='-', lw=1, marker='+', mec=n29_pen, mfc='none', mew=1)
# Eb
ax1.plot(n8_stress, n8_Eb, color=n8_pen, ls='-', lw=1, marker='o', ms=4, mec=n8_pen, mfc='none', mew=1)
ax1.plot(n14_stress, n14_Eb, color=n14_pen, ls='-', lw=1, marker='o', ms=4, mec=n14_pen, mfc='none', mew=1)
ax1.plot(n5_stress, n5_Eb, color=n5_pen, ls='-', lw=1, marker='o', ms=4, mec=n5_pen, mfc='none', mew=1)
ax1.plot(n23a_stress, n23a_Eb, color=n23a_pen, ls='-', lw=1, marker='o', ms=4, mec=n23a_pen, mfc='none', mew=1)
ax1.plot(n29_stress, n29_Eb, color=n29_pen, ls='-', lw=1, marker='o', ms=4, mec=n29_pen, mfc='none', mew=1)
# plain lines for legend
ax1.plot([-2,-1], [-2,-1], color=n8_pen, ls='-', lw=1, label=n8_leg)
ax1.plot([-2,-1], [-2,-1], color=n14_pen, ls='-', lw=1, label=n14_leg)
ax1.plot([-2,-1], [-2,-1], color=n5_pen, ls='-', lw=1, label=n5_leg)
ax1.plot([-2,-1], [-2,-1], color=n23a_pen, ls='-', lw=1, label=n23a_leg)
ax1.plot([-2,-1], [-2,-1], color=n29_pen, ls='-', lw=1, label=n29_leg)
ax1.legend(loc='best', ncol = 1, prop={'size':8})
ax1.axis(axis_limits)
ax1.set_xticks(x_tick_spec)
ax1.set_yticks(y_tick_spec)
ax1.set_xlabel('Stress (MPa)')
ax1.set_ylabel("Young's modulus (GPa)")
#
# Carbon
ax2=fig.add_subplot(132)
# Es
ax2.plot(c1_Es_stress, c1_Es, color=c1_pen, ls='-', lw=1, marker='*', mec=c1_pen, mfc='none', mew=1)
ax2.plot(c3_Es_stress, c3_Es, color=c3_pen, ls='-', lw=1, marker='*', mec=c3_pen, mfc='none', mew=1)
ax2.plot(c5b_Es_stress, c5b_Es, color=c5b_pen, ls='-', lw=1, marker='*', mec=c5b_pen, mfc='none', mew=1)
ax2.plot(c7a_Es_stress, c7a_Es, color=c7a_pen, ls='-', lw=1, marker='*', mec=c7a_pen, mfc='none', mew=1)
# Et
ax2.plot(c1_stress, c1_Et, color=c1_pen, ls='-', lw=1, marker='+', mec=c1_pen, mfc='none', mew=1)
ax2.plot(c3_stress, c3_Et, color=c3_pen, ls='-', lw=1, marker='+', mec=c3_pen, mfc='none', mew=1)
ax2.plot(c5b_stress, c5b_Et, color=c5b_pen, ls='-', lw=1, marker='+', mec=c5b_pen, mfc='none', mew=1)
ax2.plot(c7a_stress, c7a_Et, color=c7a_pen, ls='-', lw=1, marker='+', mec=c7a_pen, mfc='none', mew=1)
# Eb
ax2.plot(c1_stress, c1_Eb, color=c1_pen, ls='-', lw=1, marker='o', ms=4, mec=c1_pen, mfc='none', mew=1)
ax2.plot(c3_stress, c3_Eb, color=c3_pen, ls='-', lw=1, marker='o', ms=4, mec=c3_pen, mfc='none', mew=1)
ax2.plot(c5b_stress, c5b_Eb, color=c5b_pen, ls='-', lw=1, marker='o', ms=4, mec=c5b_pen, mfc='none', mew=1)
ax2.plot(c7a_stress, c7a_Eb, color=c7a_pen, ls='-', lw=1, marker='o', ms=4, mec=c7a_pen, mfc='none', mew=1)
# plain lines for legend
ax2.plot([-2,-1], [-2,-1], color=c1_pen, ls='-', lw=1, label=c1_leg)
ax2.plot([-2,-1], [-2,-1], color=c3_pen, ls='-', lw=1, label=c3_leg)
ax2.plot([-2,-1], [-2,-1], color=c5b_pen, ls='-', lw=1, label=c5b_leg)
ax2.plot([-2,-1], [-2,-1], color=c7a_pen, ls='-', lw=1, label=c7a_leg)
ax2.legend(loc='best', ncol = 1, prop={'size':8})
ax2.axis(axis_limits)
ax2.set_xticks(x_tick_spec)
ax2.set_yticks(y_tick_spec)
ax2.set_xlabel('Stress (MPa)')
ax2.set_ylabel("Young's modulus (GPa)")
#
# Gut
ax3=fig.add_subplot(133)
# Es
ax3.plot(g2b_Es_stress, g2b_Es, color=g2b_pen, ls='-', lw=1, marker='*', mec=g2b_pen, mfc='none', mew=1)
ax3.plot(g3b_Es_stress, g3b_Es, color=g3b_pen, ls='-', lw=1, marker='*', mec=g3b_pen, mfc='none', mew=1)
ax3.plot(g3c_Es_stress, g3c_Es, color=g3c_pen, ls='-', lw=1, marker='*', mec=g3c_pen, mfc='none', mew=1)
ax3.plot(g5b_Es_stress, g5b_Es, color=g5b_pen, ls='-', lw=1, marker='*', mec=g5b_pen, mfc='none', mew=1)
ax3.plot(g5c_Es_stress[:-1], g5c_Es[:-1], color=g5c_pen, ls='-', lw=1, marker='*', mec=g5c_pen, mfc='none', mew=1)
# Et
ax3.plot(g2b_stress, g2b_Et, color=g2b_pen, ls='-', lw=1, marker='+', mec=g2b_pen, mfc='none', mew=1)
ax3.plot(g3b_stress, g3b_Et, color=g3b_pen, ls='-', lw=1, marker='+', mec=g3b_pen, mfc='none', mew=1)
ax3.plot(g3c_stress, g3c_Et, color=g3c_pen, ls='-', lw=1, marker='+', mec=g3c_pen, mfc='none', mew=1)
ax3.plot(g5b_stress, g5b_Et, color=g5b_pen, ls='-', lw=1, marker='+', mec=g5b_pen, mfc='none', mew=1)
ax3.plot(g5c_stress[:-1], g5c_Et[:-1], color=g5c_pen, ls='-', lw=1, marker='+', mec=g5c_pen, mfc='none', mew=1)
# Eb from manual plucks
ax3.plot(g2b_stress, g2b_Eb_man, color=g2b_pen, ls='-', lw=1, marker='o', ms=4, mec=g2b_pen, mfc='none', mew=1)
ax3.plot(g3b_stress, g3b_Eb_man, color=g3b_pen, ls='-', lw=1, marker='o', ms=4, mec=g3b_pen, mfc='none', mew=1)
ax3.plot(g3c_stress, g3c_Eb_man, color=g3c_pen, ls='-', lw=1, marker='o', ms=4, mec=g3c_pen, mfc='none', mew=1)
ax3.plot(g5b_stress, g5b_Eb_man, color=g5b_pen, ls='-', lw=1, marker='o', ms=4, mec=g5b_pen, mfc='none', mew=1)
ax3.plot(g5c_stress[:-1], g5c_Eb_man[:-1], color=g5c_pen, ls='-', lw=1, marker='o', ms=4, mec=g5c_pen, mfc='none', mew=1)
# plain lines for legend
ax3.plot([-2,-1], [-2,-1], color=g2b_pen, ls='-', lw=1, label=g2b_leg)
ax3.plot([-2,-1], [-2,-1], color=g3b_pen, ls='-', lw=1, label=g3_leg)
ax3.plot([-2,-1], [-2,-1], color=g5b_pen, ls='-', lw=1, label=g5_leg)
ax3.legend(loc='upper left', ncol = 1, prop={'size':8})
ax3.axis(axis_limits)
ax3.set_xticks(x_tick_spec)
ax3.set_yticks(y_tick_spec)
ax3.set_xlabel('Stress (MPa)')
ax3.set_ylabel("Young's modulus (GPa)")
#
plt.tight_layout()
ax1.text(0.87, 0.93,'(a)', transform=ax1.transAxes)
ax2.text(0.87, 0.93,'(b)', transform=ax2.transAxes)
ax3.text(0.87, 0.93,'(c)', transform=ax3.transAxes)
plt.savefig('./02abc_youngs_modulus.eps', format='eps', dpi=1000)
plt.show()
```

## Function fits for Young's modulus¶

In [8]:

```
def Et_grid_value(stress, density):
    # Et function (GPa) for ground nylon is 32.0 + 0.0353 * notional stress (MPa) - 0.0269 * initial density (kg/m**3).
    coeff_a = 31.9859792151428
    coeff_b = 0.0352816913659568
    coeff_c = -0.0269321235467744
    return (coeff_a + coeff_b * stress + coeff_c * density)

# ground nylon
print('ground nylon')
args = (n8_stress_n, n14_stress_n, n5_stress_n, n23a_stress_n, n29_stress_n, n10b_stress_n)  
n_stress_values = np.concatenate(args)  
args = (n8_rho_n, n14_rho_n, n5_rho_n, n23a_rho_n, n29_rho_n, n10b_rho_n)
n_density_values = np.concatenate(args) 
args = (n8_Et, n14_Et, n5_Et, n23a_Et, n29_Et, n10b_Et)
n_Et_values = np.concatenate(args) 
args = (n8_Eb, n14_Eb, n5_Eb, n23a_Eb, n29_Eb, n10b_Eb)
n_Eb_values = np.concatenate(args) 
# Eb
fit = np.polyfit(n_stress_values, n_Eb_values, 1)
fit_fn = np.poly1d(fit)
n_Eb_est = fit_fn(n_stress_values)
n_Eb_rsq = r_squared(n_Eb_values, n_Eb_est)
print('Eb: fit fn:', fit, 'rsq:', n_Eb_rsq)
# Et line
fit = np.polyfit(n_stress_values, n_Et_values, 1)
fit_fn = np.poly1d(fit)
n_Et_est = fit_fn(n_stress_values)
n_Et_rsq = r_squared(n_Et_values, n_Et_est)
print('Et: fit fn:', fit, 'rsq:', n_Et_rsq)
# Et grid
n_Et_grid_est = Et_grid_value(n_stress_values, n_density_values)
n_Et_grid_rsq = r_squared(n_Et_values, n_Et_grid_est)
print('Et grid: rsq:', n_Et_grid_rsq)

# carbon
print('\ncarbon')
args = (c1_stress_n, c3_stress_n, c5b_stress_n, c7a_stress_n)  
c_stress_values = np.concatenate(args)  
args = (c1_rho_n, c3_rho_n, c5b_rho_n, c7a_rho_n)
c_density_values = np.concatenate(args) 
args = (c1_Et, c3_Et, c5b_Et, c7a_Et)
c_Et_values = np.concatenate(args) 
args = (c1_Eb, c3_Eb, c5b_Eb, c7a_Eb)
c_Eb_values = np.concatenate(args) 
# Eb
fit = np.polyfit(c_stress_values, c_Eb_values, 1)
fit_fn = np.poly1d(fit)
c_Eb_est = fit_fn(c_stress_values)
c_Eb_rsq = r_squared(c_Eb_values, c_Eb_est)
print('Eb: fit fn:', fit, 'rsq:', c_Eb_rsq)
# Et line
fit = np.polyfit(c_stress_values, c_Et_values, 1)
fit_fn = np.poly1d(fit)
c_Et_est = fit_fn(c_stress_values)
c_Et_rsq = r_squared(c_Et_values, c_Et_est)
print('Et: fit fn:', fit, 'rsq:', c_Et_rsq)
```

```
ground nylon
Eb: fit fn: [ 0.03887992  4.52905496] rsq: 0.956676977356
Et: fit fn: [ 0.039818    1.73014418] rsq: 0.857705644257
Et grid: rsq: 0.98316162931

carbon
Eb: fit fn: [ 0.04100955  3.24746902] rsq: 0.813913072698
Et: fit fn: [ 0.03601406  1.39146228] rsq: 0.947796334416
```

## $E\_T$ comparison with Bell & Firth¶

In [9]:

```
# plotting averages across all test frequencies
plt.figure(figsize=(6,4.5))
# Es
# fitted line
fit = np.polyfit(gop_inv_d, avg_Es, 1)
print(fit)
fit_fn = np.poly1d(fit)
plt.plot([0.0,2.0], [fit_fn(0),fit_fn(2.0)], 'k--')
# data
plt.plot(gop_inv_d, avg_Es, 'k*', ms=8, mfc='none', mew=1.5, label='$E_S$ from stress vs. strain')
# Et
# fitted line
fit = np.polyfit(gop_inv_d, avg_Et, 1)
print(fit)
fit_fn = np.poly1d(fit)
plt.plot([0.0,2.0], [fit_fn(0),fit_fn(2.0)], 'k--')
# data
plt.plot(gop_inv_d, avg_Et, 'k+', ms=8, mfc='none', mew=1.5, label='$E_T$ from tension modulation')
# Eb
# fitted line
fit = np.polyfit(gop_inv_d, avg_Eb_man, 1)
print(fit)
fit_fn = np.poly1d(fit)
plt.plot([0.0,2.0], [fit_fn(0),fit_fn(2.0)], 'k--')
# data
plt.plot(gop_inv_d, avg_Eb_man, 'ko', ms=6, mfc='none', mew=1.5, label='$E_B$ from bending stiffness')
# Bell & Firth
# fitted line (weighted by sample sizes)
fit = np.polyfit(bf_inv_d, bf_Et, 1, w=bf_weights)
fit_fn = np.poly1d(fit)
plt.plot([0.0,2.0], [fit_fn(0),fit_fn(2.0)], 'magenta', ls='--')
# data
plt.errorbar(bf_inv_d, bf_Et, yerr=bf_Et_SD, ls='none', marker='o', ms=6, mec='magenta', mfc='magenta', ecolor='magenta', capsize=3, label='Data from Bell and Firth')
# axes
plt.legend(loc='lower center', ncol = 2)
#plt.legend(loc='lower right', ncol = 2, prop={'size':8})
plt.axis([0, 2, 0, 7])
plt.xlabel('1 / unstretched diameter (1/mm)')
plt.ylabel("Young's modulus (GPa)")
plt.tight_layout()  
plt.savefig('./03_bell_firth_avg.eps', format='eps', dpi=1000)
plt.show()
```

```
[ 0.77078119  1.30928227]
[ 1.68482772  2.90517476]
[ 2.67122351  3.66336238]
```

## $dE/dT$ vs. stress¶

In [10]:

```
# plot against stress
fig=plt.figure(figsize=(12,3.9))
axis_limits = [0, 270, -0.14, 0.005]
x_tick_spec = np.arange(0, 300, 50)
y_tick_spec = np.arange(-0.14, 0.02, 0.02)
#
# Ground nylon
ax1=fig.add_subplot(131)
# dEb/dT
ax1.plot(n14_stress, n14_dEbdT, color=n14_pen, ls='-', lw=1, marker='o', ms=4, mec=n14_pen, mfc='none', mew=1)
ax1.plot(n5_stress, n5_dEbdT, color=n5_pen, ls='-', lw=1, marker='o', ms=4, mec=n5_pen, mfc='none', mew=1)
ax1.plot(n29_stress, n29_dEbdT, color=n29_pen, ls='-', lw=1, marker='o', ms=4, mec=n29_pen, mfc='none', mew=1)
# dEt/dT
ax1.plot(n8_stress, n8_dEtdT, color=n8_pen, ls='-', lw=1, marker='+', mec=n8_pen, mfc=n8_pen, mew=1)
ax1.plot(n14_stress, n14_dEtdT, color=n14_pen, ls='-', lw=1, marker='+', mec=n14_pen, mfc=n14_pen, mew=1)
ax1.plot(n5_stress, n5_dEtdT, color=n5_pen, ls='-', lw=1, marker='+', mec=n5_pen, mfc=n5_pen, mew=1)
ax1.plot(n23a_stress, n23a_dEtdT, color=n23a_pen, ls='-', lw=1, marker='+', mec=n23a_pen, mfc=n23a_pen, mew=1)
ax1.plot(n29_stress, n29_dEtdT, color=n29_pen, ls='-', lw=1, marker='+', mec=n29_pen, mfc=n29_pen, mew=1)
# plain lines for legend
ax1.plot([-2,-1], [-2,-1], color=n8_pen, ls='-', lw=1, label=n8_leg)
ax1.plot([-2,-1], [-2,-1], color=n14_pen, ls='-', lw=1, label=n14_leg)
ax1.plot([-2,-1], [-2,-1], color=n5_pen, ls='-', lw=1, label=n5_leg)
ax1.plot([-2,-1], [-2,-1], color=n23a_pen, ls='-', lw=1, label=n23a_leg)
ax1.plot([-2,-1], [-2,-1], color=n29_pen, ls='-', lw=1, label=n29_leg)
ax1.legend(loc='lower right', ncol = 1, prop={'size':8})
ax1.axis(axis_limits)
ax1.set_xticks(x_tick_spec)
ax1.set_yticks(y_tick_spec)
ax1.set_xlabel('Stress (MPa)')
ax1.set_ylabel('$dE/dT$  (GPa/$^\circ$C)')
#
# Carbon
ax2=fig.add_subplot(132)
# dEb/dT
ax2.plot(c1_stress, c1_dEbdT, color=c1_pen, ls='-', lw=1, marker='o', ms=4, mec=c1_pen, mfc='none', mew=1)
ax2.plot(c3_stress, c3_dEbdT, color=c3_pen, ls='-', lw=1, marker='o', ms=4, mec=c3_pen, mfc='none', mew=1)
ax2.plot(c5b_stress, c5b_dEbdT, color=c5b_pen, ls='-', lw=1, marker='o', ms=4, mec=c5b_pen, mfc='none', mew=1)
ax2.plot(c7a_stress, c7a_dEbdT, color=c7a_pen, ls='-', lw=1, marker='o', ms=4, mec=c7a_pen, mfc='none', mew=1)
# dEt/dT
ax2.plot(c1_stress, c1_dEtdT, color=c1_pen, ls='-', lw=1, marker='+', mec=c1_pen, mfc=c1_pen, mew=1)
ax2.plot(c3_stress, c3_dEtdT, color=c3_pen, ls='-', lw=1, marker='+', mec=c3_pen, mfc=c3_pen, mew=1)
ax2.plot(c5b_stress, c5b_dEtdT, color=c5b_pen, ls='-', lw=1, marker='+', mec=c5b_pen, mfc=c5b_pen, mew=1)
ax2.plot(c7a_stress, c7a_dEtdT, color=c7a_pen, ls='-', lw=1, marker='+', mec=c7a_pen, mfc=c7a_pen, mew=1)
# plain lines for legend
ax2.plot([-2,-1], [-2,-1], color=c1_pen, ls='-', lw=1, label=c1_leg)
ax2.plot([-2,-1], [-2,-1], color=c3_pen, ls='-', lw=1, label=c3_leg)
ax2.plot([-2,-1], [-2,-1], color=c5b_pen, ls='-', lw=1, label=c5b_leg)
ax2.plot([-2,-1], [-2,-1], color=c7a_pen, ls='-', lw=1, label=c7a_leg)
ax2.legend(loc='lower left', ncol = 1, prop={'size':8})
ax2.axis(axis_limits)
ax2.set_xticks(x_tick_spec)
ax2.set_yticks(y_tick_spec)
ax2.set_xlabel('Stress (MPa)')
ax2.set_ylabel('$dE/dT$  (GPa/$^\circ$C)')
#
# Gut
ax3=fig.add_subplot(133)
# dEt/dT
ax3.plot(g2b_stress, g2b_dEtdT, color=g2b_pen, ls='-', lw=1, marker='+', mec=g2b_pen, mfc=g2b_pen, mew=1)
ax3.plot(g3b_stress, g3b_dEtdT, color=g3b_pen, ls='-', lw=1, marker='+', mec=g3b_pen, mfc=g3b_pen, mew=1)
ax3.plot(g3c_stress, g3c_dEtdT, color=g3c_pen, ls='-', lw=1, marker='+', mec=g3c_pen, mfc=g3c_pen, mew=1)
ax3.plot(g5b_stress, g5b_dEtdT, color=g5b_pen, ls='-', lw=1, marker='+', mec=g5b_pen, mfc=g5b_pen, mew=1)
ax3.plot(g5c_stress[:-1], g5c_dEtdT[:-1], color=g5c_pen, ls='-', lw=1, marker='+', mec=g5c_pen, mfc=g5c_pen, mew=1)
# plain lines for legend
ax3.plot([-2,-1], [-2,-1], color=g2b_pen, ls='-', lw=1, label=g2b_leg)
ax3.plot([-2,-1], [-2,-1], color=g3b_pen, ls='-', lw=1, label=g3_leg)
ax3.plot([-2,-1], [-2,-1], color=g5b_pen, ls='-', lw=1, label=g5_leg)
ax3.legend(loc='lower right', ncol = 1, prop={'size':8})
ax3.axis(axis_limits)
ax3.set_xticks(x_tick_spec)
ax3.set_yticks(y_tick_spec)
ax3.set_xlabel('Stress (MPa)')
ax3.set_ylabel('$dE/dT$  (GPa/$^\circ$C)')
#
plt.tight_layout()
ax1.text(0.05, 0.9,'(a)', transform=ax1.transAxes)
ax2.text(0.05, 0.9,'(b)', transform=ax2.transAxes)
ax3.text(0.05, 0.9,'(c)', transform=ax3.transAxes)
plt.savefig('./04abc_dEdT.eps', format='eps', dpi=1000)
plt.show()
```

## Density ratio vs. strain¶

In [11]:

```
fig=plt.figure(figsize=(12,3.9))
axis_limits = [0, 0.2, 0.92, 1.04]
x_tick_spec = np.arange(0, 0.25, 0.05)
y_tick_spec = np.arange(0.92, 1.05, 0.02)
# Ground nylon
ax1=fig.add_subplot(131)
dash_range = 0.08
ax1.plot([0,dash_range],[1,1-(0.3*dash_range)], 'k--', lw=1.5)
ax1.errorbar(x=n8_strain0, y=n8_den_ratio, yerr=[n8_den_err_minus, n8_den_err_plus], color=n8_pen, lw=1, marker='o', ms=3, mec=n8_pen, mfc=n8_pen, mew=1, label=n8_leg, elinewidth=1, capsize=3)
ax1.errorbar(x=n14_strain0, y=n14_den_ratio, yerr=[n14_den_err_minus, n14_den_err_plus], color=n14_pen, lw=1, marker='o', ms=3, mec=n14_pen, mfc=n14_pen, mew=1, label=n14_leg, elinewidth=1, capsize=3)
ax1.errorbar(x=n5_strain0, y=n5_den_ratio, yerr=[n5_den_err_minus, n5_den_err_plus], color=n5_pen, lw=1, marker='o', ms=3, mec=n5_pen, mfc=n5_pen, mew=1, label=n5_leg, elinewidth=1, capsize=3)
ax1.errorbar(x=n23a_strain0, y=n23a_den_ratio, yerr=[n23a_den_err_minus, n23a_den_err_plus], color=n23a_pen, lw=1, marker='o', ms=3, mec=n23a_pen, mfc=n23a_pen, mew=1, label=n23a_leg, elinewidth=1, capsize=3)
#ax1.errorbar(x=n29_strain0, y=n29_den_ratio, yerr=[n29_den_err_minus, n29_den_err_plus], color=n29_pen, lw=1, marker='o', ms=3, mec=n29_pen, mfc=n29_pen, mew=1, label=n29_leg, elinewidth=1, capsize=3)
ax1.legend(loc='lower center', ncol = 2, prop={'size':8})
ax1.axis(axis_limits)
ax1.set_xticks(x_tick_spec)
ax1.set_yticks(y_tick_spec)
ax1.set_xlabel('Strain')
ax1.set_ylabel('Density ratio')
# Carbon
ax2=fig.add_subplot(132)
dash_range = 0.07
ax2.plot([0,dash_range],[1,1-(0.3*dash_range)], 'k--', lw=1.5)
ax2.errorbar(x=c1_strain0, y=c1_den_ratio, yerr=[c1_den_err_minus, c1_den_err_plus], color=c1_pen, lw=1, marker='o', ms=3, mec=c1_pen, mfc=c1_pen, mew=1, label=c1_leg, elinewidth=1, capsize=3)
ax2.errorbar(x=c3_strain0, y=c3_den_ratio, yerr=[c3_den_err_minus, c3_den_err_plus], color=c3_pen, lw=1, marker='o', ms=3, mec=c3_pen, mfc=c3_pen, mew=1, label=c3_leg, elinewidth=1, capsize=3)
ax2.errorbar(x=c5b_strain0, y=c5b_den_ratio, yerr=[c5b_den_err_minus, c5b_den_err_plus], color=c5b_pen, lw=1, marker='o', ms=3, mec=c5b_pen, mfc=c5b_pen, mew=1, label=c5b_leg, elinewidth=1, capsize=3)
ax2.errorbar(x=c7a_strain0, y=c7a_den_ratio, yerr=[c7a_den_err_minus, c7a_den_err_plus], color=c7a_pen, lw=1, marker='o', ms=3, mec=c7a_pen, mfc=c7a_pen, mew=1, label=c7a_leg, elinewidth=1, capsize=3)
ax2.legend(loc='lower center', ncol = 2, prop={'size':8})
ax2.axis(axis_limits)
ax2.set_xticks(x_tick_spec)
ax2.set_yticks(y_tick_spec)
ax2.set_xlabel('Strain')
ax2.set_ylabel('Density ratio')
# Gut
ax3=fig.add_subplot(133)
dash_range = 0.08
ax3.errorbar(x=g2b_strain0, y=g2b_den_ratio, yerr=[g2b_den_err_minus, g2b_den_err_plus], color=g2b_pen, ls='--', lw=1, marker='o', ms=3, mec=g2b_pen, mfc=g2b_pen, mew=1, label=g2b_leg, elinewidth=1, capsize=3)
ax3.errorbar(x=g3b_strain0, y=g3b_den_ratio, yerr=[g3b_den_err_minus, g3b_den_err_plus], color=g3b_pen, ls='--', lw=1, marker='o', ms=3, mec=g3b_pen, mfc=g3b_pen, mew=1, label=g3_leg, elinewidth=1, capsize=3)
ax3.errorbar(x=g3c_strain0, y=g3c_den_ratio, yerr=[g3c_den_err_minus, g3c_den_err_plus], color=g3c_pen, ls='--', lw=1, marker='o', ms=3, mec=g3c_pen, mfc=g3c_pen, mew=1, elinewidth=1, capsize=3)
ax3.errorbar(x=g5b_strain0, y=g5b_den_ratio, yerr=[g5b_den_err_minus, g5b_den_err_plus], color=g5b_pen, ls='--', lw=1, marker='o', ms=3, mec=g5b_pen, mfc=g5b_pen, mew=1, label=g5_leg, elinewidth=1, capsize=3)
ax3.errorbar(x=g5c_strain0[:-1], y=g5c_den_ratio[:-1], yerr=[g5c_den_err_minus[:-1], g5c_den_err_plus[:-1]], color=g5c_pen, ls='--', lw=1, marker='o', ms=3, mec=g5c_pen, mfc=g5c_pen, mew=1, elinewidth=1, capsize=3)
ax3.errorbar(x=[g5c_strain0[-3],g5c_strain0[-1]], y=[g5c_den_ratio[-3],g5c_den_ratio[-1]], yerr=[[g5c_den_err_minus[-3],g5c_den_err_minus[-1]], [g5c_den_err_plus[-3],g5c_den_err_plus[-1]]], color=g5c_pen, ls=':', lw=1, marker='o', ms=3, mec=g5c_pen, mfc=g5c_pen, mew=1, elinewidth=1, capsize=3)
ax3.legend(loc='lower right', ncol = 1, prop={'size':8})
ax3.axis(axis_limits)
ax3.set_xticks(x_tick_spec)
ax3.set_yticks(y_tick_spec)
ax3.set_xlabel('Strain')
ax3.set_ylabel('Density ratio')
#
plt.tight_layout()
ax1.text(0.87, 0.9,'(a)', transform=ax1.transAxes)
ax2.text(0.87, 0.9,'(b)', transform=ax2.transAxes)
ax3.text(0.87, 0.9,'(c)', transform=ax3.transAxes)
plt.savefig('./05abc_density.eps', format='eps', dpi=1000)
plt.show()
```

## Working tension vs. notional tension¶

In [12]:

```
# Notes on regression through the origin
# see: https://online.stat.psu.edu/~ajw13/stat501/SpecialTopics/Reg_thru_origin.pdf
# Joseph G. Eisenhauer. Regression through the Origin. Teaching Statistics. Volume 25, Number 3, pp. 76-80, Autumn 2003
# 
# slope of least squares fit line through origin, y_est = a.x
# a = sum(x.y)/sum(x**2) = x.dot(y)/x.dot(x)
#
# for the coefficient of determination for regression through the origin:
# r^2 = sum(y_est**2)/sum(y**2) = y_est.dot(y_est)/y.dot(y) = a**2 * x.dot(x)/y.dot(y)

fig=plt.figure(figsize=(12,3.9))
axis_limits = [0, 350, 0, 350]
x_tick_spec = np.arange(0, 400, 50)
y_tick_spec = np.arange(0, 400, 50)
#
# Ground nylon
ax1=fig.add_subplot(131)
# fitted line 
x = np.asarray(nop_Fn_full)
y = np.asarray(nop_Fw_full)
a = x.dot(y)/x.dot(x)   # slope of least squares fit line through origin, y = a.x
r_sq = a**2 * x.dot(x)/y.dot(y)
print(a, r_sq)
ax1.plot([0,350], [0,350*a], 'k--', lw=1)
# data
ax1.plot(n8_Fn, n8_Fw, color=n8_pen, lw=1, label=n8_leg)
ax1.plot(n14_Fn, n14_Fw, color=n14_pen, lw=1, label=n14_leg)
ax1.plot(n5_Fn, n5_Fw, color=n5_pen, lw=1, label=n5_leg)
ax1.plot(n23a_Fn, n23a_Fw, color=n23a_pen, lw=1, label=n23a_leg)
ax1.plot(n29_Fn, n29_Fw, color=n29_pen, lw=1, label=n29_leg)
ax1.plot(nop_Fn, nop_Fw, color=op_pen, ls='none', marker=op_marker, ms=op_ms, mec=op_pen, mfc=op_mfc, mew=op_mew, label=op_leg)
ax1.legend(loc='best', ncol = 1, prop={'size':8})
ax1.axis(axis_limits)
ax1.set_xticks(x_tick_spec)
ax1.set_yticks(y_tick_spec)
ax1.set_xlabel('Notional tension (N)')
ax1.set_ylabel('Actual tension (N)')
#
# Carbon
ax2=fig.add_subplot(132)
# fitted line 
x = np.asarray(cop_Fn)
y = np.asarray(cop_Fw)
a = x.dot(y)/x.dot(x)   # slope of least squares fit line through origin, y = a.x
r_sq = a**2 * x.dot(x)/y.dot(y)
print(a, r_sq)
ax2.plot([0,350], [0,350*a], 'k--', lw=1)
# data
ax2.plot(c1_Fn, c1_Fw, color=c1_pen, lw=1, label=c1_leg)
ax2.plot(c3_Fn, c3_Fw, color=c3_pen, lw=1, label=c3_leg)
ax2.plot(c5b_Fn, c5b_Fw, color=c5b_pen, lw=1, label=c5b_leg)
ax2.plot(c7a_Fn, c7a_Fw, color=c7a_pen, lw=1, label=c7a_leg)
ax2.plot(cop_Fn, cop_Fw, color=op_pen, ls='none', marker=op_marker, ms=op_ms, mec=op_pen, mfc=op_mfc, mew=op_mew, label=op_leg)
ax2.legend(loc='best', ncol = 1, prop={'size':8})
ax2.axis(axis_limits)
ax2.set_xticks(x_tick_spec)
ax2.set_yticks(y_tick_spec)
ax2.set_xlabel('Notional tension (N)')
ax2.set_ylabel('Actual tension (N)')
#
# Gut
ax3=fig.add_subplot(133)
# fitted line 
x = np.asarray(gop_Fn)
y = np.asarray(gop_Fw)
a = x.dot(y)/x.dot(x)   # slope of least squares fit line through origin, y = a.x
r_sq = a**2 * x.dot(x)/y.dot(y)
print(a, r_sq)
ax3.plot([0,350], [0,350*a], 'k--', lw=1)
# data
ax3.plot(g2b_Fn, g2b_Fw, color=g2b_pen, lw=1, label=g2b_leg)
ax3.plot(g3b_Fn, g3b_Fw, color=g3b_pen, lw=1, label=g3_leg)
ax3.plot(g3c_Fn, g3c_Fw, color=g3c_pen, lw=1)
ax3.plot(g5b_Fn, g5b_Fw, color=g5b_pen, lw=1, label=g5_leg)
ax3.plot(g5c_Fn[:-1], g5c_Fw[:-1], color=g5c_pen, lw=1)
ax3.plot(gop_Fn, gop_Fw, color=op_pen, ls='none', marker=op_marker, ms=op_ms, mec=op_pen, mfc=op_mfc, mew=op_mew, label=op_leg)
ax3.legend(loc='best', ncol = 1, prop={'size':8})
ax3.axis(axis_limits)
ax3.set_xticks(x_tick_spec)
ax3.set_yticks(y_tick_spec)
ax3.set_xlabel('Notional tension (N)')
ax3.set_ylabel('Actual tension (N)')
#
plt.tight_layout()
ax1.text(0.87, 0.9,'(a)', transform=ax1.transAxes)
ax2.text(0.87, 0.9,'(b)', transform=ax2.transAxes)
ax3.text(0.87, 0.9,'(c)', transform=ax3.transAxes)
plt.savefig('./06abc_working_tension.eps', format='eps', dpi=1000)
plt.show()
```

```
0.895676658861 0.999724206372
0.860280885975 0.999778578319
0.943360341024 0.999982519149
```

## $df/dT|\_L$ vs. stress¶

In [13]:

```
# estimated responses for nylon strings
stress_ratio = 0.97463729113807  # actual to notional stress ratio (from measured data)

def n_dfdT_L_fn_lo(diameter, act_stress, dfdT_est):
    # df/dT|L function (cent/deg.C) for lower density ground nylon is
    # 1.067 + 115 / notional stress (MPa) - 11.3 / notional tension (N)
    coeff_a = 1.06704323339857
    coeff_b = 115.157320571965
    coeff_c = -11.2992937536173
    for i in act_stress:
        # i is actual stress in MPa
        stress = i / stress_ratio  # notional stress in MPa
        tension = stress * np.pi * diameter**2 / 4.0  # notional tension in N
        dfdT_est.append(coeff_a + coeff_b / stress + coeff_c / tension)

def n_dfdT_L_fn_hi(diameter, act_stress, dfdT_est):
    # df/dT|L function (cent/deg.C) for higher density ground nylon is
    # 0.890 - 14.7 / notional stress (MPa) - 67.1 / notional tension (N)
    coeff_a = 0.890126279489474
    coeff_b = -14.6839943147396
    coeff_c = -67.1498180942761
    for i in act_stress:
        # i is actual stress in MPa
        stress = i / stress_ratio  # notional stress in MPa
        tension = stress * np.pi * diameter**2 / 4.0  # notional tension in N
        dfdT_est.append(coeff_a + coeff_b / stress + coeff_c / tension)

# plot against stress
fig=plt.figure(figsize=(12,3.9))
axis_limits = [0, 270, -4, 5]
x_tick_spec = np.arange(0, 300, 50)
y_tick_spec = np.arange(-4, 6, 1)
#
# Ground nylon
ax1=fig.add_subplot(131)
ax1.plot([0,300],[0,0],'k--',lw=1)  # Zero reference
arr_stress = np.arange(5, 300, 5)
# n8
# estimated response
n8_dfdT_L_est = []
n_dfdT_L_fn_lo(n8_dia_n, arr_stress, n8_dfdT_L_est)
ax1.plot(arr_stress, n8_dfdT_L_est, color=n8_pen, ls='--', lw=1)
# data
ax1.plot(n8_stress, n8_dfdT_L, color=n8_pen, ls='none', lw=1, marker='o', ms=3, mec=n8_pen, mfc=n8_pen, mew=1)
# n14
# estimated response
n14_dfdT_L_est = []
n_dfdT_L_fn_lo(n14_dia_n, arr_stress, n14_dfdT_L_est)
ax1.plot(arr_stress, n14_dfdT_L_est, color=n14_pen, ls='--', lw=1)
# data
ax1.plot(n14_stress, n14_dfdT_L, color=n14_pen, ls='none', lw=1, marker='o', ms=3, mec=n14_pen, mfc=n14_pen, mew=1)
# n5
# estimated response
n5_dfdT_L_est = []
n_dfdT_L_fn_lo(n5_dia_n, arr_stress, n5_dfdT_L_est)
ax1.plot(arr_stress, n5_dfdT_L_est, color=n5_pen, ls='--', lw=1)
# data
ax1.plot(n5_stress, n5_dfdT_L, color=n5_pen, ls='none', lw=1, marker='o', ms=3, mec=n5_pen, mfc=n5_pen, mew=1)
# n23a
# estimated response
n23a_dfdT_L_est = []
n_dfdT_L_fn_hi(n23a_dia_n, arr_stress, n23a_dfdT_L_est)
ax1.plot(arr_stress, n23a_dfdT_L_est, color=n23a_pen, ls='--', lw=1)
# data
ax1.plot(n23a_stress, n23a_dfdT_L, color=n23a_pen, ls='none', lw=1, marker='o', ms=3, mec=n23a_pen, mfc=n23a_pen, mew=1)
# n29
# estimated response
n29_dfdT_L_est = []
n_dfdT_L_fn_hi(n29_dia_n, arr_stress, n29_dfdT_L_est)
ax1.plot(arr_stress, n29_dfdT_L_est, color=n29_pen, ls='--', lw=1)
# data
ax1.plot(n29_stress, n29_dfdT_L, color=n29_pen, ls='none', lw=1, marker='o', ms=3, mec=n29_pen, mfc=n29_pen, mew=1)
# operating points
ax1.plot(nop_stress, nop_dfdT_L, color=op_pen, ls='none', marker=op_marker, ms=op_ms, mec=op_pen, mfc=op_mfc, mew=op_mew)
# check fit
# low density strings
dfdT_est = []
n_dfdT_L_fn_lo(n8_dia_n, n8_stress, dfdT_est)
n_dfdT_L_fn_lo(n14_dia_n, n14_stress, dfdT_est)
n_dfdT_L_fn_lo(n5_dia_n, n5_stress, dfdT_est)
dfdT_act = np.concatenate([n8_dfdT_L, n14_dfdT_L, n5_dfdT_L])
print('N8, N14, N5 rsq:', r_squared(dfdT_act, dfdT_est))
# high density strings
dfdT_est = []
n_dfdT_L_fn_hi(n23a_dia_n, n23a_stress, dfdT_est)
n_dfdT_L_fn_hi(n29_dia_n, n29_stress, dfdT_est)
dfdT_act = np.concatenate([n23a_dfdT_L, n29_dfdT_L])
print('N23a, N29 rsq:  ', r_squared(dfdT_act, dfdT_est))
# plain lines for legend
ax1.plot([-2,-1], [-2,-1], color=n8_pen, ls='-', lw=1, label=n8_leg)
ax1.plot([-2,-1], [-2,-1], color=n14_pen, ls='-', lw=1, label=n14_leg)
ax1.plot([-2,-1], [-2,-1], color=n5_pen, ls='-', lw=1, label=n5_leg)
ax1.plot([-2,-1], [-2,-1], color=n23a_pen, ls='-', lw=1, label=n23a_leg)
ax1.plot([-2,-1], [-2,-1], color=n29_pen, ls='-', lw=1, label=n29_leg)
ax1.plot([-20,-10], [-20,-10], color=op_pen, ls='none', marker=op_marker, ms=op_ms, mec=op_pen, mfc=op_mfc, mew=op_mew, label=op_leg)
ax1.legend(loc='lower right', ncol = 1, prop={'size':8})
ax1.axis(axis_limits)
ax1.set_xticks(x_tick_spec)
ax1.set_yticks(y_tick_spec)
ax1.set_xlabel('Stress (MPa)')
ax1.set_ylabel('Constant length $df/dT$  (cent/$^\circ$C)')
#
# Carbon
ax2=fig.add_subplot(132)
ax2.plot([0,300],[0,0],'k--',lw=1)  # Zero reference
x_plot = np.arange(5.0, 300.0, 5.0)
# C1
ax2.plot(c1_stress, c1_dfdT_L, color=c1_pen, ls='none', lw=1, marker='o', ms=3, mec=c1_pen, mfc=c1_pen, mew=1)
# fitted expression
x_data = 1.0 / np.asarray(c1_stress)
y_data = np.asarray(c1_dfdT_L)
fit = np.polyfit(x_data, y_data, 1)
c1_fit_fn = np.poly1d(fit)
ax2.plot(x_plot, c1_fit_fn(1/x_plot), color=c1_pen, ls='--', lw=1)
print('\nC1 fit:', fit, ' rsq:', r_squared(y_data, c1_fit_fn(x_data)))
# C3
ax2.plot(c3_stress, c3_dfdT_L, color=c3_pen, ls='none', lw=1, marker='o', ms=3, mec=c3_pen, mfc=c3_pen, mew=1)
# fitted expression
x_data = 1.0 / np.asarray(c3_stress)
y_data = np.asarray(c3_dfdT_L)
fit = np.polyfit(x_data, y_data, 1)
c3_fit_fn = np.poly1d(fit)
ax2.plot(x_plot, c3_fit_fn(1/x_plot), color=c3_pen, ls='--', lw=1)
print('C3 fit:', fit, ' rsq:', r_squared(y_data, c3_fit_fn(x_data)))
# C5b
ax2.plot(c5b_stress, c5b_dfdT_L, color=c5b_pen, ls='none', lw=1, marker='o', ms=3, mec=c5b_pen, mfc=c5b_pen, mew=1)
# fitted expression
x_data = 1.0 / np.asarray(c5b_stress)
y_data = np.asarray(c5b_dfdT_L)
fit = np.polyfit(x_data, y_data, 1)
c5b_fit_fn = np.poly1d(fit)
ax2.plot(x_plot, c5b_fit_fn(1/x_plot), color=c5b_pen, ls='--', lw=1)
print('C5b fit:', fit, ' rsq:', r_squared(y_data, c5b_fit_fn(x_data)))
# C7a
ax2.plot(c7a_stress, c7a_dfdT_L, color=c7a_pen, ls='none', lw=1, marker='o', ms=3, mec=c7a_pen, mfc=c7a_pen, mew=1)
# fitted expression
x_data = 1.0 / np.asarray(c7a_stress)
y_data = np.asarray(c7a_dfdT_L)
fit = np.polyfit(x_data, y_data, 1)
c7a_fit_fn = np.poly1d(fit)
ax2.plot(x_plot, c7a_fit_fn(1/x_plot), color=c7a_pen, ls='--', lw=1)
print('C7a fit:', fit, ' rsq:', r_squared(y_data, c7a_fit_fn(x_data)))
# operating points
ax2.plot(cop_stress, cop_dfdT_L, color=op_pen, ls='none', marker=op_marker, ms=op_ms, mec=op_pen, mfc=op_mfc, mew=op_mew)
# plain lines for legend
ax2.plot([-2,-1], [-2,-1], color=c1_pen, ls='-', lw=1, label=c1_leg)
ax2.plot([-2,-1], [-2,-1], color=c3_pen, ls='-', lw=1, label=c3_leg)
ax2.plot([-2,-1], [-2,-1], color=c5b_pen, ls='-', lw=1, label=c5b_leg)
ax2.plot([-2,-1], [-2,-1], color=c7a_pen, ls='-', lw=1, label=c7a_leg)
ax2.plot([-20,-10], [-20,-10], color=op_pen, ls='none', marker=op_marker, ms=op_ms, mec=op_pen, mfc=op_mfc, mew=op_mew, label=op_leg)
ax2.legend(loc='lower right', ncol = 1, prop={'size':8})
ax2.axis(axis_limits)
ax2.set_xticks(x_tick_spec)
ax2.set_yticks(y_tick_spec)
ax2.set_xlabel('Stress (MPa)')
ax2.set_ylabel('Constant length $df/dT$  (cent/$^\circ$C)')
#
# Gut
ax3=fig.add_subplot(133)
ax3.plot([0,300],[0,0],'k--',lw=1)  # Zero reference
x_plot = np.arange(5.0, 300.0, 5.0)
# G2b
ax3.plot(g2b_stress, g2b_dfdT_L, color=g2b_pen, ls='none', lw=1, marker='o', ms=3, mec=g2b_pen, mfc=g2b_pen, mew=1)
# fitted expression
x_data = 1.0 / np.asarray(g2b_stress)
y_data = np.asarray(g2b_dfdT_L)
fit = np.polyfit(x_data, y_data, 1)
g2b_fit_fn = np.poly1d(fit)
ax3.plot(x_plot, g2b_fit_fn(1/x_plot), color=g2b_pen, ls='--', lw=1)
print('\nG2b fit:', fit, ' rsq:', r_squared(y_data, g2b_fit_fn(x_data)))
# G3b
ax3.plot(g3b_stress, g3b_dfdT_L, color=g3b_pen, ls='none', lw=1, marker='o', ms=3, mec=g3b_pen, mfc=g3b_pen, mew=1)
# fitted expression
x_data = 1.0 / np.asarray(g3b_stress)
y_data = np.asarray(g3b_dfdT_L)
fit = np.polyfit(x_data, y_data, 1)
g3b_fit_fn = np.poly1d(fit)
ax3.plot(x_plot, g3b_fit_fn(1/x_plot), color=g3b_pen, ls='--', lw=1)
print('G3b fit:', fit, ' rsq:', r_squared(y_data, g3b_fit_fn(x_data)))
# G3c
ax3.plot(g3c_stress, g3c_dfdT_L, color=g3c_pen, ls='none', lw=1, marker='o', ms=3, mec=g3c_pen, mfc=g3c_pen, mew=1)
# fitted expression
x_data = 1.0 / np.asarray(g3c_stress)
y_data = np.asarray(g3c_dfdT_L)
fit = np.polyfit(x_data, y_data, 1)
g3c_fit_fn = np.poly1d(fit)
ax3.plot(x_plot, g3c_fit_fn(1/x_plot), color=g3c_pen, ls='--', lw=1)
print('G3c fit:', fit, ' rsq:', r_squared(y_data, g3c_fit_fn(x_data)))
# G5b
ax3.plot(g5b_stress, g5b_dfdT_L, color=g5b_pen, ls='none', lw=1, marker='o', ms=3, mec=g5b_pen, mfc=g5b_pen, mew=1)
# fitted expression
x_data = 1.0 / np.asarray(g5b_stress)
y_data = np.asarray(g5b_dfdT_L)
fit = np.polyfit(x_data, y_data, 1)
g5b_fit_fn = np.poly1d(fit)
ax3.plot(x_plot, g5b_fit_fn(1/x_plot), color=g5b_pen, ls='--', lw=1)
print('G5b fit:', fit, ' rsq:', r_squared(y_data, g5b_fit_fn(x_data)))
# G5c
ax3.plot(g5c_stress[:-1], g5c_dfdT_L[:-1], color=g5c_pen, ls='none', lw=1, marker='o', ms=3, mec=g5c_pen, mfc=g5c_pen, mew=1)
# fitted expression
x_data = 1.0 / np.asarray(g5c_stress[:-1])
y_data = np.asarray(g5c_dfdT_L[:-1])
fit = np.polyfit(x_data, y_data, 1)
g5c_fit_fn = np.poly1d(fit)
ax3.plot(x_plot, g5c_fit_fn(1/x_plot), color=g5c_pen, ls='--', lw=1)
print('G5c fit:', fit, ' rsq:', r_squared(y_data, g5c_fit_fn(x_data)))
# operating points
ax3.plot(gop_stress, gop_dfdT_L, color=op_pen, ls='none', marker=op_marker, ms=op_ms, mec=op_pen, mfc=op_mfc, mew=op_mew)
# plain lines for legend
ax3.plot([-2,-1], [-2,-1], color=g2b_pen, ls='-', lw=1, label=g2b_leg)
ax3.plot([-2,-1], [-2,-1], color=g3b_pen, ls='-', lw=1, label=g3_leg)
ax3.plot([-2,-1], [-2,-1], color=g5b_pen, ls='-', lw=1, label=g5_leg)
ax3.plot([-20,-10], [-20,-10], color=op_pen, ls='none', marker=op_marker, ms=op_ms, mec=op_pen, mfc=op_mfc, mew=op_mew, label=op_leg)
ax3.legend(loc='upper left', ncol = 1, prop={'size':8})
ax3.axis(axis_limits)
ax3.set_xticks(x_tick_spec)
ax3.set_yticks(y_tick_spec)
ax3.set_xlabel('Stress (MPa)')
ax3.set_ylabel('Constant length $df/dT$  (cent/$^\circ$C)')
#
plt.tight_layout()
ax1.text(0.87, 0.9,'(a)', transform=ax1.transAxes)
ax2.text(0.87, 0.9,'(b)', transform=ax2.transAxes)
ax3.text(0.87, 0.9,'(c)', transform=ax3.transAxes)
plt.savefig('./07abc_dfdT_L.eps', format='eps', dpi=1000)
plt.show()


# plot against 1/stress
fig=plt.figure(figsize=(12,3.9))
axis_limits = [0.003, 0.033, -4, 5]
x_tick_spec = np.arange(0.005, 0.035, 0.005)
y_tick_spec = np.arange(-4, 6, 1)
#
# Ground nylon
ax1=fig.add_subplot(131)
ax1.plot([0,300],[0,0],'k--',lw=1)  # Zero reference
arr_inv_stress = 1.0 / np.asarray(arr_stress)
# n8
ax1.plot(arr_inv_stress, n8_dfdT_L_est, color=n8_pen, ls='--', lw=1)
ax1.plot(n8_inv_stress, n8_dfdT_L, color=n8_pen, ls='none', lw=1, marker='o', ms=3, mec=n8_pen, mfc=n8_pen, mew=1)
# n14
ax1.plot(arr_inv_stress, n14_dfdT_L_est, color=n14_pen, ls='--', lw=1)
ax1.plot(n14_inv_stress, n14_dfdT_L, color=n14_pen, ls='none', lw=1, marker='o', ms=3, mec=n14_pen, mfc=n14_pen, mew=1)
# n5
ax1.plot(arr_inv_stress, n5_dfdT_L_est, color=n5_pen, ls='--', lw=1)
ax1.plot(n5_inv_stress, n5_dfdT_L, color=n5_pen, ls='none', lw=1, marker='o', ms=3, mec=n5_pen, mfc=n5_pen, mew=1)
# n23a
ax1.plot(arr_inv_stress, n23a_dfdT_L_est, color=n23a_pen, ls='--', lw=1)
ax1.plot(n23a_inv_stress, n23a_dfdT_L, color=n23a_pen, ls='none', lw=1, marker='o', ms=3, mec=n23a_pen, mfc=n23a_pen, mew=1)
# n29
ax1.plot(arr_inv_stress, n29_dfdT_L_est, color=n29_pen, ls='--', lw=1)
ax1.plot(n29_inv_stress, n29_dfdT_L, color=n29_pen, ls='none', lw=1, marker='o', ms=3, mec=n29_pen, mfc=n29_pen, mew=1)
# operating points
ax1.plot(nop_inv_stress, nop_dfdT_L, color=op_pen, ls='none', marker=op_marker, ms=op_ms, mec=op_pen, mfc=op_mfc, mew=op_mew)
# plain lines for legend
ax1.plot([-2,-1], [-2,-1], color=n8_pen, ls='-', lw=1, label=n8_leg)
ax1.plot([-2,-1], [-2,-1], color=n14_pen, ls='-', lw=1, label=n14_leg)
ax1.plot([-2,-1], [-2,-1], color=n5_pen, ls='-', lw=1, label=n5_leg)
ax1.plot([-2,-1], [-2,-1], color=n23a_pen, ls='-', lw=1, label=n23a_leg)
ax1.plot([-2,-1], [-2,-1], color=n29_pen, ls='-', lw=1, label=n29_leg)
ax1.plot([-20,-10], [-20,-10], color=op_pen, ls='none', marker=op_marker, ms=op_ms, mec=op_pen, mfc=op_mfc, mew=op_mew, label=op_leg)
ax1.legend(loc='lower left', ncol = 1, prop={'size':8})
ax1.axis(axis_limits)
ax1.set_xticks(x_tick_spec)
ax1.set_yticks(y_tick_spec)
ax1.set_xlabel('1/Stress (MPa$^{-1}$)')
ax1.set_ylabel('Constant length $df/dT$  (cent/$^\circ$C)')
#
# Carbon
ax2=fig.add_subplot(132)
ax2.plot([0,300],[0,0],'k--',lw=1)  # Zero reference
# C1
ax2.plot(c1_inv_stress, c1_dfdT_L, color=c1_pen, ls='none', lw=1, marker='o', ms=3, mec=c1_pen, mfc=c1_pen, mew=1)
ax2.plot(1/x_plot, c1_fit_fn(1/x_plot), color=c1_pen, ls='--', lw=1)
# C3
ax2.plot(c3_inv_stress, c3_dfdT_L, color=c3_pen, ls='none', lw=1, marker='o', ms=3, mec=c3_pen, mfc=c3_pen, mew=1)
ax2.plot(1/x_plot, c3_fit_fn(1/x_plot), color=c3_pen, ls='--', lw=1)
# C5b
ax2.plot(c5b_inv_stress, c5b_dfdT_L, color=c5b_pen, ls='none', lw=1, marker='o', ms=3, mec=c5b_pen, mfc=c5b_pen, mew=1)
ax2.plot(1/x_plot, c5b_fit_fn(1/x_plot), color=c5b_pen, ls='--', lw=1)
# C7a
ax2.plot(c7a_inv_stress, c7a_dfdT_L, color=c7a_pen, ls='none', lw=1, marker='o', ms=3, mec=c7a_pen, mfc=c7a_pen, mew=1)
ax2.plot(1/x_plot, c7a_fit_fn(1/x_plot), color=c7a_pen, ls='--', lw=1)
# operating points
ax2.plot(cop_inv_stress, cop_dfdT_L, color=op_pen, ls='none', marker=op_marker, ms=op_ms, mec=op_pen, mfc=op_mfc, mew=op_mew)
# plain lines for legend
ax2.plot([-2,-1], [-2,-1], color=c1_pen, ls='-', lw=1, label=c1_leg)
ax2.plot([-2,-1], [-2,-1], color=c3_pen, ls='-', lw=1, label=c3_leg)
ax2.plot([-2,-1], [-2,-1], color=c5b_pen, ls='-', lw=1, label=c5b_leg)
ax2.plot([-2,-1], [-2,-1], color=c7a_pen, ls='-', lw=1, label=c7a_leg)
ax2.plot([-20,-10], [-20,-10], color=op_pen, ls='none', marker=op_marker, ms=op_ms, mec=op_pen, mfc=op_mfc, mew=op_mew, label=op_leg)
ax2.legend(loc='lower left', ncol = 1, prop={'size':8})
ax2.axis(axis_limits)
ax2.set_xticks(x_tick_spec)
ax2.set_yticks(y_tick_spec)
ax2.set_xlabel('1/Stress (MPa$^{-1}$)')
ax2.set_ylabel('Constant length $df/dT$  (cent/$^\circ$C)')
#
# Gut
ax3=fig.add_subplot(133)
ax3.plot([0,300],[0,0],'k--',lw=1)  # Zero reference
# Data
ax3.plot(g2b_inv_stress, g2b_dfdT_L, color=g2b_pen, ls='none', lw=1, marker='o', ms=3, mec=g2b_pen, mfc=g2b_pen, mew=1)
ax3.plot(1/x_plot, g2b_fit_fn(1/x_plot), color=g2b_pen, ls='--', lw=1)
# G3b
ax3.plot(g3b_inv_stress, g3b_dfdT_L, color=g3b_pen, ls='none', lw=1, marker='o', ms=3, mec=g3b_pen, mfc=g3b_pen, mew=1)
ax3.plot(1/x_plot, g3b_fit_fn(1/x_plot), color=g3b_pen, ls='--', lw=1)
# G3c
ax3.plot(g3c_inv_stress, g3c_dfdT_L, color=g3c_pen, ls='none', lw=1, marker='o', ms=3, mec=g3c_pen, mfc=g3c_pen, mew=1)
ax3.plot(1/x_plot, g3c_fit_fn(1/x_plot), color=g3c_pen, ls='--', lw=1)
# G5b
ax3.plot(g5b_inv_stress, g5b_dfdT_L, color=g5b_pen, ls='none', lw=1, marker='o', ms=3, mec=g5b_pen, mfc=g5b_pen, mew=1)
ax3.plot(1/x_plot, g5b_fit_fn(1/x_plot), color=g5b_pen, ls='--', lw=1)
# G5c
ax3.plot(g5c_inv_stress[:-1], g5c_dfdT_L[:-1], color=g5c_pen, ls='none', lw=1, marker='o', ms=3, mec=g5c_pen, mfc=g5c_pen, mew=1)
ax3.plot(1/x_plot, g5c_fit_fn(1/x_plot), color=g5c_pen, ls='--', lw=1)
# operating points
ax3.plot(gop_inv_stress, gop_dfdT_L, color=op_pen, ls='none', marker=op_marker, ms=op_ms, mec=op_pen, mfc=op_mfc, mew=op_mew)
# plain lines for legend
ax3.plot([-2,-1], [-2,-1], color=g2b_pen, ls='-', lw=1, label=g2b_leg)
ax3.plot([-2,-1], [-2,-1], color=g3b_pen, ls='-', lw=1, label=g3_leg)
ax3.plot([-2,-1], [-2,-1], color=g5b_pen, ls='-', lw=1, label=g5_leg)
ax3.plot([-20,-10], [-20,-10], color=op_pen, ls='none', marker=op_marker, ms=op_ms, mec=op_pen, mfc=op_mfc, mew=op_mew, label=op_leg)
ax3.legend(loc='upper right', ncol = 1, prop={'size':8}, framealpha=1)
ax3.axis(axis_limits)
ax3.set_xticks(x_tick_spec)
ax3.set_yticks(y_tick_spec)
ax3.set_xlabel('1/Stress (MPa$^{-1}$)')
ax3.set_ylabel('Constant length $df/dT$  (cent/$^\circ$C)')
#
plt.tight_layout()
ax1.text(0.05, 0.9,'(a)', transform=ax1.transAxes)
ax2.text(0.05, 0.9,'(b)', transform=ax2.transAxes)
ax3.text(0.05, 0.9,'(c)', transform=ax3.transAxes)
plt.show()
```

```
N8, N14, N5 rsq: 0.993707112008
N23a, N29 rsq:   0.828662505642

C1 fit: [ 30.3558182   0.3238129]  rsq: 0.993602035041
C3 fit: [ 54.61570168   0.4921609 ]  rsq: 0.930979751047
C5b fit: [-27.32846115   0.40829536]  rsq: 0.915056375303
C7a fit: [-7.63123442  0.42063942]  rsq: 0.275260985553

G2b fit: [ -1.20289840e+02  -3.54965496e-02]  rsq: 0.997912196978
G3b fit: [-75.03903542   0.26235283]  rsq: 0.970360725168
G3c fit: [-35.39887563   0.07264282]  rsq: 0.870923419652
G5b fit: [-125.51621223   -0.18965061]  rsq: 0.999595679281
G5c fit: [-112.33216683   -0.27292576]  rsq: 0.998778229379
```

## $df/dT|\_F$ vs. stress¶

In [14]:

```
# plot against stress
fig=plt.figure(figsize=(12,3.9))
axis_limits = [0, 270, -1, 2.3]
x_tick_spec = np.arange(0, 300, 50)
y_tick_spec = np.arange(-1, 2.5, 0.5)
#
# Ground nylon
ax1=fig.add_subplot(131)
ax1.plot([0,300],[0,0],'k--',lw=1)  # Zero reference
# Data
ax1.plot(n8_stress, n8_dfdT_F, color=n8_pen, ls='-', lw=1, marker='o', ms=3, mec=n8_pen, mfc=n8_pen, mew=1)
ax1.plot(n14_stress, n14_dfdT_F, color=n14_pen, ls='-', lw=1, marker='o', ms=3, mec=n14_pen, mfc=n14_pen, mew=1)
ax1.plot(n5_stress, n5_dfdT_F, color=n5_pen, ls='-', lw=1, marker='o', ms=3, mec=n5_pen, mfc=n5_pen, mew=1)
ax1.plot(n23a_stress, n23a_dfdT_F, color=n23a_pen, ls='-', lw=1, marker='o', ms=3, mec=n23a_pen, mfc=n23a_pen, mew=1)
ax1.plot(n29_stress, n29_dfdT_F, color=n29_pen, ls='-', lw=1, marker='o', ms=3, mec=n29_pen, mfc=n29_pen, mew=1)
ax1.plot(nop_stress, nop_dfdT_F, color=op_pen, ls='none', marker=op_marker, ms=op_ms, mec=op_pen, mfc=op_mfc, mew=op_mew)
# plain lines for legend
ax1.plot([-2,-1], [-2,-1], color=n8_pen, ls='-', lw=1, label=n8_leg)
ax1.plot([-2,-1], [-2,-1], color=n14_pen, ls='-', lw=1, label=n14_leg)
ax1.plot([-2,-1], [-2,-1], color=n5_pen, ls='-', lw=1, label=n5_leg)
ax1.plot([-2,-1], [-2,-1], color=n23a_pen, ls='-', lw=1, label=n23a_leg)
ax1.plot([-2,-1], [-2,-1], color=n29_pen, ls='-', lw=1, label=n29_leg)
ax1.plot([-2,-1], [-2,-1], color=op_pen, ls='none', marker=op_marker, ms=op_ms, mec=op_pen, mfc=op_mfc, mew=op_mew, label=op_leg)
ax1.legend(loc='upper right', ncol = 1, prop={'size':8})
ax1.axis(axis_limits)
ax1.set_xticks(x_tick_spec)
ax1.set_yticks(y_tick_spec)
ax1.set_xlabel('Stress (MPa)')
ax1.set_ylabel('Constant tension $df/dT$  (cent/$^\circ$C)')
#
# Carbon
ax2=fig.add_subplot(132)
ax2.plot([0,300],[0,0],'k--',lw=1)  # Zero reference
# Data
ax2.plot(c1_stress, c1_dfdT_F, color=c1_pen, ls='-', lw=1, marker='o', ms=3, mec=c1_pen, mfc=c1_pen, mew=1)
ax2.plot(c3_stress, c3_dfdT_F, color=c3_pen, ls='-', lw=1, marker='o', ms=3, mec=c3_pen, mfc=c3_pen, mew=1)
ax2.plot(c5b_stress, c5b_dfdT_F, color=c5b_pen, ls='-', lw=1, marker='o', ms=3, mec=c5b_pen, mfc=c5b_pen, mew=1)
ax2.plot(c7a_stress, c7a_dfdT_F, color=c7a_pen, ls='-', lw=1, marker='o', ms=3, mec=c7a_pen, mfc=c7a_pen, mew=1)
ax2.plot(cop_stress, cop_dfdT_F, color=op_pen, ls='none', marker=op_marker, ms=op_ms, mfc=op_mfc, mew=op_mew)
# plain lines for legend
ax2.plot([-2,-1], [-2,-1], color=c1_pen, ls='-', lw=1, label=c1_leg)
ax2.plot([-2,-1], [-2,-1], color=c3_pen, ls='-', lw=1, label=c3_leg)
ax2.plot([-2,-1], [-2,-1], color=c5b_pen, ls='-', lw=1, label=c5b_leg)
ax2.plot([-2,-1], [-2,-1], color=c7a_pen, ls='-', lw=1, label=c7a_leg)
ax2.plot([-2,-1], [-2,-1], color=op_pen, ls='none', marker=op_marker, ms=op_ms, mec=op_pen, mfc=op_mfc, mew=op_mew, label=op_leg)
ax2.legend(loc='upper right', ncol = 1, prop={'size':8})
ax2.axis(axis_limits)
ax2.set_xticks(x_tick_spec)
ax2.set_yticks(y_tick_spec)
ax2.set_xlabel('Stress (MPa)')
ax2.set_ylabel('Constant tension $df/dT$  (cent/$^\circ$C)')
#
# Gut
ax3=fig.add_subplot(133)
ax3.plot([0,300],[0,0],'k--',lw=1)  # Zero reference
# Data
ax3.plot(g2b_stress, g2b_dfdT_F, color=g2b_pen, ls='--', lw=1, marker='o', ms=3, mec=g2b_pen, mfc=g2b_pen, mew=1)
ax3.plot(g3b_stress, g3b_dfdT_F, color=g3b_pen, ls='--', lw=1, marker='o', ms=3, mec=g3b_pen, mfc=g3b_pen, mew=1)
ax3.plot(g3c_stress, g3c_dfdT_F, color=g3c_pen, ls='--', lw=1, marker='o', ms=3, mec=g3c_pen, mfc=g3c_pen, mew=1)
ax3.plot(g5b_stress, g5b_dfdT_F, color=g5b_pen, ls='--', lw=1, marker='o', ms=3, mec=g5b_pen, mfc=g5b_pen, mew=1)
ax3.plot(g5c_stress[:-1], g5c_dfdT_F[:-1], color=g5c_pen, ls='--', lw=1, marker='o', ms=3, mec=g5c_pen, mfc=g5c_pen, mew=1)
ax3.plot(gop_stress, gop_dfdT_F, color=op_pen, ls='none', marker=op_marker, ms=op_ms, mec=op_pen, mfc=op_mfc, mew=op_mew)
# plain lines for legend
ax3.plot([-2,-1], [-2,-1], color=g2b_pen, ls='-', lw=1, label=g2b_leg)
ax3.plot([-2,-1], [-2,-1], color=g3b_pen, ls='-', lw=1, label=g3_leg)
ax3.plot([-2,-1], [-2,-1], color=g5b_pen, ls='-', lw=1, label=g5_leg)
ax3.plot([-2,-1], [-2,-1], color=op_pen, ls='none', marker=op_marker, ms=op_ms, mec=op_pen, mfc=op_mfc, mew=op_mew, label=op_leg)
ax3.legend(loc='lower right', ncol = 1, prop={'size':8})
ax3.axis(axis_limits)
ax3.set_xticks(x_tick_spec)
ax3.set_yticks(y_tick_spec)
ax3.set_xlabel('Stress (MPa)')
ax3.set_ylabel('Constant tension $df/dT$  (cent/$^\circ$C)')
#
plt.tight_layout()
ax1.text(0.05, 0.9,'(a)', transform=ax1.transAxes)
ax2.text(0.05, 0.9,'(b)', transform=ax2.transAxes)
ax3.text(0.05, 0.9,'(c)', transform=ax3.transAxes)
plt.savefig('./09abc_dfdT_F.eps', format='eps', dpi=1000)
plt.show()


# plot against 1/stress
fig=plt.figure(figsize=(12,3.9))
axis_limits = [0.003, 0.033, -1, 2.3]
x_tick_spec = np.arange(0.005, 0.035, 0.005)
y_tick_spec = np.arange(-1, 2.5, 0.5)
#
# Ground nylon
ax1=fig.add_subplot(131)
ax1.plot([0,300],[0,0],'k--',lw=1)  # Zero reference
# Data
ax1.plot(n8_inv_stress, n8_dfdT_F, color=n8_pen, ls='-', lw=1, marker='o', ms=3, mec=n8_pen, mfc=n8_pen, mew=1)
ax1.plot(n14_inv_stress, n14_dfdT_F, color=n14_pen, ls='-', lw=1, marker='o', ms=3, mec=n14_pen, mfc=n14_pen, mew=1)
ax1.plot(n5_inv_stress, n5_dfdT_F, color=n5_pen, ls='-', lw=1, marker='o', ms=3, mec=n5_pen, mfc=n5_pen, mew=1)
ax1.plot(n23a_inv_stress, n23a_dfdT_F, color=n23a_pen, ls='-', lw=1, marker='o', ms=3, mec=n23a_pen, mfc=n23a_pen, mew=1)
ax1.plot(n29_inv_stress, n29_dfdT_F, color=n29_pen, ls='-', lw=1, marker='o', ms=3, mec=n29_pen, mfc=n29_pen, mew=1)
ax1.plot(nop_inv_stress, nop_dfdT_F, color=op_pen, ls='none', marker=op_marker, ms=op_ms, mec=op_pen, mfc=op_mfc, mew=op_mew)
# plain lines for legend
ax1.plot([-2,-1], [-2,-1], color=n8_pen, ls='-', lw=1, label=n8_leg)
ax1.plot([-2,-1], [-2,-1], color=n14_pen, ls='-', lw=1, label=n14_leg)
ax1.plot([-2,-1], [-2,-1], color=n5_pen, ls='-', lw=1, label=n5_leg)
ax1.plot([-2,-1], [-2,-1], color=n23a_pen, ls='-', lw=1, label=n23a_leg)
ax1.plot([-2,-1], [-2,-1], color=n29_pen, ls='-', lw=1, label=n29_leg)
ax1.plot([-2,-1], [-2,-1], color=op_pen, ls='none', marker=op_marker, ms=op_ms, mec=op_pen, mfc=op_mfc, mew=op_mew, label=op_leg)
ax1.legend(loc='upper left', ncol = 1, prop={'size':8})
ax1.axis(axis_limits)
ax1.set_xticks(x_tick_spec)
ax1.set_yticks(y_tick_spec)
ax1.set_xlabel('1/Stress (MPa$^{-1}$)')
ax1.set_ylabel('Constant tension $df/dT$  (cent/$^\circ$C)')
#
# Carbon
ax2=fig.add_subplot(132)
ax2.plot([0,300],[0,0],'k--',lw=1)  # Zero reference
# Data
ax2.plot(c1_inv_stress, c1_dfdT_F, color=c1_pen, ls='-', lw=1, marker='o', ms=3, mec=c1_pen, mfc=c1_pen, mew=1)
ax2.plot(c3_inv_stress, c3_dfdT_F, color=c3_pen, ls='-', lw=1, marker='o', ms=3, mec=c3_pen, mfc=c3_pen, mew=1)
ax2.plot(c5b_inv_stress, c5b_dfdT_F, color=c5b_pen, ls='-', lw=1, marker='o', ms=3, mec=c5b_pen, mfc=c5b_pen, mew=1)
ax2.plot(c7a_inv_stress, c7a_dfdT_F, color=c7a_pen, ls='-', lw=1, marker='o', ms=3, mec=c7a_pen, mfc=c7a_pen, mew=1)
ax2.plot(cop_inv_stress, cop_dfdT_F, color=op_pen, ls='none', marker=op_marker, ms=op_ms, mec=op_pen, mfc=op_mfc, mew=op_mew)
# plain lines for legend
ax2.plot([-2,-1], [-2,-1], color=c1_pen, ls='-', lw=1, label=c1_leg)
ax2.plot([-2,-1], [-2,-1], color=c3_pen, ls='-', lw=1, label=c3_leg)
ax2.plot([-2,-1], [-2,-1], color=c5b_pen, ls='-', lw=1, label=c5b_leg)
ax2.plot([-2,-1], [-2,-1], color=c7a_pen, ls='-', lw=1, label=c7a_leg)
ax2.plot([-2,-1], [-2,-1], color=op_pen, ls='none', marker=op_marker, ms=op_ms, mec=op_pen, mfc=op_mfc, mew=op_mew, label=op_leg)
ax2.legend(loc='upper left', ncol = 1, prop={'size':8})
ax2.axis(axis_limits)
ax2.set_xticks(x_tick_spec)
ax2.set_yticks(y_tick_spec)
ax2.set_xlabel('1/Stress (MPa$^{-1}$)')
ax2.set_ylabel('Constant tension $df/dT$  (cent/$^\circ$C)')
#
# Gut
ax3=fig.add_subplot(133)
ax3.plot([0,300],[0,0],'k--',lw=1)  # Zero reference
# Data
ax3.plot(g2b_inv_stress, g2b_dfdT_F, color=g2b_pen, ls='--', lw=1, marker='o', ms=3, mec=g2b_pen, mfc=g2b_pen, mew=1)
ax3.plot(g3b_inv_stress, g3b_dfdT_F, color=g3b_pen, ls='--', lw=1, marker='o', ms=3, mec=g3b_pen, mfc=g3b_pen, mew=1)
ax3.plot(g3c_inv_stress, g3c_dfdT_F, color=g3c_pen, ls='--', lw=1, marker='o', ms=3, mec=g3c_pen, mfc=g3c_pen, mew=1)
ax3.plot(g5b_inv_stress, g5b_dfdT_F, color=g5b_pen, ls='--', lw=1, marker='o', ms=3, mec=g5b_pen, mfc=g5b_pen, mew=1)
ax3.plot(g5c_inv_stress[:-1], g5c_dfdT_F[:-1], color=g5c_pen, ls='--', lw=1, marker='o', ms=3, mec=g5c_pen, mfc=g5c_pen, mew=1)
ax3.plot(gop_inv_stress, gop_dfdT_F, color=op_pen, ls='none', marker=op_marker, ms=op_ms, mec=op_pen, mfc=op_mfc, mew=op_mew)
# plain lines for legend
ax3.plot([-2,-1], [-2,-1], color=g2b_pen, ls='-', lw=1, label=g2b_leg)
ax3.plot([-2,-1], [-2,-1], color=g3b_pen, ls='-', lw=1, label=g3_leg)
ax3.plot([-2,-1], [-2,-1], color=g5b_pen, ls='-', lw=1, label=g5_leg)
ax3.plot([-2,-1], [-2,-1], color=op_pen, ls='none', marker=op_marker, ms=op_ms, mec=op_pen, mfc=op_mfc, mew=op_mew, label=op_leg)
ax3.legend(loc='lower right', ncol = 1, prop={'size':8})
ax3.axis(axis_limits)
ax3.set_xticks(x_tick_spec)
ax3.set_yticks(y_tick_spec)
ax3.set_xlabel('1/Stress (MPa$^{-1}$)')
ax3.set_ylabel('Constant tension $df/dT$  (cent/$^\circ$C)')
#
plt.tight_layout()
ax1.text(0.87, 0.9,'(a)', transform=ax1.transAxes)
ax2.text(0.87, 0.9,'(b)', transform=ax2.transAxes)
ax3.text(0.87, 0.9,'(c)', transform=ax3.transAxes)
plt.show()
```

## Comparison between $\lambda = (1/F\_0)~dF/dT$ and $\psi = - (1/\mu\_0)~d\mu/dT$¶

In [15]:

```
# plot against 1/stress
fig=plt.figure(figsize=(12,7.4))
axis_limits = [0, 0.04, -4.3, 5]
x_tick_spec = np.arange(0, 0.05, .01)
y_tick_spec = np.arange(-4, 6, 1)
#
# Ground nylon
# lambda = (1/f).dF/dT
ax1=fig.add_subplot(231)
ax1.plot([0,300],[0,0],'k--',lw=1)  # Zero reference
ax1.plot(n8_inv_stress, n8_dFdTbyF, color=n8_pen, ls='-', lw=1, marker='o', ms=3, mec=n8_pen, mfc=n8_pen, mew=1)
ax1.plot(n14_inv_stress, n14_dFdTbyF, color=n14_pen, ls='-', lw=1, marker='o', ms=3, mec=n14_pen, mfc=n14_pen, mew=1)
ax1.plot(n5_inv_stress, n5_dFdTbyF, color=n5_pen, ls='-', lw=1, marker='o', ms=3, mec=n5_pen, mfc=n5_pen, mew=1)
ax1.plot(n23a_inv_stress, n23a_dFdTbyF, color=n23a_pen, ls='-', lw=1, marker='o', ms=3, mec=n23a_pen, mfc=n23a_pen, mew=1)
ax1.plot(n29_inv_stress, n29_dFdTbyF, color=n29_pen, ls='-', lw=1, marker='o', ms=3, mec=n29_pen, mfc=n29_pen, mew=1)
# plain lines for legend
ax1.plot([-2,-1], [-2,-1], color=n8_pen, ls='-', lw=1, label=n8_leg)
ax1.plot([-2,-1], [-2,-1], color=n14_pen, ls='-', lw=1, label=n14_leg)
ax1.plot([-2,-1], [-2,-1], color=n5_pen, ls='-', lw=1, label=n5_leg)
ax1.plot([-2,-1], [-2,-1], color=n23a_pen, ls='-', lw=1, label=n23a_leg)
ax1.plot([-2,-1], [-2,-1], color=n29_pen, ls='-', lw=1, label=n29_leg)
ax1.legend(loc='lower left', ncol = 1, prop={'size':8})
ax1.axis(axis_limits)
ax1.set_xticks(x_tick_spec)
ax1.set_yticks(y_tick_spec)
ax1.set_xlabel('1/Stress (MPa$^{-1}$)')
ax1.set_ylabel('$\lambda = 1/F_0$ $dF/dT$  ($10^{-3}/^\circ$C)')
#
# Ground nylon
# psi
ax4=fig.add_subplot(234)
ax4.plot([0,300],[0,0],'k--',lw=1)  # Zero reference
ax4.plot(n8_inv_stress, n8_psi, color=n8_pen, ls='-', lw=1, marker='o', ms=3, mec=n8_pen, mfc=n8_pen, mew=1)
ax4.plot(n14_inv_stress, n14_psi, color=n14_pen, ls='-', lw=1, marker='o', ms=3, mec=n14_pen, mfc=n14_pen, mew=1)
ax4.plot(n5_inv_stress, n5_psi, color=n5_pen, ls='-', lw=1, marker='o', ms=3, mec=n5_pen, mfc=n5_pen, mew=1)
ax4.plot(n23a_inv_stress, n23a_psi, color=n23a_pen, ls='-', lw=1, marker='o', ms=3, mec=n23a_pen, mfc=n23a_pen, mew=1)
ax4.plot(n29_inv_stress, n29_psi, color=n29_pen, ls='-', lw=1, marker='o', ms=3, mec=n29_pen, mfc=n29_pen, mew=1)
# plain lines for legend
ax4.plot([-2,-1], [-2,-1], color=n8_pen, ls='-', lw=1, label=n8_leg)
ax4.plot([-2,-1], [-2,-1], color=n14_pen, ls='-', lw=1, label=n14_leg)
ax4.plot([-2,-1], [-2,-1], color=n5_pen, ls='-', lw=1, label=n5_leg)
ax4.plot([-2,-1], [-2,-1], color=n23a_pen, ls='-', lw=1, label=n23a_leg)
ax4.plot([-2,-1], [-2,-1], color=n29_pen, ls='-', lw=1, label=n29_leg)
ax4.legend(loc='lower left', ncol = 1, prop={'size':8})
ax4.axis(axis_limits)
ax4.set_xticks(x_tick_spec)
ax4.set_yticks(y_tick_spec)
ax4.set_xlabel('1/Stress (MPa$^{-1}$)')
ax4.set_ylabel('$\psi = -1/ \mu_0$ $d \mu / dT$  ($10^{-3}/^\circ$C)')
#
# Carbon
# lambda = (1/f).dF/dT
ax2=fig.add_subplot(232)
ax2.plot([0,300],[0,0],'k--',lw=1)  # Zero reference
ax2.plot(c1_inv_stress, c1_dFdTbyF, color=c1_pen, ls='-', lw=1, marker='o', ms=3, mec=c1_pen, mfc=c1_pen, mew=1)
ax2.plot(c3_inv_stress, c3_dFdTbyF, color=c3_pen, ls='-', lw=1, marker='o', ms=3, mec=c3_pen, mfc=c3_pen, mew=1)
ax2.plot(c5b_inv_stress, c5b_dFdTbyF, color=c5b_pen, ls='-', lw=1, marker='o', ms=3, mec=c5b_pen, mfc=c5b_pen, mew=1)
ax2.plot(c7a_inv_stress, c7a_dFdTbyF, color=c7a_pen, ls='-', lw=1, marker='o', ms=3, mec=c7a_pen, mfc=c7a_pen, mew=1)
# plain lines for legend
ax2.plot([-2,-1], [-2,-1], color=c1_pen, ls='-', lw=1, label=c1_leg)
ax2.plot([-2,-1], [-2,-1], color=c3_pen, ls='-', lw=1, label=c3_leg)
ax2.plot([-2,-1], [-2,-1], color=c5b_pen, ls='-', lw=1, label=c5b_leg)
ax2.plot([-2,-1], [-2,-1], color=c7a_pen, ls='-', lw=1, label=c7a_leg)
ax2.legend(loc='lower left', ncol = 1, prop={'size':8})
ax2.axis(axis_limits)
ax2.set_xticks(x_tick_spec)
ax2.set_yticks(y_tick_spec)
ax2.set_xlabel('1/Stress (MPa$^{-1}$)')
ax2.set_ylabel('$\lambda = 1/F_0$ $dF/dT$  ($10^{-3}/^\circ$C)')
#
# Carbon
# psi
ax5=fig.add_subplot(235)
ax5.plot([0,300],[0,0],'k--',lw=1)  # Zero reference
ax5.plot(c1_inv_stress, c1_psi, color=c1_pen, ls='-', lw=1, marker='o', ms=3, mec=c1_pen, mfc=c1_pen, mew=1)
ax5.plot(c3_inv_stress, c3_psi, color=c3_pen, ls='-', lw=1, marker='o', ms=3, mec=c3_pen, mfc=c3_pen, mew=1)
ax5.plot(c5b_inv_stress, c5b_psi, color=c5b_pen, ls='-', lw=1, marker='o', ms=3, mec=c5b_pen, mfc=c5b_pen, mew=1)
ax5.plot(c7a_inv_stress, c7a_psi, color=c7a_pen, ls='-', lw=1, marker='o', ms=3, mec=c7a_pen, mfc=c7a_pen, mew=1)
# plain lines for legend
ax5.plot([-2,-1], [-2,-1], color=c1_pen, ls='-', lw=1, label=c1_leg)
ax5.plot([-2,-1], [-2,-1], color=c3_pen, ls='-', lw=1, label=c3_leg)
ax5.plot([-2,-1], [-2,-1], color=c5b_pen, ls='-', lw=1, label=c5b_leg)
ax5.plot([-2,-1], [-2,-1], color=c7a_pen, ls='-', lw=1, label=c7a_leg)
ax5.legend(loc='lower left', ncol = 1, prop={'size':8})
ax5.axis(axis_limits)
ax5.set_xticks(x_tick_spec)
ax5.set_yticks(y_tick_spec)
ax5.set_xlabel('1/Stress (MPa$^{-1}$)')
ax5.set_ylabel('$\psi = -1/ \mu_0$ $d \mu / dT$  ($10^{-3}/^\circ$C)')
#
# Gut
# lambda = (1/f).dF/dT
ax3=fig.add_subplot(233)
ax3.plot([0,300],[0,0],'k--',lw=1)  # Zero reference
ax3.plot(g2b_inv_stress, g2b_dFdTbyF, color=g2b_pen, ls='--', lw=1, marker='o', ms=3, mec=g2b_pen, mfc=g2b_pen, mew=1)
ax3.plot(g3b_inv_stress, g3b_dFdTbyF, color=g3b_pen, ls='--', lw=1, marker='o', ms=3, mec=g3b_pen, mfc=g3b_pen, mew=1)
ax3.plot(g3c_inv_stress, g3c_dFdTbyF, color=g3c_pen, ls='--', lw=1, marker='o', ms=3, mec=g3c_pen, mfc=g3c_pen, mew=1)
ax3.plot(g5b_inv_stress, g5b_dFdTbyF, color=g5b_pen, ls='--', lw=1, marker='o', ms=3, mec=g5b_pen, mfc=g5b_pen, mew=1)
ax3.plot(g5c_inv_stress[:-1], g5c_dFdTbyF[:-1], color=g5c_pen, ls='--', lw=1, marker='o', ms=3, mec=g5c_pen, mfc=g5c_pen, mew=1)
ax3.plot([g5c_inv_stress[-3],g5c_inv_stress[-1]], [g5c_dFdTbyF[-3],g5c_dFdTbyF[-1]], color=g5c_pen, ls=':', lw=1, marker='o', ms=3, mec=g5c_pen, mfc=g5c_pen, mew=1)
# plain lines for legend
ax3.plot([-2,-1], [-2,-1], color=g2b_pen, ls='-', lw=1, label=g2b_leg)
ax3.plot([-2,-1], [-2,-1], color=g3b_pen, ls='-', lw=1, label=g3_leg)
ax3.plot([-2,-1], [-2,-1], color=g5b_pen, ls='-', lw=1, label=g5_leg)
ax3.legend(loc='upper left', ncol = 1, prop={'size':8})
ax3.axis(axis_limits)
ax3.set_xticks(x_tick_spec)
ax3.set_yticks(y_tick_spec)
ax3.set_xlabel('1/Stress (MPa$^{-1}$)')
ax3.set_ylabel('$\lambda = 1/F_0$ $dF/dT$  ($10^{-3}/^\circ$C)')
#
# Gut
# psi
ax6=fig.add_subplot(236)
ax6.plot([0,300],[0,0],'k--',lw=1)  # Zero reference
ax6.plot(g2b_inv_stress, g2b_psi, color=g2b_pen, ls='--', lw=1, marker='o', ms=3, mec=g2b_pen, mfc=g2b_pen, mew=1)
ax6.plot(g3b_inv_stress, g3b_psi, color=g3b_pen, ls='--', lw=1, marker='o', ms=3, mec=g3b_pen, mfc=g3b_pen, mew=1)
ax6.plot(g3c_inv_stress, g3c_psi, color=g3c_pen, ls='--', lw=1, marker='o', ms=3, mec=g3c_pen, mfc=g3c_pen, mew=1)
ax6.plot(g5b_inv_stress, g5b_psi, color=g5b_pen, ls='--', lw=1, marker='o', ms=3, mec=g5b_pen, mfc=g5b_pen, mew=1)
ax6.plot(g5c_inv_stress[:-1], g5c_psi[:-1], color=g5c_pen, ls='--', lw=1, marker='o', ms=3, mec=g5c_pen, mfc=g5c_pen, mew=1)
ax6.plot([g5c_inv_stress[-3],g5c_inv_stress[-1]], [g5c_psi[-3],g5c_psi[-1]], color=g5c_pen, ls=':', lw=1, marker='o', ms=3, mec=g5c_pen, mfc=g5c_pen, mew=1)
# plain lines for legend
ax6.plot([-2,-1], [-2,-1], color=g2b_pen, ls='-', lw=1, label=g2b_leg)
ax6.plot([-2,-1], [-2,-1], color=g3b_pen, ls='-', lw=1, label=g3_leg)
ax6.plot([-2,-1], [-2,-1], color=g5b_pen, ls='-', lw=1, label=g5_leg)
ax6.legend(loc='upper left', ncol = 1, prop={'size':8})
ax6.axis(axis_limits)
ax6.set_xticks(x_tick_spec)
ax6.set_yticks(y_tick_spec)
ax6.set_xlabel('1/Stress (MPa$^{-1}$)')
ax6.set_ylabel('$\psi = -1/ \mu_0$ $d \mu / dT$  ($10^{-3}/^\circ$C)')
#
plt.tight_layout()
ax1.text(0.87, 0.9,'(a)', transform=ax1.transAxes)
ax2.text(0.87, 0.9,'(b)', transform=ax2.transAxes)
ax3.text(0.87, 0.9,'(c)', transform=ax3.transAxes)
ax4.text(0.87, 0.9,'(d)', transform=ax4.transAxes)
ax5.text(0.87, 0.9,'(e)', transform=ax5.transAxes)
ax6.text(0.87, 0.9,'(f)', transform=ax6.transAxes)
plt.savefig('./10abcdef_lambda_and_psi.eps', format='eps', dpi=1000)
plt.show()


# check sums
# plot against 1/stress
fig=plt.figure(figsize=(12,3.9))
axis_limits = [0, 0.04, -4, 5]
x_tick_spec = np.arange(0, 0.05, .01)
y_tick_spec = np.arange(-4, 6, 1)
scaler = 0.6 / np.log(2.0)
#
# Ground nylon
ax1=fig.add_subplot(131)
ax1.plot([0,300],[0,0],'k--',lw=1)  # Zero reference
# df/dT|L
ax1.plot(n8_inv_stress, n8_dfdT_L, color=n8_pen, ls='-', lw=1, marker='o', ms=3, mec=n8_pen, mfc=n8_pen, mew=1)
ax1.plot(n14_inv_stress, n14_dfdT_L, color=n14_pen, ls='-', lw=1, marker='o', ms=3, mec=n14_pen, mfc=n14_pen, mew=1)
ax1.plot(n5_inv_stress, n5_dfdT_L, color=n5_pen, ls='-', lw=1, marker='o', ms=3, mec=n5_pen, mfc=n5_pen, mew=1)
ax1.plot(n23a_inv_stress, n23a_dfdT_L, color=n23a_pen, ls='-', lw=1, marker='o', ms=3, mec=n23a_pen, mfc=n23a_pen, mew=1)
ax1.plot(n29_inv_stress, n29_dfdT_L, color=n29_pen, ls='-', lw=1, marker='o', ms=3, mec=n29_pen, mfc=n29_pen, mew=1)
# sums
n8_sum = scaler * (n8_dFdTbyF + np.asarray(n8_psi))
n14_sum = scaler * (n14_dFdTbyF + np.asarray(n14_psi))
n5_sum = scaler * (n5_dFdTbyF + np.asarray(n5_psi))
n23a_sum = scaler * (n23a_dFdTbyF + np.asarray(n23a_psi))
n29_sum = scaler * (n29_dFdTbyF + np.asarray(n29_psi))
ax1.plot(n8_inv_stress, n8_sum, color=n8_pen, ls='-', lw=1, marker='o', ms=3, mec=n8_pen, mfc='none', mew=1)
ax1.plot(n14_inv_stress, n14_sum, color=n14_pen, ls='-', lw=1, marker='o', ms=3, mec=n14_pen, mfc='none', mew=1)
ax1.plot(n5_inv_stress, n5_sum, color=n5_pen, ls='-', lw=1, marker='o', ms=3, mec=n5_pen, mfc='none', mew=1)
ax1.plot(n23a_inv_stress, n23a_sum, color=n23a_pen, ls='-', lw=1, marker='o', ms=3, mec=n23a_pen, mfc='none', mew=1)
ax1.plot(n29_inv_stress, n29_sum, color=n29_pen, ls='-', lw=1, marker='o', ms=3, mec=n29_pen, mfc='none', mew=1)
# plain lines for legend
ax1.plot([-2,-1], [-2,-1], color=n8_pen, ls='-', lw=1, label=n8_leg)
ax1.plot([-2,-1], [-2,-1], color=n14_pen, ls='-', lw=1, label=n14_leg)
ax1.plot([-2,-1], [-2,-1], color=n5_pen, ls='-', lw=1, label=n5_leg)
ax1.plot([-2,-1], [-2,-1], color=n23a_pen, ls='-', lw=1, label=n23a_leg)
ax1.plot([-2,-1], [-2,-1], color=n29_pen, ls='-', lw=1, label=n29_leg)
ax1.legend(loc='lower left', ncol = 1, prop={'size':8})
ax1.axis(axis_limits)
ax1.set_xticks(x_tick_spec)
ax1.set_yticks(y_tick_spec)
ax1.set_xlabel('1/Stress (MPa$^{-1}$)')
ax1.set_ylabel('Constant length $df/dT$  (cent/$^\circ$C)')
#
# Carbon
ax2=fig.add_subplot(132)
ax2.plot([0,300],[0,0],'k--',lw=1)  # Zero reference
# df/dT|L
ax2.plot(c1_inv_stress, c1_dfdT_L, color=c1_pen, ls='-', lw=1, marker='o', ms=3, mec=c1_pen, mfc=c1_pen, mew=1)
ax2.plot(c3_inv_stress, c3_dfdT_L, color=c3_pen, ls='-', lw=1, marker='o', ms=3, mec=c3_pen, mfc=c3_pen, mew=1)
ax2.plot(c5b_inv_stress, c5b_dfdT_L, color=c5b_pen, ls='-', lw=1, marker='o', ms=3, mec=c5b_pen, mfc=c5b_pen, mew=1)
ax2.plot(c7a_inv_stress, c7a_dfdT_L, color=c7a_pen, ls='-', lw=1, marker='o', ms=3, mec=c7a_pen, mfc=c7a_pen, mew=1)
# sums
c1_sum = scaler * (c1_dFdTbyF + np.asarray(c1_psi))
c3_sum = scaler * (c3_dFdTbyF + np.asarray(c3_psi))
c5b_sum = scaler * (c5b_dFdTbyF + np.asarray(c5b_psi))
c7a_sum = scaler * (c7a_dFdTbyF + np.asarray(c7a_psi))
ax2.plot(c1_inv_stress, c1_sum, color=c1_pen, ls='-', lw=1, marker='o', ms=3, mec=c1_pen, mfc='none', mew=2)
ax2.plot(c3_inv_stress, c3_sum, color=c3_pen, ls='-', lw=1, marker='o', ms=3, mec=c3_pen, mfc='none', mew=2)
ax2.plot(c5b_inv_stress, c5b_sum, color=c5b_pen, ls='-', lw=1, marker='o', ms=3, mec=c5b_pen, mfc='none', mew=2)
ax2.plot(c7a_inv_stress, c7a_sum, color=c7a_pen, ls='-', lw=1, marker='o', ms=3, mec=c7a_pen, mfc='none', mew=2)
# plain lines for legend
ax2.plot([-2,-1], [-2,-1], color=c1_pen, ls='-', lw=1, label=c1_leg)
ax2.plot([-2,-1], [-2,-1], color=c3_pen, ls='-', lw=1, label=c3_leg)
ax2.plot([-2,-1], [-2,-1], color=c5b_pen, ls='-', lw=1, label=c5b_leg)
ax2.plot([-2,-1], [-2,-1], color=c7a_pen, ls='-', lw=1, label=c7a_leg)
ax2.legend(loc='lower left', ncol = 1, prop={'size':8})
ax2.axis(axis_limits)
ax2.set_xticks(x_tick_spec)
ax2.set_yticks(y_tick_spec)
ax2.set_xlabel('1/Stress (MPa$^{-1}$)')
ax2.set_ylabel('Constant length $df/dT$  (cent/$^\circ$C)')
#
# Gut
ax3=fig.add_subplot(133)
ax3.plot([0,300],[0,0],'k--',lw=1)  # Zero reference
# df/dT|L
ax3.plot(g2b_inv_stress, g2b_dfdT_L, color=g2b_pen, ls='-', lw=1, marker='o', ms=3, mec=g2b_pen, mfc=g2b_pen, mew=1)
ax3.plot(g3b_inv_stress, g3b_dfdT_L, color=g3b_pen, ls='-', lw=1, marker='o', ms=3, mec=g3b_pen, mfc=g3b_pen, mew=1)
ax3.plot(g3c_inv_stress, g3c_dfdT_L, color=g3c_pen, ls='-', lw=1, marker='o', ms=3, mec=g3c_pen, mfc=g3c_pen, mew=1)
ax3.plot(g5b_inv_stress, g5b_dfdT_L, color=g5b_pen, ls='-', lw=1, marker='o', ms=3, mec=g5b_pen, mfc=g5b_pen, mew=1)
ax3.plot(g5c_inv_stress[:-1], g5c_dfdT_L[:-1], color=g5c_pen, ls='-', lw=1, marker='o', ms=3, mec=g5c_pen, mfc=g5c_pen, mew=1)
# sums
g2b_sum = scaler * (g2b_dFdTbyF + np.asarray(g2b_psi))
g3b_sum = scaler * (g3b_dFdTbyF + np.asarray(g3b_psi))
g3c_sum = scaler * (g3c_dFdTbyF + np.asarray(g3c_psi))
g5b_sum = scaler * (g5b_dFdTbyF + np.asarray(g5b_psi))
g5c_sum = scaler * (g5c_dFdTbyF + np.asarray(g5c_psi))
ax3.plot(g2b_inv_stress, g2b_sum, color=g2b_pen, ls='-', lw=1, marker='o', ms=3, mec=g2b_pen, mfc='none', mew=1)
ax3.plot(g3b_inv_stress, g3b_sum, color=g3b_pen, ls='-', lw=1, marker='o', ms=3, mec=g3b_pen, mfc='none', mew=1)
ax3.plot(g3c_inv_stress, g3c_sum, color=g3c_pen, ls='-', lw=1, marker='o', ms=3, mec=g3c_pen, mfc='none', mew=1)
ax3.plot(g5b_inv_stress, g5b_sum, color=g5b_pen, ls='-', lw=1, marker='o', ms=3, mec=g5b_pen, mfc='none', mew=1)
ax3.plot(g5c_inv_stress[:-1], g5c_sum[:-1], color=g5c_pen, ls='-', lw=1, marker='o', ms=3, mec=g5c_pen, mfc='none', mew=1)
# plain lines for legend
ax3.plot([-2,-1], [-2,-1], color=g2b_pen, ls='-', lw=1, label=g2b_leg)
ax3.plot([-2,-1], [-2,-1], color=g3b_pen, ls='-', lw=1, label=g3_leg)
ax3.plot([-2,-1], [-2,-1], color=g5b_pen, ls='-', lw=1, label=g5_leg)
ax3.legend(loc='upper left', ncol = 1, prop={'size':8}, framealpha=1)
ax3.axis(axis_limits)
ax3.set_xticks(x_tick_spec)
ax3.set_yticks(y_tick_spec)
ax3.set_xlabel('1/Stress (MPa$^{-1}$)')
ax3.set_ylabel('Constant length $df/dT$  (cent/$^\circ$C)')
#
plt.tight_layout()
ax1.text(0.87, 0.9,'(a)', transform=ax1.transAxes)
ax2.text(0.87, 0.9,'(b)', transform=ax2.transAxes)
ax3.text(0.87, 0.9,'(c)', transform=ax3.transAxes)
plt.show()
```

## CLTE vs. stress¶

In [16]:

```
# plot just for nylon and carbon
fig=plt.figure(figsize=(8,3.9))
axis_limits = [0, 270, -680, 0]
x_tick_spec = np.arange(0, 300, 50)
y_tick_spec = np.arange(-600, 100, 100)
#
# Ground nylon
ax1=fig.add_subplot(121)
# CLTE
ax1.plot(n8_stress, n8_CLTE, color=n8_pen, ls='-', lw=1, marker='o', ms=3, mec=n8_pen, mfc=n8_pen, mew=1)
ax1.plot(n14_stress, n14_CLTE, color=n14_pen, ls='-', lw=1, marker='o', ms=3, mec=n14_pen, mfc=n14_pen, mew=1)
ax1.plot(n5_stress, n5_CLTE, color=n5_pen, ls='-', lw=1, marker='o', ms=3, mec=n5_pen, mfc=n5_pen, mew=1)
ax1.plot(n23a_stress, n23a_CLTE, color=n23a_pen, ls='-', lw=1, marker='o', ms=3, mec=n23a_pen, mfc=n23a_pen, mew=1)
ax1.plot(n29_stress, n29_CLTE, color=n29_pen, ls='-', lw=1, marker='o', ms=3, mec=n29_pen, mfc=n29_pen, mew=1)
# plain lines for legend
ax1.plot([-2,-1], [-2,-1], color=n8_pen, ls='-', lw=1, label=n8_leg)
ax1.plot([-2,-1], [-2,-1], color=n14_pen, ls='-', lw=1, label=n14_leg)
ax1.plot([-2,-1], [-2,-1], color=n5_pen, ls='-', lw=1, label=n5_leg)
ax1.plot([-2,-1], [-2,-1], color=n23a_pen, ls='-', lw=1, label=n23a_leg)
ax1.plot([-2,-1], [-2,-1], color=n29_pen, ls='-', lw=1, label=n29_leg)
ax1.legend(loc='lower right', ncol = 1, prop={'size':8})
ax1.axis(axis_limits)
ax1.set_xticks(x_tick_spec)
ax1.set_yticks(y_tick_spec)
ax1.set_xlabel('Stress (MPa)')
ax1.set_ylabel(r'CLTE $\alpha$  ($10^{-6}/^\circ$C)')
#
# Carbon
ax2=fig.add_subplot(122)
# CLTE
ax2.plot(c1_stress, c1_CLTE, color=c1_pen, ls='-', lw=1, marker='o', ms=3, mec=c1_pen, mfc=c1_pen, mew=1)
ax2.plot(c3_stress, c3_CLTE, color=c3_pen, ls='-', lw=1, marker='o', ms=3, mec=c3_pen, mfc=c3_pen, mew=1)
ax2.plot(c5b_stress, c5b_CLTE, color=c5b_pen, ls='-', lw=1, marker='o', ms=3, mec=c5b_pen, mfc=c5b_pen, mew=1)
ax2.plot(c7a_stress, c7a_CLTE, color=c7a_pen, ls='-', lw=1, marker='o', ms=3, mec=c7a_pen, mfc=c7a_pen, mew=1)
# plain lines for legend
ax2.plot([-2,-1], [-2,-1], color=c1_pen, ls='-', lw=1, label=c1_leg)
ax2.plot([-2,-1], [-2,-1], color=c3_pen, ls='-', lw=1, label=c3_leg)
ax2.plot([-2,-1], [-2,-1], color=c5b_pen, ls='-', lw=1, label=c5b_leg)
ax2.plot([-2,-1], [-2,-1], color=c7a_pen, ls='-', lw=1, label=c7a_leg)
ax2.legend(loc='lower right', ncol = 1, prop={'size':8})
ax2.axis(axis_limits)
ax2.set_xticks(x_tick_spec)
ax2.set_yticks(y_tick_spec)
ax2.set_xlabel('Stress (MPa)')
ax2.set_ylabel(r'CLTE $\alpha$  ($10^{-6}/^\circ$C)')
#
plt.tight_layout()
ax1.text(0.05, 0.9,'(a)', transform=ax1.transAxes)
ax2.text(0.05, 0.9,'(b)', transform=ax2.transAxes)
plt.savefig('./11ab_CLTE2.eps', format='eps', dpi=1000)
plt.show()
```
