# Supplementary material for: Comparison of Mechanical Properties of Natural Gut and Synthetic Polymer Harp Strings
Source: Materials (Basel). 2018 Nov 1;11(11):2160. doi: 10.3390/ma11112160 (PMC6266393; doi:10.3390/ma11112160)
Supplement: Supplementary file 1 [file materials-11-02160-s001.zip › Figures+for+extended+gut+tests.html]

Figures for extended gut tests


# Figures for extended tests with gut strings¶

In [1]:

```
import xlrd
import numpy as np
import matplotlib.pyplot as plt
# set latex expressions to same font as other text
#import matplotlib
#matplotlib.rcParams['mathtext.fontset'] = 'custom'
#matplotlib.rcParams['mathtext.rm'] = 'Bitstream Vera Sans'
#matplotlib.rcParams['mathtext.it'] = 'Bitstream Vera Sans:italic'
#matplotlib.rcParams['mathtext.bf'] = 'Bitstream Vera Sans:bold'
#matplotlib.rcParams['mathtext.cal'] = 'Bitstream Vera Sans:cursive'
# enable plots to be shown in cells
%matplotlib inline
#
import math

from datetime import datetime
FMT_time = '%H:%M:%S'
FMT_date = '%Y/%m/%d'

def read_data_file(filename, time_array, frequency_array, tension_array, temperature_array, RH_array, AH_array):
    global start_date
    global start_time
    f = open(filename, "r")
    # read in data
    data = f.readlines()
    found_start = False
    for line in data:
        words = line.split(',')
        if((found_start == True) and (words[2] == 'ok') and (words[3] == 'ok')):
            line_date = datetime.strptime(words[0],FMT_date)
            line_time = datetime.strptime(words[1],FMT_time)
            if(len(time_array)==0):
                start_date = line_date
                start_time = line_time
                time_array.append(0.0)
            else:
                date_diff = line_date - start_date
                time_diff = line_time - start_time # could be negative
                days = date_diff.days + time_diff.days + time_diff.seconds / (24.0 * 3600.0)
                time_array.append(days)
            frequency_array.append(float(words[4]))
            tension_array.append(float(words[5]))
            temperature_array.append(float(words[7]))
            RH_array.append(float(words[8]))
            AH_array.append(float(words[9]))
        if('No_Adjustment_mode' in words[0]):
            found_start = True
    # close file
    f.close()

def locate_time_index(time_array, search_time, start_index): 
    # returns 0 if end of time_array reached before search_time found
    finished = False
    found_it = False
    scan_index = start_index - 1
    while((found_it == False) and (finished == False)):
        scan_index += 1;
        if(scan_index == len(time_array)):
            finished = True
        elif(time_array[scan_index] >= search_time):
            found_it = True
    if(finished == False):
        return scan_index
    else:
        return 0
    
def derived_responses(time_array, freq_array, tension_array, temp_array, RH_array, AH_array, dev_array, target_freq,
                      lin_den_array, vibr_length, avge_time_array, avge_dev_array, avge_tension_array, avge_lin_den_array,
                      avge_temp_array, avge_RH_array, avge_AH_array):
    four_Lv_sq = 4.0 * vibr_length * vibr_length
    for i in range(0, len(freq_array), 1):
        dev_array.append(1200.0 * math.log(freq_array[i] / target_freq, 2.0))
        lin_den_array.append(1000.0 * tension_array[i] / (four_Lv_sq * freq_array[i] * freq_array[i])) # now in g/m
    finished = False
    start_index = -1
    while(finished == False):
        start_index += 1
        search_time = time_array[start_index] + 1.0
        stop_index = locate_time_index(time_array, search_time, start_index)
        if(stop_index == 0):
            finished = True
        else:
            avge_time_array.append(time_array[start_index] + 0.5)
            avge_dev_array.append(np.average(dev_array[start_index:stop_index+1]))
            avge_tension_array.append(np.average(tension_array[start_index:stop_index+1]))
            avge_lin_den_array.append(np.average(lin_den_array[start_index:stop_index+1]))
            avge_temp_array.append(np.average(temp_array[start_index:stop_index+1]))
            avge_RH_array.append(np.average(RH_array[start_index:stop_index+1]))
            avge_AH_array.append(np.average(AH_array[start_index:stop_index+1]))

def process_data_file(time_array, temp_array, AH_array, RH_array, tension_array, y_array, max_span, fit_array, order, dydT_array, 
                      mid_time_array, avg_temp_array, avg_AH_array, avg_RH_array, avg_tension_array, maxmin_time, maxmin_temp):
    # scan for temperature maxima and minima
    # maxima
    finished = False
    day1_index = locate_time_index(time_array, time_array[0] + max_span, 0)
    if(day1_index > 0):        
        index2 = np.argmax(temp_array[0:day1_index])
        maxmin_time.append(time_array[index2])
        maxmin_temp.append(temp_array[index2])
        while(finished == False):
            # locate array indeces for next temperature cycle
            index1 = index2
            start_index = locate_time_index(time_array, time_array[index1] + 0.5, index1)
            stop_index = locate_time_index(time_array, time_array[index1] + 1.5, start_index)
            if(stop_index == 0):
                finished = True
            else:
                index2 = start_index + np.argmax(temp_array[start_index:stop_index])
                maxmin_time.append(time_array[index2])
                maxmin_temp.append(temp_array[index2])
                # calculate dy/dT and average values for cycle between index1 and index2
                mid_time_array.append((time_array[index1] + time_array[index2]) / 2.0)
                average_temp = np.average(temp_array[index1:index2+1])
                avg_temp_array.append(average_temp)
                avg_AH_array.append(np.average(AH_array[index1:index2+1]))
                avg_RH_array.append(np.average(RH_array[index1:index2+1]))
                avg_tension_array.append(np.average(tension_array[index1:index2+1]))
                fit = np.polyfit(temp_array[index1:index2+1], y_array[index1:index2+1], order)
                if(order == 1):
                    dydT_value = fit[0]
                elif(order == 2):
                    dydT_value = 2.0 * fit[0] * average_temp + fit[1] # differentiate to get gradient
                else:
                    dydT_value = 0.0
                dydT_array.append(dydT_value)
                fit_array.append(fit)
        # minima
        finished = False
        day1_index = locate_time_index(time_array, time_array[0] + 1.0, 0)
        index2 = np.argmin(temp_array[0:day1_index])      
        maxmin_time.append(time_array[index2])
        maxmin_temp.append(temp_array[index2])
        while(finished == False):
            # locate array indeces for next temperature cycle
            index1 = index2
            start_index = locate_time_index(time_array, time_array[index1] + 0.5, index1)
            stop_index = locate_time_index(time_array, time_array[index1] + 1.5, start_index)
            if(stop_index == 0):
                finished = True
            else:
                index2 = start_index + np.argmin(temp_array[start_index:stop_index])
                maxmin_time.append(time_array[index2])
                maxmin_temp.append(temp_array[index2])
                 # calculate dy/dT and average values for cycle between index1 and index2
                mid_time_array.append((time_array[index1] + time_array[index2]) / 2.0)
                average_temp = np.average(temp_array[index1:index2+1])
                avg_temp_array.append(average_temp)
                avg_AH_array.append(np.average(AH_array[index1:index2+1]))
                avg_RH_array.append(np.average(RH_array[index1:index2+1]))
                avg_tension_array.append(np.average(tension_array[index1:index2+1]))
                fit = np.polyfit(temp_array[index1:index2+1], y_array[index1:index2+1], order)
                if(order == 1):
                    dydT_value = fit[0]
                elif(order == 2):
                    dydT_value = 2.0 * fit[0] * average_temp + fit[1] # differentiate to get gradient
                else:
                    dydT_value = 0.0
                dydT_array.append(dydT_value)
                fit_array.append(fit)

    
# load data for G5b @ 324 Hz
time_1 = []
freq_1 = []
tens_1 = []
temp_1 = []
RH_1 = []
AH_1= []
read_data_file(".\string_test_data_2015.09.03.08.19.csv", time_1, freq_1, tens_1, temp_1, RH_1, AH_1)
read_data_file(".\string_test_data_2015.09.07.19.23.csv", time_1, freq_1, tens_1, temp_1, RH_1, AH_1)
# derived responses
dev_1 = []
mu_1 = []
avge_time_1 = []
avge_dev_1 = []
avge_tens_1 = []
avge_mu_1 = []
avge_temp_1 = []
avge_RH_1 = []
avge_AH_1 = []
derived_responses(time_1, freq_1, tens_1, temp_1, RH_1, AH_1, dev_1, 324.0, mu_1, 0.4985,
                  avge_time_1, avge_dev_1, avge_tens_1, avge_mu_1, avge_temp_1, avge_RH_1, avge_AH_1)

# load data for G5c @ 324 Hz
time_2 = []
freq_2 = []
tens_2 = []
temp_2 = []
RH_2 = []
AH_2 = []
read_data_file(".\string_test_data_2017.11.12.10.51.csv", time_2, freq_2, tens_2, temp_2, RH_2, AH_2)
# derived responses
dev_2 = []
mu_2 = []                             
avge_time_2 = []
avge_dev_2 = []
avge_tens_2 = []
avge_mu_2 = []
avge_temp_2 = []
avge_RH_2 = []
avge_AH_2 = []
derived_responses(time_2, freq_2, tens_2, temp_2, RH_2, AH_2, dev_2, 324.0, mu_2, 0.4985,
                  avge_time_2, avge_dev_2, avge_tens_2, avge_mu_2, avge_temp_2, avge_RH_2, avge_AH_2)

# load second data set for G5c @ 324 Hz (reduced humidity)
time_3 = []
freq_3 = []
tens_3 = []
temp_3 = []
RH_3 = []
AH_3 = []
read_data_file(".\string_test_data_2017.12.13.13.00.csv", time_3, freq_3, tens_3, temp_3, RH_3, AH_3)
# derived responses
dev_3 = []
mu_3 = []                             
avge_time_3 = []
avge_dev_3 = []
avge_tens_3 = []
avge_mu_3 = []
avge_temp_3 = []
avge_RH_3 = []
avge_AH_3 = []
derived_responses(time_3, freq_3, tens_3, temp_3, RH_3, AH_3, dev_3, 324.0, mu_3, 0.4985,
                  avge_time_3, avge_dev_3, avge_tens_3, avge_mu_3, avge_temp_3, avge_RH_3, avge_AH_3)

# load third data set for G5c @ 324 Hz (reduced humidity)
time_4 = []
freq_4 = []
tens_4 = []
temp_4 = []
RH_4 = []
AH_4 = []
read_data_file(".\string_test_data_2017.12.28.09.14.csv", time_4, freq_4, tens_4, temp_4, RH_4, AH_4)
# derived responses
dev_4 = []
mu_4 = []                             
avge_time_4 = []
avge_dev_4 = []
avge_tens_4 = []
avge_mu_4 = []
avge_temp_4 = []
avge_RH_4 = []
avge_AH_4 = []
derived_responses(time_4, freq_4, tens_4, temp_4, RH_4, AH_4, dev_4, 324.0, mu_4, 0.4985,
                  avge_time_4, avge_dev_4, avge_tens_4, avge_mu_4, avge_temp_4, avge_RH_4, avge_AH_4)

# load combined data for G5c
time_2x = []
freq_2x = []
tens_2x = []
temp_2x = []
RH_2x = []
AH_2x = []
read_data_file(".\string_test_data_2017.11.12.10.51.csv", time_2x, freq_2x, tens_2x, temp_2x, RH_2x, AH_2x)
read_data_file(".\string_test_data_2017.12.13.13.00.csv", time_2x, freq_2x, tens_2x, temp_2x, RH_2x, AH_2x)
read_data_file(".\string_test_data_2017.12.28.09.14.csv", time_2x, freq_2x, tens_2x, temp_2x, RH_2x, AH_2x)
# derived responses
dev_2x = []
mu_2x = []                             
avge_time_2x = []
avge_dev_2x = []
avge_tens_2x = []
avge_mu_2x = []
avge_temp_2x = []
avge_RH_2x = []
avge_AH_2x = []
derived_responses(time_2x, freq_2x, tens_2x, temp_2x, RH_2x, AH_2x, dev_2x, 324.0, mu_2x, 0.4985,
                  avge_time_2x, avge_dev_2x, avge_tens_2x, avge_mu_2x, avge_temp_2x, avge_RH_2x, avge_AH_2x)

# load data set for G5c at 235 Hz
time_5 = []
freq_5 = []
tens_5 = []
temp_5 = []
RH_5 = []
AH_5 = []
read_data_file(".\string_test_data_2018.02.20.14.44.csv", time_5, freq_5, tens_5, temp_5, RH_5, AH_5)
# derived responses
dev_5 = []
mu_5 = []                             
avge_time_5 = []
avge_dev_5 = []
avge_tens_5 = []
avge_mu_5 = []
avge_temp_5 = []
avge_RH_5 = []
avge_AH_5 = []
derived_responses(time_5, freq_5, tens_5, temp_5, RH_5, AH_5, dev_5, 235.0, mu_5, 0.4985,
                  avge_time_5, avge_dev_5, avge_tens_5, avge_mu_5, avge_temp_5, avge_RH_5, avge_AH_5)

# load data set for G5c at 288 Hz
time_6 = []
freq_6 = []
tens_6 = []
temp_6 = []
RH_6 = []
AH_6 = []
read_data_file(".\string_test_data_2018.03.19.14.59.csv", time_6, freq_6, tens_6, temp_6, RH_6, AH_6)
# derived responses
dev_6 = []
mu_6 = []                             
avge_time_6 = []
avge_dev_6 = []
avge_tens_6 = []
avge_mu_6 = []
avge_temp_6 = []
avge_RH_6 = []
avge_AH_6 = []
derived_responses(time_6, freq_6, tens_6, temp_6, RH_6, AH_6, dev_6, 288.0, mu_6, 0.4985,
                  avge_time_6, avge_dev_6, avge_tens_6, avge_mu_6, avge_temp_6, avge_RH_6, avge_AH_6)

# load data set for G5c at 306 Hz
time_7 = []
freq_7 = []
tens_7 = []
temp_7 = []
RH_7 = []
AH_7 = []
read_data_file(".\string_test_data_2018.04.09.15.15.csv", time_7, freq_7, tens_7, temp_7, RH_7, AH_7)
read_data_file(".\string_test_data_2018.04.18.09.33.csv", time_7, freq_7, tens_7, temp_7, RH_7, AH_7)
# derived responses
dev_7 = []
mu_7 = []                             
avge_time_7 = []
avge_dev_7 = []
avge_tens_7 = []
avge_mu_7 = []
avge_temp_7 = []
avge_RH_7 = []
avge_AH_7 = []
derived_responses(time_7, freq_7, tens_7, temp_7, RH_7, AH_7, dev_7, 306.0, mu_7, 0.4985,
                  avge_time_7, avge_dev_7, avge_tens_7, avge_mu_7, avge_temp_7, avge_RH_7, avge_AH_7)


# load data set for carbon string C5b at 375 Hz
time_C1 = []
freq_C1 = []
tens_C1 = []
temp_C1 = []
RH_C1 = []
AH_C1 = []
read_data_file(".\string_test_data_2018.03.11.08.52.csv", time_C1, freq_C1, tens_C1, temp_C1, RH_C1, AH_C1)
# derived responses
dev_C1 = []
mu_C1 = []                             
avge_time_C1 = []
avge_dev_C1 = []
avge_tens_C1 = []
avge_mu_C1 = []
avge_temp_C1 = []
avge_RH_C1 = []
avge_AH_C1 = []
derived_responses(time_C1, freq_C1, tens_C1, temp_C1, RH_C1, AH_C1, dev_C1, 375.0, mu_C1, 0.5,
                  avge_time_C1, avge_dev_C1, avge_tens_C1, avge_mu_C1, avge_temp_C1, avge_RH_C1, avge_AH_C1)
```

## Extended test constant length $df/dT$ study¶

In [2]:

```
# study at 324 Hz
plt.figure(figsize=(6,7))

# tuning error vs temperature
ax1=plt.subplot2grid((2,2), (0,0), colspan=2)
ax1.plot(temp_1, dev_1, 'g', lw=1)
ax1.plot(temp_2, dev_2, 'lime', lw=1)
ax1.plot(temp_3, dev_3, 'brown', lw=1)
ax1.plot(temp_4, dev_4, 'brown', lw=1)
ax1.set_xticks(np.arange(5, 40, 5))
ax1.set_xlabel('Temperature ($^\circ$C)')
#ax1.set_yticks(np.arange(5, 40, 5))
ax1.set_ylabel('Tuning error (cent)')

# AH vs temperature
ax2=plt.subplot2grid((2,2), (1,0))
ax2.plot(temp_1, AH_1, 'g', lw=1)
ax2.plot(temp_2, AH_2, 'lime', lw=1)
ax2.plot(temp_3, AH_3, 'brown', lw=1)
ax2.plot(temp_4, AH_4, 'brown', lw=1)
ax2.set_xticks(np.arange(5, 40, 5))
ax2.set_xlabel('Temperature ($^\circ$C)')
#ax2.set_yticks(np.arange(5, 40, 5))
ax2.set_ylabel('Absolute humidity (g/m$^3$)')

# RH vs temperature
ax3=plt.subplot2grid((2,2), (1,1))
ax3.plot(temp_1, RH_1, 'g', lw=1)
ax3.plot(temp_2, RH_2, 'lime', lw=1)
ax3.plot(temp_3, RH_3, 'brown', lw=1)
ax3.plot(temp_4, RH_4, 'brown', lw=1)
ax3.set_xticks(np.arange(5, 40, 5))
ax3.set_xlabel('Temperature ($^\circ$C)')
#ax3.set_yticks(np.arange(5, 40, 5))
ax3.set_ylabel('Relative humidity (%)')

plt.tight_layout()  

# Adjust subplot box sizes
box = ax1.get_position()
ax1.set_position([box.x0, box.y0 - box.height * 0.2, box.width, box.height * 1.2])
box = ax2.get_position()
ax2.set_position([box.x0, box.y0, box.width, box.height * 0.8])
box = ax3.get_position()
ax3.set_position([box.x0, box.y0, box.width, box.height * 0.8])

ax1.text(0.94, 0.93,'(a)', transform=ax1.transAxes)
ax2.text(0.05, 0.9,'(b)', transform=ax2.transAxes)
ax3.text(0.87, 0.9,'(c)', transform=ax3.transAxes)

plt.savefig('./08abc_dfdT_L_ext.eps', format='eps', dpi=1000)
plt.show()
```

In [3]:

```
# study at 235 Hz
plt.figure(figsize=(6,7))

# tuning error vs temperature
ax1=plt.subplot2grid((2,2), (0,0), colspan=2)
ax1.plot(temp_5, dev_5, 'magenta', lw=1)
ax1.set_xticks(np.arange(5, 40, 5))
ax1.set_xlabel('Temperature ($^\circ$C)')
#ax1.set_yticks(np.arange(5, 40, 5))
ax1.set_ylabel('Tuning error (cent)')

# AH vs temperature
ax2=plt.subplot2grid((2,2), (1,0))
ax2.plot(temp_5, AH_5, 'magenta', lw=1)
ax2.set_xticks(np.arange(5, 40, 5))
ax2.set_xlabel('Temperature ($^\circ$C)')
#ax2.set_yticks(np.arange(5, 40, 5))
ax2.set_ylabel('Absolute humidity (g/m$^3$)')

# RH vs temperature
ax3=plt.subplot2grid((2,2), (1,1))
ax3.plot(temp_5, RH_5, 'magenta', lw=1)
ax3.set_xticks(np.arange(5, 40, 5))
ax3.set_xlabel('Temperature ($^\circ$C)')
#ax3.set_yticks(np.arange(5, 40, 5))
ax3.set_ylabel('Relative humidity (%)')

plt.tight_layout()  

# Adjust subplot box sizes
box = ax1.get_position()
ax1.set_position([box.x0, box.y0 - box.height * 0.2, box.width, box.height * 1.2])
box = ax2.get_position()
ax2.set_position([box.x0, box.y0, box.width, box.height * 0.8])
box = ax3.get_position()
ax3.set_position([box.x0, box.y0, box.width, box.height * 0.8])

ax1.text(0.94, 0.93,'(a)', transform=ax1.transAxes)
ax2.text(0.05, 0.9,'(b)', transform=ax2.transAxes)
ax3.text(0.87, 0.9,'(c)', transform=ax3.transAxes)

plt.show()
```

In [4]:

```
# study at 288 Hz
plt.figure(figsize=(6,7))

# tuning error vs temperature
ax1=plt.subplot2grid((2,2), (0,0), colspan=2)
ax1.plot(temp_6, dev_6, 'orange', lw=1)
ax1.set_xticks(np.arange(5, 40, 5))
ax1.set_xlabel('Temperature ($^\circ$C)')
#ax1.set_yticks(np.arange(5, 40, 5))
ax1.set_ylabel('Tuning error (cent)')

# AH vs temperature
ax2=plt.subplot2grid((2,2), (1,0))
ax2.plot(temp_6, AH_6, 'orange', lw=1)
ax2.set_xticks(np.arange(5, 40, 5))
ax2.set_xlabel('Temperature ($^\circ$C)')
#ax2.set_yticks(np.arange(5, 40, 5))
ax2.set_ylabel('Absolute humidity (g/m$^3$)')

# RH vs temperature
ax3=plt.subplot2grid((2,2), (1,1))
ax3.plot(temp_6, RH_6, 'orange', lw=1)
ax3.set_xticks(np.arange(5, 40, 5))
ax3.set_xlabel('Temperature ($^\circ$C)')
#ax3.set_yticks(np.arange(5, 40, 5))
ax3.set_ylabel('Relative humidity (%)')

plt.tight_layout()  

# Adjust subplot box sizes
box = ax1.get_position()
ax1.set_position([box.x0, box.y0 - box.height * 0.2, box.width, box.height * 1.2])
box = ax2.get_position()
ax2.set_position([box.x0, box.y0, box.width, box.height * 0.8])
box = ax3.get_position()
ax3.set_position([box.x0, box.y0, box.width, box.height * 0.8])

ax1.text(0.94, 0.93,'(a)', transform=ax1.transAxes)
ax2.text(0.05, 0.9,'(b)', transform=ax2.transAxes)
ax3.text(0.87, 0.9,'(c)', transform=ax3.transAxes)

plt.show()
```

In [5]:

```
# study at 306 Hz
plt.figure(figsize=(6,7))

# tuning error vs temperature
ax1=plt.subplot2grid((2,2), (0,0), colspan=2)
ax1.plot(temp_7, dev_7, 'red', lw=1)
ax1.set_xticks(np.arange(5, 40, 5))
ax1.set_xlabel('Temperature ($^\circ$C)')
#ax1.set_yticks(np.arange(5, 40, 5))
ax1.set_ylabel('Tuning error (cent)')

# AH vs temperature
ax2=plt.subplot2grid((2,2), (1,0))
ax2.plot(temp_7, AH_7, 'red', lw=1)
ax2.set_xticks(np.arange(5, 40, 5))
ax2.set_xlabel('Temperature ($^\circ$C)')
#ax2.set_yticks(np.arange(5, 40, 5))
ax2.set_ylabel('Absolute humidity (g/m$^3$)')

# RH vs temperature
ax3=plt.subplot2grid((2,2), (1,1))
ax3.plot(temp_7, RH_7, 'red', lw=1)
ax3.set_xticks(np.arange(5, 40, 5))
ax3.set_xlabel('Temperature ($^\circ$C)')
#ax3.set_yticks(np.arange(5, 40, 5))
ax3.set_ylabel('Relative humidity (%)')

plt.tight_layout()  

# Adjust subplot box sizes
box = ax1.get_position()
ax1.set_position([box.x0, box.y0 - box.height * 0.2, box.width, box.height * 1.2])
box = ax2.get_position()
ax2.set_position([box.x0, box.y0, box.width, box.height * 0.8])
box = ax3.get_position()
ax3.set_position([box.x0, box.y0, box.width, box.height * 0.8])

ax1.text(0.94, 0.93,'(a)', transform=ax1.transAxes)
ax2.text(0.05, 0.9,'(b)', transform=ax2.transAxes)
ax3.text(0.87, 0.9,'(c)', transform=ax3.transAxes)

plt.show()
```

## Tension and linear density variations in G5c @ 324 Hz¶

In [6]:

```
index1 = 399
index2 = 861
index3 = 1249

index4 = locate_time_index(avge_time_2, time_2[index3] - 0.5, 0)

# plot
fig=plt.figure(figsize=(9,5.5))
#
# frquency deviation vs. temperature
ax1=fig.add_subplot(231)
ax1.plot(temp_2[:index1+1], dev_2[:index1+1], 'b')
ax1.plot(temp_2[index1:index2+1], dev_2[index1:index2+1], 'g')
ax1.plot(temp_2[index2:index3+1], dev_2[index2:index3+1], 'cyan')
ax1.set_xticks(np.arange(10, 40, 5))
ax1.set_xlabel('Temperature ($^\circ$C)')
ax1.set_ylabel('Frequency deviation (cent)')

# tension vs. temperature
ax2=fig.add_subplot(232)
ax2.plot(temp_2[:index1+1], tens_2[:index1+1], 'b')
ax2.plot(temp_2[index1:index2+1], tens_2[index1:index2+1], 'g')
ax2.plot(temp_2[index2:index3+1], tens_2[index2:index3+1], 'cyan')
ax2.set_xticks(np.arange(10, 40, 5))
ax2.set_yticks(np.arange(255, 265, 1))
ax2.set_xlabel('Temperature ($^\circ$C)')
ax2.set_ylabel('Tension (N)')

# linear density vs. temperature
ax3=fig.add_subplot(233)
ax3.plot(temp_2[:index1+1], mu_2[:index1+1], 'b')
ax3.plot(temp_2[index1:index2+1], mu_2[index1:index2+1], 'g')
ax3.plot(temp_2[index2:index3+1], mu_2[index2:index3+1], 'cyan')
ax3.set_xticks(np.arange(10, 40, 5))
ax3.set_xlabel('Temperature ($^\circ$C)')
ax3.set_ylabel('Linear density (g/m$^3$)')

# temperature vs. time
ax4=fig.add_subplot(234)
ax4.plot(avge_time_2[:index4+1], avge_temp_2[:index4+1], 'k')
ax4.plot(time_2[:index1+1], temp_2[:index1+1], 'b')
ax4.plot(time_2[index1:index2+1], temp_2[index1:index2+1], 'g')
ax4.plot(time_2[index2:index3+1], temp_2[index2:index3+1], 'cyan')
ax4.set_xticks(np.arange(0, 6, 1))
ax4.set_xlabel('Time (days)')
ax4.set_ylabel('Temperature ($^\circ$C)')

# absolute humidity vs. time
ax5=fig.add_subplot(235)
ax5.plot(avge_time_2[:index4+1], avge_AH_2[:index4+1], 'k')
ax5.plot(time_2[:index1+1], AH_2[:index1+1], 'b')
ax5.plot(time_2[index1:index2+1], AH_2[index1:index2+1], 'g')
ax5.plot(time_2[index2:index3+1], AH_2[index2:index3+1], 'cyan')
ax5.set_xticks(np.arange(0, 6, 1))
ax5.set_xlabel('Time (days)')
ax5.set_ylabel('Absolute humidity (g/m$^3$)')

# relative humidity vs. time
ax6=fig.add_subplot(236)
ax6.plot(avge_time_2[:index4+1], avge_RH_2[:index4+1], 'k')
ax6.plot(time_2[:index1+1], RH_2[:index1+1], 'b')
ax6.plot(time_2[index1:index2+1], RH_2[index1:index2+1], 'g')
ax6.plot(time_2[index2:index3+1], RH_2[index2:index3+1], 'cyan')
ax6.set_xticks(np.arange(0, 6, 1))
ax6.set_xlabel('Time (days)')
ax6.set_ylabel('Relative humidity (%)')

plt.tight_layout()  

ax1.text(0.87, 0.9,'(a)', transform=ax1.transAxes)
ax2.text(0.87, 0.9,'(b)', transform=ax2.transAxes)
ax3.text(0.05, 0.9,'(c)', transform=ax3.transAxes)
ax4.text(0.87, 0.9,'(d)', transform=ax4.transAxes)
ax5.text(0.87, 0.9,'(e)', transform=ax5.transAxes)
ax6.text(0.87, 0.9,'(f)', transform=ax6.transAxes)

plt.savefig('./12abcdef_dFdT_variation.eps', format='eps', dpi=1000)
plt.show()
```

## Extended test thermal sensitivity vs. average temperature and humidity¶

In [7]:

```
# process data
order = 2  # 1: line; 2: quadratic

# process data for G5b at 324 Hz
fitF_1 = []
dFdT_1 = []
midtime_1 = []
avgT_1 = []
avgAH_1 = []
avgRH_1 = []
avgF_1 = []
mmtime_1 = []
mmtemp_1 = []
process_data_file(time_1, temp_1, AH_1, RH_1, tens_1, tens_1, 1.5, fitF_1, order, dFdT_1, 
                  midtime_1, avgT_1, avgAH_1, avgRH_1, avgF_1, mmtime_1, mmtemp_1)
fitmu_1 = []
dmudT_1 = []
midtime_1 = []
avgT_1 = []
avgAH_1 = []
avgRH_1 = []
avgF_1 = []
mmtime_1 = []
mmtemp_1 = []
process_data_file(time_1, temp_1, AH_1, RH_1, tens_1, mu_1, 1.5, fitmu_1, order, dmudT_1, 
                  midtime_1, avgT_1, avgAH_1, avgRH_1, avgF_1, mmtime_1, mmtemp_1)


# process data for G5c at 324 Hz
fitF_2 = []
dFdT_2 = []
midtime_2 = []
avgT_2 = []
avgAH_2 = []
avgRH_2 = []
avgF_2 = []
mmtime_2 = []
mmtemp_2 = []
process_data_file(time_2, temp_2, AH_2, RH_2, tens_2, tens_2, 1.5, fitF_2, order, dFdT_2,
                  midtime_2, avgT_2, avgAH_2, avgRH_2, avgF_2, mmtime_2, mmtemp_2)
fitmu_2 = []
dmudT_2 = []
midtime_2 = []
avgT_2 = []
avgAH_2 = []
avgRH_2 = []
avgF_2 = []
mmtime_2 = []
mmtemp_2 = []
process_data_file(time_2, temp_2, AH_2, RH_2, tens_2, mu_2, 1.5, fitmu_2, order, dmudT_2,
                  midtime_2, avgT_2, avgAH_2, avgRH_2, avgF_2, mmtime_2, mmtemp_2)
#
# process second data set for G5c at 324 Hz (reduced humidity)
time_point = 4.4 # days
index = locate_time_index(time_3, time_point, 0)
fitF_3 = []
dFdT_3 = []
midtime_3 = []
avgT_3 = []
avgAH_3 = []
avgRH_3 = []
avgF_3 = []
mmtime_3 = []
mmtemp_3 = []
process_data_file(time_3[index:], temp_3[index:], AH_3[index:], RH_3[index:], tens_3[index:], tens_3[index:], 1.5,
                  fitF_3, order, dFdT_3, midtime_3, avgT_3, avgAH_3, avgRH_3, avgF_3, mmtime_3, mmtemp_3)
fitmu_3 = []
dmudT_3 = []
midtime_3 = []
avgT_3 = []
avgAH_3 = []
avgRH_3 = []
avgF_3 = []
mmtime_3 = []
mmtemp_3 = []
process_data_file(time_3[index:], temp_3[index:], AH_3[index:], RH_3[index:], tens_3[index:], mu_3[index:], 1.5,
                  fitmu_3, order, dmudT_3, midtime_3, avgT_3, avgAH_3, avgRH_3, avgF_3, mmtime_3, mmtemp_3)
#
# process third data set for G5c at 324 Hz (reduced humidity)
# first section
time_point = 5.5 # days
index = locate_time_index(time_4, time_point, 0)
fitF_4a = []
dFdT_4a = []
midtime_4a = []
avgT_4a = []
avgAH_4a = []
avgRH_4a = []
avgF_4a = []
mmtime_4a = []
mmtemp_4a = []
process_data_file(time_4[:index], temp_4[:index], AH_4[:index], RH_4[:index], tens_4[:index], tens_4[:index], 1.0,
                  fitF_4a, order, dFdT_4a, midtime_4a, avgT_4a, avgAH_4a, avgRH_4a, avgF_4a, mmtime_4a, mmtemp_4a)
fitmu_4a = []
dmudT_4a = []
midtime_4a = []
avgT_4a = []
avgAH_4a = []
avgRH_4a = []
avgF_4a = []
mmtime_4a = []
mmtemp_4a = []
process_data_file(time_4[:index], temp_4[:index], AH_4[:index], RH_4[:index], tens_4[:index], mu_4[:index], 1.0,
                  fitmu_4a, order, dmudT_4a, midtime_4a, avgT_4a, avgAH_4a, avgRH_4a, avgF_4a, mmtime_4a, mmtemp_4a)
# second section
time_point = 5.5 # days
index1 = locate_time_index(time_4, time_point, 0)
time_point = 12 # days
index2 = locate_time_index(time_4, time_point, 0)
fitF_4b = []
dFdT_4b = []
midtime_4b = []
avgT_4b = []
avgAH_4b = []
avgRH_4b = []
avgF_4b = []
mmtime_4b = []
mmtemp_4b = []
process_data_file(time_4[index1:index2], temp_4[index1:index2], AH_4[index1:index2], RH_4[index1:index2], 
                  tens_4[index1:index2], tens_4[index1:index2], 1.0,
                  fitF_4b, order, dFdT_4b, midtime_4b, avgT_4b, avgAH_4b, avgRH_4b, avgF_4b, mmtime_4b, mmtemp_4b)
fitmu_4b = []
dmudT_4b = []
midtime_4b = []
avgT_4b = []
avgAH_4b = []
avgRH_4b = []
avgF_4b = []
mmtime_4b = []
mmtemp_4b = []
process_data_file(time_4[index1:index2], temp_4[index1:index2], AH_4[index1:index2], RH_4[index1:index2], 
                  tens_4[index1:index2], mu_4[index1:index2], 1.0,
                  fitmu_4b, order, dmudT_4b, midtime_4b, avgT_4b, avgAH_4b, avgRH_4b, avgF_4b, mmtime_4b, mmtemp_4b)
# third section
time_point = 12.5 # days
index = locate_time_index(time_4, time_point, 0)
fitF_4c = []
dFdT_4c = []
midtime_4c = []
avgT_4c = []
avgAH_4c = []
avgRH_4c = []
avgF_4c = []
mmtime_4c = []
mmtemp_4c = []
process_data_file(time_4[index:], temp_4[index:], AH_4[index:], RH_4[index:], tens_4[index:], tens_4[index:], 1.0,
                  fitF_4c, order, dFdT_4c, midtime_4c, avgT_4c, avgAH_4c, avgRH_4c, avgF_4c, mmtime_4c, mmtemp_4c)
fitmu_4c = []
dmudT_4c = []
midtime_4c = []
avgT_4c = []
avgAH_4c = []
avgRH_4c = []
avgF_4c = []
mmtime_4c = []
mmtemp_4c = []
process_data_file(time_4[index:], temp_4[index:], AH_4[index:], RH_4[index:], tens_4[index:], mu_4[index:], 1.0,
                  fitmu_4c, order, dmudT_4c, midtime_4c, avgT_4c, avgAH_4c, avgRH_4c, avgF_4c, mmtime_4c, mmtemp_4c)
# combined responses
dFdT_4 = np.concatenate([dFdT_4a, dFdT_4b, dFdT_4c])
dmudT_4 = np.concatenate([dmudT_4a, dmudT_4b, dmudT_4c])
avgT_4 = np.concatenate([avgT_4a, avgT_4b, avgT_4c])
avgAH_4 = np.concatenate([avgAH_4a, avgAH_4b, avgAH_4c])
avgRH_4 = np.concatenate([avgRH_4a, avgRH_4b, avgRH_4c])


# process data for G5c at 235 Hz
# first section
time_point = 8 # days
index = locate_time_index(time_5, time_point, 0)
fitF_5a = []
dFdT_5a = []
midtime_5a = []
avgT_5a = []
avgAH_5a = []
avgRH_5a = []
avgF_5a = []
mmtime_5a = []
mmtemp_5a = []
process_data_file(time_5[:index], temp_5[:index], AH_5[:index], RH_5[:index], tens_5[:index], tens_5[:index], 1.0,
                  fitF_5a, order, dFdT_5a, midtime_5a, avgT_5a, avgAH_5a, avgRH_5a, avgF_5a, mmtime_5a, mmtemp_5a)
fitmu_5a = []
dmudT_5a = []
midtime_5a = []
avgT_5a = []
avgAH_5a = []
avgRH_5a = []
avgF_5a = []
mmtime_5a = []
mmtemp_5a = []
process_data_file(time_5[:index], temp_5[:index], AH_5[:index], RH_5[:index], tens_5[:index], mu_5[:index], 1.0,
                  fitmu_5a, order, dmudT_5a, midtime_5a, avgT_5a, avgAH_5a, avgRH_5a, avgF_5a, mmtime_5a, mmtemp_5a)
# second section
time_point = 7 # days
index1 = locate_time_index(time_5, time_point, 0)
time_point = 11 # days
index2 = locate_time_index(time_5, time_point, 0)
fitF_5b = []
dFdT_5b = []
midtime_5b = []
avgT_5b = []
avgAH_5b = []
avgRH_5b = []
avgF_5b = []
mmtime_5b = []
mmtemp_5b = []
process_data_file(time_5[index1:index2], temp_5[index1:index2], AH_5[index1:index2], RH_5[index1:index2], 
                  tens_5[index1:index2], tens_5[index1:index2], 1.0,
                  fitF_5b, order, dFdT_5b, midtime_5b, avgT_5b, avgAH_5b, avgRH_5b, avgF_5b, mmtime_5b, mmtemp_5b)
fitmu_5b = []
dmudT_5b = []
midtime_5b = []
avgT_5b = []
avgAH_5b = []
avgRH_5b = []
avgF_5b = []
mmtime_5b = []
mmtemp_5b = []
process_data_file(time_5[index1:index2], temp_5[index1:index2], AH_5[index1:index2], RH_5[index1:index2], 
                  tens_5[index1:index2], mu_5[index1:index2], 1.0,
                  fitmu_5b, order, dmudT_5b, midtime_5b, avgT_5b, avgAH_5b, avgRH_5b, avgF_5b, mmtime_5b, mmtemp_5b)
# third section
time_point = 10 # days
index1 = locate_time_index(time_5, time_point, 0)
time_point = 14 # days
index2 = locate_time_index(time_5, time_point, 0)
fitF_5c = []
dFdT_5c = []
midtime_5c = []
avgT_5c = []
avgAH_5c = []
avgRH_5c = []
avgF_5c = []
mmtime_5c = []
mmtemp_5c = []
process_data_file(time_5[index1:index2], temp_5[index1:index2], AH_5[index1:index2], RH_5[index1:index2], 
                  tens_5[index1:index2], tens_5[index1:index2], 1.0,
                  fitF_5c, order, dFdT_5c, midtime_5c, avgT_5c, avgAH_5c, avgRH_5c, avgF_5c, mmtime_5c, mmtemp_5c)
fitmu_5c = []
dmudT_5c = []
midtime_5c = []
avgT_5c = []
avgAH_5c = []
avgRH_5c = []
avgF_5c = []
mmtime_5c = []
mmtemp_5c = []
process_data_file(time_5[index1:index2], temp_5[index1:index2], AH_5[index1:index2], RH_5[index1:index2], 
                  tens_5[index1:index2], mu_5[index1:index2], 1.0,
                  fitmu_5c, order, dmudT_5c, midtime_5c, avgT_5c, avgAH_5c, avgRH_5c, avgF_5c, mmtime_5c, mmtemp_5c)
# fourth section
time_point = 13 # days
index = locate_time_index(time_5, time_point, 0)
fitF_5d = []
dFdT_5d = []
midtime_5d = []
avgT_5d = []
avgAH_5d = []
avgRH_5d = []
avgF_5d = []
mmtime_5d = []
mmtemp_5d = []
process_data_file(time_5[index:], temp_5[index:], AH_5[index:], RH_5[index:], tens_5[index:], tens_5[index:], 1.0,
                  fitF_5d, order, dFdT_5d, midtime_5d, avgT_5d, avgAH_5d, avgRH_5d, avgF_5d, mmtime_5d, mmtemp_5d)
fitmu_5d = []
dmudT_5d = []
midtime_5d = []
avgT_5d = []
avgAH_5d = []
avgRH_5d = []
avgF_5d = []
mmtime_5d = []
mmtemp_5d = []
process_data_file(time_5[index:], temp_5[index:], AH_5[index:], RH_5[index:], tens_5[index:], mu_5[index:], 1.0,
                  fitmu_5d, order, dmudT_5d, midtime_5d, avgT_5d, avgAH_5d, avgRH_5d, avgF_5d, mmtime_5d, mmtemp_5d)
# combined responses
dFdT_5 = np.concatenate([dFdT_5a, dFdT_5b, dFdT_5c, dFdT_5d])
dmudT_5 = np.concatenate([dmudT_5a, dmudT_5b, dmudT_5c, dmudT_5d])
avgT_5 = np.concatenate([avgT_5a, avgT_5b, avgT_5c, avgT_5d])
avgAH_5 = np.concatenate([avgAH_5a, avgAH_5b, avgAH_5c, avgAH_5d])
avgRH_5 = np.concatenate([avgRH_5a, avgRH_5b, avgRH_5c, avgRH_5d])


# process data for G5c at 288 Hz
fitF_6 = []
dFdT_6 = []
midtime_6 = []
avgT_6 = []
avgAH_6 = []
avgRH_6 = []
avgF_6 = []
mmtime_6 = []
mmtemp_6 = []
process_data_file(time_6, temp_6, AH_6, RH_6, tens_6, tens_6, 1.0, fitF_6, order, dFdT_6,
                  midtime_6, avgT_6, avgAH_6, avgRH_6, avgF_6, mmtime_6, mmtemp_6)
fitmu_6 = []
dmudT_6 = []
midtime_6 = []
avgT_6 = []
avgAH_6 = []
avgRH_6 = []
avgF_6 = []
mmtime_6 = []
mmtemp_6 = []
process_data_file(time_6, temp_6, AH_6, RH_6, tens_6, mu_6, 1.0, fitmu_6, order, dmudT_6,
                  midtime_6, avgT_6, avgAH_6, avgRH_6, avgF_6, mmtime_6, mmtemp_6)


# process data for G5c at 306 Hz
fitF_7 = []
dFdT_7 = []
midtime_7 = []
avgT_7 = []
avgAH_7 = []
avgRH_7 = []
avgF_7 = []
mmtime_7 = []
mmtemp_7 = []
process_data_file(time_7, temp_7, AH_7, RH_7, tens_7, tens_7, 1.0, fitF_7, order, dFdT_7,
                  midtime_7, avgT_7, avgAH_7, avgRH_7, avgF_7, mmtime_7, mmtemp_7)
fitmu_7 = []
dmudT_7 = []
midtime_7 = []
avgT_7 = []
avgAH_7 = []
avgRH_7 = []
avgF_7 = []
mmtime_7 = []
mmtemp_7 = []
process_data_file(time_7, temp_7, AH_7, RH_7, tens_7, mu_7, 1.0, fitmu_7, order, dmudT_7,
                  midtime_7, avgT_7, avgAH_7, avgRH_7, avgF_7, mmtime_7, mmtemp_7)


# process data for carbon string C5b at 375 Hz
fitF_C1 = []
dFdT_C1 = []
midtime_C1 = []
avgT_C1 = []
avgAH_C1 = []
avgRH_C1 = []
avgF_C1 = []
mmtime_C1 = []
mmtemp_C1 = []
process_data_file(time_C1, temp_C1, AH_C1, RH_C1, tens_C1, tens_C1, 1.0, fitF_C1, order, dFdT_C1,
                  midtime_C1, avgT_C1, avgAH_C1, avgRH_C1, avgF_C1, mmtime_C1, mmtemp_C1)
fitmu_C1 = []
dmudT_C1 = []
midtime_C1 = []
avgT_C1 = []
avgAH_C1 = []
avgRH_C1 = []
avgF_C1 = []
mmtime_C1 = []
mmtemp_C1 = []
process_data_file(time_C1, temp_C1, AH_C1, RH_C1, tens_C1, mu_C1, 1.0, fitmu_C1, order, dmudT_C1,
                  midtime_C1, avgT_C1, avgAH_C1, avgRH_C1, avgF_C1, mmtime_C1, mmtemp_C1)


# plot max min points
fig=plt.figure(figsize=(12,12))
#
ax1=fig.add_subplot(811)
ax1.plot(time_1, temp_1, 'g')
ax1.plot(mmtime_1, mmtemp_1, 'ko', markersize = 6)
ax1.set_xlabel('Time (days)')
ax1.set_ylabel('Temperature ($^\circ$C)')
#
ax2=fig.add_subplot(812)
ax2.plot(time_2, temp_2, 'lime')
ax2.plot(mmtime_2, mmtemp_2, 'ko', markersize = 6)
ax2.set_xlabel('Time (days)')
ax2.set_ylabel('Temperature ($^\circ$C)')
#
ax3=fig.add_subplot(813)
ax3.plot(time_3, temp_3, 'brown')
ax3.plot(mmtime_3, mmtemp_3, 'ko', markersize = 6)
ax3.set_xlabel('Time (days)')
ax3.set_ylabel('Temperature ($^\circ$C)')
#
ax4=fig.add_subplot(814)
ax4.plot(time_4, temp_4, 'r')
ax4.plot(mmtime_4a, mmtemp_4a, 'ko', markersize = 6)
ax4.plot(mmtime_4b, mmtemp_4b, 'bo', markersize = 6)
ax4.plot(mmtime_4c, mmtemp_4c, 'ko', markersize = 6)
ax4.set_xlabel('Time (days)')
ax4.set_ylabel('Temperature ($^\circ$C)')
#
ax5=fig.add_subplot(815)
ax5.plot(time_5, temp_5, 'magenta')
ax5.plot(mmtime_5a, mmtemp_5a, 'ko', markersize = 6)
ax5.plot(mmtime_5b, mmtemp_5b, 'bo', markersize = 6)
ax5.plot(mmtime_5c, mmtemp_5c, 'ko', markersize = 6)
ax5.plot(mmtime_5d, mmtemp_5d, 'bo', markersize = 6)
ax5.set_xlabel('Time (days)')
ax5.set_ylabel('Temperature ($^\circ$C)')
#
ax6=fig.add_subplot(816)
ax6.plot(time_6, temp_6, 'orange')
ax6.plot(mmtime_6, mmtemp_6, 'ko', markersize = 6)
ax6.set_xlabel('Time (days)')
ax6.set_ylabel('Temperature ($^\circ$C)')
#
ax6=fig.add_subplot(817)
ax6.plot(time_7, temp_7, 'red')
ax6.plot(mmtime_7, mmtemp_7, 'ko', markersize = 6)
ax6.set_xlabel('Time (days)')
ax6.set_ylabel('Temperature ($^\circ$C)')
#
ax9=fig.add_subplot(818)
ax9.plot(time_C1, temp_C1, 'black')
ax9.plot(mmtime_C1, mmtemp_C1, 'ko', markersize = 6)
ax9.set_xlabel('Time (days)')
ax9.set_ylabel('Temperature ($^\circ$C)')
#
fig.tight_layout()  
#plt.show()
```

In [8]:

```
# plots for G5b and G5c

mkr='o'
msz=2

# plot dy/dT vs avge RH
fig4=plt.figure(figsize=(6,2.75))
ax41=fig4.add_subplot(121)
ax42=fig4.add_subplot(122)
#
# dF/dT
ax41.plot(avgRH_1, dFdT_1, 'g', ls='none', marker=mkr, ms=msz)
ax41.plot(avgRH_2, dFdT_2, 'lime', ls='none', marker=mkr, ms=msz)
ax41.plot(avgRH_3, dFdT_3, 'brown', ls='none', marker=mkr, ms=msz)
ax41.plot(avgRH_4, dFdT_4, 'brown', ls='none', marker=mkr, ms=msz)
ax41.plot(avgRH_5, dFdT_5, 'magenta', ls='none', marker=mkr, ms=msz)
ax41.plot(avgRH_6, dFdT_6, 'orange', ls='none', marker=mkr, ms=msz)
ax41.plot(avgRH_7, dFdT_7, 'red', ls='none', marker=mkr, ms=msz)
#ax41.plot(avgRH_C1, dFdT_C1, 'black', ls='none', marker=mkr, ms=msz)
ax41.set_xticks(np.arange(50, 80, 5))
ax41.set_xlabel('Average RH (%)')
ax41.set_ylabel('dF/dT (N/$^\circ$C)')
#
# dy/dT
ax42.plot(avgRH_1, dmudT_1, 'g', ls='none', marker=mkr, ms=msz)
ax42.plot(avgRH_2, dmudT_2, 'lime', ls='none', marker=mkr, ms=msz)
ax42.plot(avgRH_3, dmudT_3, 'brown', ls='none', marker=mkr, ms=msz)
ax42.plot(avgRH_4, dmudT_4, 'brown', ls='none', marker=mkr, ms=msz)
ax42.plot(avgRH_5, dmudT_5, 'magenta', ls='none', marker=mkr, ms=msz)
ax42.plot(avgRH_6, dmudT_6, 'orange', ls='none', marker=mkr, ms=msz)
ax42.plot(avgRH_7, dmudT_7, 'red', ls='none', marker=mkr, ms=msz)
#ax42.plot(avgRH_C1, dmudT_C1, 'black', ls='none', marker=mkr, ms=msz)
ax42.set_xticks(np.arange(50, 80, 5))
ax42.set_xlabel('Average RH (%)')
ax42.set_ylabel('d$\mu$/dT (g/m$^\circ$C)')
#
fig4.tight_layout()  


# tension and linear density versus temperature
fig5=plt.figure(figsize=(8,4.5))
ax1=fig5.add_subplot(121)
ax2=fig5.add_subplot(122)
pls='-'
plw=0.5
#
# tension vs temperature
ax1.plot(temp_1, tens_1, 'g', ls=pls, lw=plw)
ax1.plot(temp_2, tens_2, 'lime', ls=pls, lw=plw)
ax1.plot(temp_3, tens_3, 'brown', ls=pls, lw=plw)
ax1.plot(temp_4, tens_4, 'brown', ls=pls, lw=plw)
ax1.plot(temp_5, tens_5, 'magenta', ls=pls, lw=plw)
ax1.plot(temp_6, tens_6, 'orange', ls=pls, lw=plw)
ax1.plot(temp_7, tens_7, 'red', ls=pls, lw=plw)
#ax1.plot(temp_C1, tens_C1, 'k', ls=pls, lw=plw)
ax1.set_xticks(np.arange(5, 40, 5))
ax1.set_xlabel('Temperature ($^\circ$C)')
#ax1.set_yticks(np.arange(5, 40, 5))
ax1.set_ylabel('Tension (N)')
#
# linear density vs temperature
ax2.plot(temp_1, mu_1, 'g', ls=pls, lw=plw, label='G5b @ 324 Hz')
ax2.plot(temp_2, mu_2, 'lime', ls=pls, lw=plw, label='G5c @ 324 Hz')
ax2.plot(temp_3, mu_3, 'brown', ls=pls, lw=plw, label='G5c @ 324 Hz, reduced RH')
ax2.plot(temp_4, mu_4, 'brown', ls=pls, lw=plw)
ax2.plot(temp_5, mu_5, 'magenta', ls=pls, lw=plw, label='G5c @ 235 Hz')
ax2.plot(temp_6, mu_6, 'orange', ls=pls, lw=plw, label='G5c @ 288 Hz')
ax2.plot(temp_7, mu_7, 'red', ls=pls, lw=plw, label='G5c @ 306 Hz')
#ax2.plot(temp_C1, mu_C1, 'k', ls=pls, lw=plw, label='C5b @ 375 Hz')
ax2.set_xticks(np.arange(5, 40, 5))
ax2.set_xlabel('Temperature ($^\circ$C)')
#ax2.set_yticks(np.arange(5, 40, 5))
ax2.set_ylabel('Linear density (g/m)')
# 
fig5.tight_layout()  # need to do this before adjusting box heights  
#
# Shrink subplot box heights by 12% on the bottom
factor = 0.12
box = ax1.get_position()
ax1.set_position([box.x0, box.y0 + box.height * factor, box.width, box.height * (1-factor)])
box = ax2.get_position()
ax2.set_position([box.x0, box.y0 + box.height * factor, box.width, box.height * (1-factor)])
#
ax1.text(0.05, 0.9,'(a)', transform=ax1.transAxes)
ax2.text(0.87, 0.9,'(b)', transform=ax2.transAxes)
ax2.legend(bbox_to_anchor=(-0.17, -0.17), loc=9, ncol=3, borderaxespad=0.)
#
fig5.savefig('./13ab_long_F_and_mu.eps', format='eps', dpi=1000)
plt.show()


# dF/dT and d(mu)/dT responses
fig6=plt.figure(figsize=(6,5.5))
ax2=fig6.add_subplot(221)
ax3=fig6.add_subplot(222)
ax5=fig6.add_subplot(223)
ax6=fig6.add_subplot(224)
#
# dF/dT vs avge temperature
ax2.plot([0,300],[0,0],'k--',lw=1)  # Zero reference
ax2.plot(avgT_1, dFdT_1, 'g', ls='none', marker=mkr, ms=msz)
ax2.plot(avgT_2, dFdT_2, 'lime', ls='none', marker=mkr, ms=msz)
ax2.plot(avgT_3, dFdT_3, 'brown', ls='none', marker=mkr, ms=msz)
ax2.plot(avgT_4, dFdT_4, 'brown', ls='none', marker=mkr, ms=msz)
ax2.plot(avgT_5, dFdT_5, 'magenta', ls='none', marker=mkr, ms=msz)
ax2.plot(avgT_6, dFdT_6, 'orange', ls='none', marker=mkr, ms=msz)
ax2.plot(avgT_7, dFdT_7, 'red', ls='none', marker=mkr, ms=msz)
#ax2.plot(avgT_C1, dFdT_C1, 'k', ls='none', marker=mkr, ms=msz)
ax2.axis([3,30,-1.2,4.2])
ax2.set_xticks(np.arange(5, 35, 5))
ax2.set_xlabel('Average temperature ($^\circ$C)')
ax2.set_ylabel('dF/dT (N/$^\circ$C)')
#   
# dF/dT vs avge AH
ax3.plot([0,300],[0,0],'k--',lw=1)  # Zero reference
ax3.plot(avgAH_1, dFdT_1, 'g', ls='none', marker=mkr, ms=msz)
ax3.plot(avgAH_2, dFdT_2, 'lime', ls='none', marker=mkr, ms=msz)
ax3.plot(avgAH_3, dFdT_3, 'brown', ls='none', marker=mkr, ms=msz)
ax3.plot(avgAH_4, dFdT_4, 'brown', ls='none', marker=mkr, ms=msz)
ax3.plot(avgAH_5, dFdT_5, 'magenta', ls='none', marker=mkr, ms=msz)
ax3.plot(avgAH_6, dFdT_6, 'orange', ls='none', marker=mkr, ms=msz)
ax3.plot(avgAH_7, dFdT_7, 'red', ls='none', marker=mkr, ms=msz)
#ax3.plot(avgAH_C1, dFdT_C1, 'k', ls='none', marker=mkr, ms=msz)
ax3.axis([3,20,-1.2,4.2])
ax3.set_xticks(np.arange(5, 25, 5))
ax3.set_xlabel('Average AH (g/m$^3$)')
ax3.set_ylabel('dF/dT (N/$^\circ$C)')
#
# dmu/dT vs avge temperature
ax5.plot([0,300],[0,0],'k--',lw=1)  # Zero reference
ax5.plot(avgT_1, dmudT_1, 'g', ls='none', marker=mkr, ms=msz)
ax5.plot(avgT_2, dmudT_2, 'lime', ls='none', marker=mkr, ms=msz)
ax5.plot(avgT_3, dmudT_3, 'brown', ls='none', marker=mkr, ms=msz)
ax5.plot(avgT_4, dmudT_4, 'brown', ls='none', marker=mkr, ms=msz)
ax5.plot(avgT_5, dmudT_5, 'magenta', ls='none', marker=mkr, ms=msz)
ax5.plot(avgT_6, dmudT_6, 'orange', ls='none', marker=mkr, ms=msz)
ax5.plot(avgT_7, dmudT_7, 'red', ls='none', marker=mkr, ms=msz)
#ax5.plot(avgT_C1, dmudT_C1, 'k', ls='none', marker=mkr, ms=msz)
ax5.axis([3,30,-0.011,0.045])
ax5.set_xticks(np.arange(5, 35, 5))
ax5.set_xlabel('Average temperature ($^\circ$C)')
ax5.set_ylabel('d$\mu$/dT (g/m$^\circ$C)')
#    
# dmu/dT vs avge AH
ax6.plot([0,300],[0,0],'k--',lw=1)  # Zero reference
ax6.plot(avgAH_1, dmudT_1, 'g', ls='none', marker=mkr, ms=msz, label='G5b @ 324 Hz')
ax6.plot(avgAH_2, dmudT_2, 'lime', ls='none', marker=mkr, ms=msz, label='G5c @ 324 Hz')
ax6.plot(avgAH_3, dmudT_3, 'brown', ls='none', marker=mkr, ms=msz, label='G5c @ 324 Hz, reduced RH')
ax6.plot(avgAH_4, dmudT_4, 'brown', ls='none', marker=mkr, ms=msz)
ax6.plot(avgAH_5, dmudT_5, 'magenta', ls='none', marker=mkr, ms=msz, label='G5c @ 235 Hz')
ax6.plot(avgAH_6, dmudT_6, 'orange', ls='none', marker=mkr, ms=msz, label='G5c @ 288 Hz')
ax6.plot(avgAH_7, dmudT_7, 'red', ls='none', marker=mkr, ms=msz, label='G5c @ 306 Hz')
#ax6.plot(avgAH_C1, dmudT_C1, 'k', ls='none', marker=mkr, ms=msz, label='C5b @ 375 Hz')
ax6.axis([3,20,-0.011,0.045])
ax6.set_xticks(np.arange(5, 25, 5))
ax6.set_xlabel('Average AH (g/m$^3$)')
ax6.set_ylabel('d$\mu$/dT (g/m$^\circ$C)')
#    
fig6.tight_layout()  # need to do this before adjusting box heights  
#
# Shrink subplot box heights by 10% on the bottom
box = ax2.get_position()
ax2.set_position([box.x0, box.y0 + box.height * 0.1, box.width, box.height * 0.9])
box = ax3.get_position()
ax3.set_position([box.x0, box.y0 + box.height * 0.1, box.width, box.height * 0.9])
box = ax5.get_position()
ax5.set_position([box.x0, box.y0 + box.height * 0.2, box.width, box.height * 0.9])
box = ax6.get_position()
ax6.set_position([box.x0, box.y0 + box.height * 0.2, box.width, box.height * 0.9])
#
ax2.text(0.87, 0.9,'(a)', transform=ax2.transAxes)
ax3.text(0.87, 0.9,'(b)', transform=ax3.transAxes)
ax5.text(0.87, 0.9,'(c)', transform=ax5.transAxes)
ax6.text(0.87, 0.9,'(d)', transform=ax6.transAxes)
ax6.legend(bbox_to_anchor=(-0.42, -0.3), loc=9, ncol=3, borderaxespad=0.)
#
fig6.savefig('./14abcd_long_dFdT_dmudT_alt.eps', format='eps', dpi=1000)
plt.show()
```

## Additional tension versus additional linear density¶

In [9]:

```
# fit lines to responses during period of applied heating
# and calculate variations during extended test period without heating
fit_start = 360
fit_stop = 831
fit2_start = 399
fit2_stop = 861
fit5_start = 64
fit5_stop = 273
fit6_start = 172
fit6_stop = 384
fit7_start = 57
fit7_stop = 507

# select whether to use same fit function for both G5b and G5c
use_same_fit = True

# tension
fit = np.polyfit(temp_1[fit_start:fit_stop+1], tens_1[fit_start:fit_stop+1], 1)
print(fit)
if(use_same_fit == True):
    fit2 = fit
else:    
    fit2 = np.polyfit(temp_2x[fit2_start:fit2_stop+1], tens_2x[fit2_start:fit2_stop+1], 1)
print(fit2)
fit_fn = np.poly1d(fit)
add_tens_1 = tens_1 - fit_fn(temp_1)
fit_fn = np.poly1d(fit2)
add_tens_2 = tens_2 - fit_fn(temp_2)
add_tens_3 = tens_3 - fit_fn(temp_3)
add_tens_4 = tens_4 - fit_fn(temp_4)
# G5c at 235 Hz
fit5 = np.polyfit(temp_5[fit5_start:fit5_stop+1], tens_5[fit5_start:fit5_stop+1], 1)
print(fit5)
fit_fn = np.poly1d(fit5)
add_tens_5 = tens_5 - fit_fn(temp_5)
# G5c at 288 Hz
fit6 = np.polyfit(temp_6[fit6_start:fit6_stop+1], tens_6[fit6_start:fit6_stop+1], 1)
print(fit6)
fit_fn = np.poly1d(fit6)
add_tens_6 = tens_6 - fit_fn(temp_6)
# G5c at 306 Hz
fit7 = np.polyfit(temp_7[fit7_start:fit7_stop+1], tens_7[fit7_start:fit7_stop+1], 1)
print(fit7)
fit_fn = np.poly1d(fit7)
add_tens_7 = tens_7 - fit_fn(temp_7)

# linear density
fit = np.polyfit(temp_1[fit_start:fit_stop+1], mu_1[fit_start:fit_stop+1], 1)
print(fit)
if(use_same_fit == True):
    fit2 = fit
else:    
    fit2 = np.polyfit(temp_2x[fit2_start:fit2_stop+1], mu_2x[fit2_start:fit2_stop+1], 1)
print(fit2)
fit_fn = np.poly1d(fit)
add_mu_1 = mu_1 - fit_fn(temp_1)
fit_fn = np.poly1d(fit2)
add_mu_2 = mu_2 - fit_fn(temp_2)
add_mu_3 = mu_3 - fit_fn(temp_3)
add_mu_4 = mu_4 - fit_fn(temp_4)
# G5c at 235 Hz
fit5 = np.polyfit(temp_5[fit5_start:fit5_stop+1], mu_5[fit5_start:fit5_stop+1], 1)
print(fit5)
fit_fn = np.poly1d(fit5)
add_mu_5 = mu_5 - fit_fn(temp_5)
# G5c at 288 Hz
fit6 = np.polyfit(temp_6[fit6_start:fit6_stop+1], mu_6[fit6_start:fit6_stop+1], 1)
print(fit6)
fit_fn = np.poly1d(fit6)
add_mu_6 = mu_6 - fit_fn(temp_6)
# G5c at 306 Hz
fit7 = np.polyfit(temp_7[fit7_start:fit7_stop+1], mu_7[fit7_start:fit7_stop+1], 1)
print(fit7)
fit_fn = np.poly1d(fit7)
add_mu_7 = mu_7 - fit_fn(temp_7)

# plot
plt.figure(figsize=(6,4.5))
plt.plot([-1,1],[0,0],'k--',lw=1)  # Zero reference
plt.plot([0,0],[-100,100],'k--',lw=1)  # Zero reference
#
# additional tension vs additional linear density
plt.plot(add_mu_1, add_tens_1, 'g', label='G5b @ 324 Hz')
plt.plot(add_mu_2, add_tens_2, 'lime', label='G5c @ 324 Hz')
plt.plot(add_mu_3, add_tens_3, 'brown', label='G5c @ 324 Hz, reduced RH')
plt.plot(add_mu_4, add_tens_4, 'brown')
plt.plot(add_mu_5, add_tens_5, 'magenta', label='G5c @ 235 Hz')
plt.plot(add_mu_6, add_tens_6, 'orange', label='G5c @ 288 Hz')
plt.plot(add_mu_7, add_tens_7, 'red', label='G5c @ 306 Hz')
# axes
plt.legend(loc='lower right', ncol = 1, framealpha=1)
plt.axis([-0.25,0.2,-50,15])
plt.xlabel('Additional linear density (g/m$^3$)')
plt.ylabel('Additional tension (N)')
plt.tight_layout()  
plt.savefig('./15_long_addF_vs_addmu.eps', format='eps', dpi=1000)
plt.show()
```

```
[ -2.50708629e-01   2.70573854e+02]
[ -2.50708629e-01   2.70573854e+02]
[  -0.45743285  157.68207428]
[ -1.66300594e-01   2.15335864e+02]
[  -0.25355648  243.14418607]
[  8.02543847e-04   2.52708873e+00]
[  8.02543847e-04   2.52708873e+00]
[ -2.40350501e-03   2.74852511e+00]
[  2.21620221e-03   2.51974345e+00]
[  8.95182857e-04   2.54387209e+00]
```
